# Randomized trial of stopping or continuing ART among postpartum women with pre-ART CD4 ≥ 400 cells/mm^3 ^

**DOI:** 10.1371/journal.pone.0176009

**Published:** 2017-05-10

**Authors:** Judith S. Currier, Paula Britto, Risa M. Hoffman, Sean Brummel, Gaerolwe Masheto, Esau Joao, Breno Santos, Linda Aurpibul, Marcelo Losso, Marie F. Pierre, Adriana Weinberg, Devasena Gnanashanmugam, Nahida Chakhtoura, Karin Klingman, Renee Browning, Anne Coletti, Lynne Mofenson, David Shapiro, Jose Pilotto, for the 1077HS PROMISE Team

**Affiliations:** 1 Division of Infectious Diseases, David Geffen School of Medicine, University of California, Los Angeles, Los Angeles, California, United States of America; 2 Center for Biostatistics in AIDS Research, T.H. Chan School of Public Health, Harvard University, Cambridge, Massachusettes, United States of America; 3 Botswana Harvard AIDS Partnership, Gabarone, Botswana; 4 Hospital Federal dos Servidores do Estado, Rio de Janeiro, Brazil; 5 Hospital Conceicao, Porto Alegre, Brazil; 6 Research Institute for Health Sciences, Chiang Mai University, Chiang Mai, Thailand; 7 HIV Unit, Hospital J.M. Ramos Meija, Buenos Aires, Argentina; 8 Centres GHESKIO, Port-au-Prince, Haiti; 9 University of Colorado Denver, Aurora, Colorado, United States of America; 10 NICHD, Washington, D.C., United States of America; 11 NIAID, NIH, Washington, D.C., United States of America; 12 Science Facilitation, FHI360, Durham, North Carolina, United States; 13 Elizabeth Glaser Pediatric AIDS Foundation, Washington, D.C., United States of America; 14 Laboratório de AIDS & Imunologia Molecular, Instituto Oswaldo Cruz-FIOCRUZ, Rio de Janeiro, Brazil; Azienda Ospedaliera Universitaria di Perugia, ITALY

## Abstract

**Background:**

Health benefits of postpartum antiretroviral therapy (ART) for human immunodeficiency virus (HIV) positive women with high CD4+ T-counts have not been assessed in randomized trials.

**Methods:**

Asymptomatic, HIV-positive, non-breastfeeding women with pre-ART CD4+ T-cell counts ≥ 400 cells/mm^3^ started on ART during pregnancy were randomized up to 42 days after delivery to continue or discontinue ART. Lopinavir/ritonavir plus tenofovir/emtricitabine was the preferred ART regimen. The sample size was selected to provide 88% power to detect a 50% reduction from an annualized primary event rate of 2.07%. A post-hoc analysis evaluated HIV/AIDS-related and World Health Organization (WHO) Stage 2 and 3 events. All analyses were intent to treat.

**Results:**

1652 women from 52 sites in Argentina, Botswana, Brazil, China, Haiti, Peru, Thailand and the US were enrolled (1/2010-11/2014). Median age was 28 years and major racial categories were Black African (28%), Asian (25%) White (15%). Median entry CD4 count was 696 cells/mm^3^ (IQR 575–869), median ART exposure prior to delivery was 19 weeks (IQR 13–24) and 94% had entry HIV-1 RNA < 1000 copies/ml. After a median follow-up of 2.3 years, the primary composite endpoint rate was significantly lower than expected, and not significantly different between arms (continue arm 0.21 /100 person years(py); discontinue 0.31/100 py, Hazard ratio (HR) 0.68, 95% CI: 0.19, 2.40). WHO Stage 2 and 3 events were significantly reduced with continued ART (2.08/100 py vs. 4.36/100 py in the discontinue arm; HR 0.48, 95%CI: 0.33, 0.70). Toxicity rates did not differ significantly between arms. Among women randomized to continue ART, 189/827 (23%) had virologic failure; of the 155 with resistance testing, 103 (66%) failed without resistance to their current regimen, suggesting non-adherence.

**Conclusions:**

Overall, serious clinical events were rare among young HIV-positive post-partum women with high CD4 cell counts. Continued ART was safe and was associated with a halving of the rate of WHO 2/3 conditions. Virologic failure rates were high, underscoring the urgent need to improve adherence in this population.

**Trial registration:**

ClinicalTrials.gov NCT00955968

## Introduction

Over the past two decades, considerable progress has been made in the prevention of perinatal HIV transmission[1,2]. Strategies for the prevention of perinatal transmission among asymptomatic women with early stage HIV infection need to balance the impact of preventing transmission and optimizing infant outcomes with preserving maternal health. Concerns about high rates of maternal morbidity postpartum[3] and health risks of discontinuing antiretroviral therapy (ART) emanating from the Strategies for Management of Antiretroviral Therapy(SMART) study, coupled with strong evidence demonstrating a reduction in the risk of HIV transmission during the use of ART[4] and the clinical benefit from ART among non pregnant women and men with higher CD4 + T-cell counts[5] has led to the evolution of strategies for perinatal HIV transmission that favor the continuous use of triple drug ART for life (herein referred to as ART), also known as Option B+[6]. Concerns about maternal toxicity, possible risks for premature delivery during longer term fetal exposure to ART, challenges to maternal adherence during the postpartum period leading to antiretroviral resistance, and the lack of randomized clinical trial data evaluating the benefits of postpartum ART in women with higher CD4+ T-cell counts have led others to advocate for the rigorous evaluation of the use of short term ART interventions among pregnant women with higher CD4 + T-cells[7–10].

The “HAART Standard” (HS) component of the Promoting Maternal and Infant Safety Everywhere (PROMISE) trial (NCT00955968) was designed to examine the risks and benefits of continued ART compared to stopping ART after delivery and re-initiating treatment when CD4 + T-cell counts fell below 350 cells/mm^3^ among non-breastfeeding women with CD4 + T-cell counts ≥ 400 cells/mm^3^ in the setting of a randomized clinical trial. The trial was planned for settings where ART was the standard of care for the prevention of perinatal HIV transmssion in 2009.

## Materials and methods

The “HAART Standard” component of the PROMISE studies was conducted by the International Maternal, Pediatric Adolescent AIDS Clinical Trials Network (IMPAACT) in collaboration with the AIDS Clinical Trials Network (ACTG). The trial was a randomized strategy trial conducted among clinically stable HIV-infected pregnant women, antiretroviral-naïve except for prior use in pregnancy, without other indications for ART, who received ART during pregnancy for the purpose of preventing perinatal HIV transmission. Women ≥ 18 years of age or who had attained the minimum age of independent consent as defined by the local institutional review board were eligible to enroll if they had documentation of a CD4 + T-cell count of ≥400 cells/mm^3^ within the 120 days prior to the start of ART during the current pregnancy and evidence that CD4 + T-cell count remained ≥ 400 cells/mm^3^ during the 45 days prior to entry while on ART. Participants could not have a clinical indication for ART, including any WHO clinical stage 3 or 4 condition, or any clinically significant illness within 30 days prior to entry. The study was approved by the institutional review board or ethics committee at each participating site and written informed consent was obtained from all participants. During the trial as new guidelines were released with higher CD4 + T-cell count threshholds for ART eligibility, participants were informed and re-consented.

### Study design

This open-label randomized clinical trial evaluated two strategies for the management of ART among postpartum women within 42 days after delivery: continuing ART or discontinuing ART and restarting when clinically indicated. The study was conducted at sites in Argentina, Botswana, Brazil, China, Haiti, Peru, Thailand and the United States. In step 1 of the trial, participants were randomized in a 1:1 ratio to either continue or discontinue ART by a web-based, central randomization system on the IMPAACT Data Management Center’s portal which used permuted block allocation (block size 4) with stratification by screening CD4+ cell count (400–499 versus 500–749 versus ≥ 750 cells/mm^3^) and dynamic balancing by study site[11]. A site staff member enrolled participants by answering eligibility and stratification questions in the randomization system, and if passed, the system randomized the participant and generated a coded study identification number which the site pharmacist compared to a master list and dispensed the corresponding study drug.

Participants randomized to discontinue ART in step 1 entered step 2 and restarted ART if they met one of the following criteria; 1) developed an AIDS-defining/WHO Stage 4 illness, 2) had a confirmed CD4+ T-cell count <350 cells/mm^3^, 3) developed a clinical condition (other than pregnancy) considered an indication for ART by country-specific guidelines during the period of January 2010- July 2015 or 4) otherwise required ART as determined in consultation with the study clinical management committee. Women in step 1 or step 2 entered step 3 if they developed a medical indication to change ART.

The preferred study-supplied ART regimen was lopinavir/ritonavir (LPV/RTV) plus fixed dose combination emtricitabine/tenofovir (FTC/TDF). This regimen was chosen because it was the preferred regimen for use in pregnancy by the Department of Health and Human Services (DHHS) guidelines at the time the study was designed[12]. Additional study-supplied antiretrovirals (ARVs) included fixed dose combination lamivudine/zidovudine (3TC/ZDV), lamivudine (3TC), zidovudine (ZDV), tenofovir disoproxil fumarate (TDF), fixed dose combination emtricitabine/tenofovir disoproxil fumarate/rilpivirine (FTC/TDF/RPV), atazanavir (ATV), raltegravir (RAL), and ritonavir (RTV). Study clinicians in conjunction with participants were allowed to determine the optimal drug combination for each participant. Regimens not provided by the study were allowed if they included three or more agents from two or more classes of ART.

Participants were seen for clinical and safety evaluations at four weeks and at 12 weeks, and every 12 weeks thereafter. If a change in treatment was required for disease progression or due to toxicity, participants were seen at four weeks after ART regimen change and then returned to the every 12 week schedule. HIV viral load was used to maximize the benefits of ART and to determine when treatment should be changed. HIV-1 RNA was evaluated in real time at baseline and among those randomized to continue ART, at weeks four and 12 after entry andevery 12 weeks thereafter. A viral load was also obtained at every step change.

### Study endpoints

The primary composite endpoint included death from any cause, AIDS-defining illness, and serious non-AIDS-defining cardiovascular, serious renal, and hepatic events. The qualifying illnesses and conditions corresponding to the primary endpoints are as follows: AIDS-defining illness refers to the WHO Clinical Stage 4 conditions; cardiovascular endpoints include myocardial infarction, stroke or coronary artery disease requiring revascularization; renal endpoints included end stage renal disease; and hepatic endpoints include decompensated liver disease (cirrhosis). Secondary endpoints reported here include: 1) composite endpoint of HIV/AIDS related events (defined as WHO Stage 4 conditions, pulmonary tuberculosis and serious bacterial infections) or death, 2) composite endpoint of HIV/AIDS-related event or WHO Clinical Stage 2 or 3 event, 3) safety, and 4) HIV-1 viral resistance to ART at the time of virologic failure. The safety endpoint is a composite of a grade 3 or 4 sign or symptom, or grade 2, 3, or 4 hematology or chemistry result, whichever came first. Post-hoc analysis included comparing WHO Stage 2 and 3 events in those randomized to continue versus discontinue ART.

### Statistical analysis

The planned sample size of 2000 was selected to provide 90% power to detect a 50% reduction from an annualized primary event rate of 2.07% in the discontinue ART group. Analyses used the principle of intention-to-treat and included all women randomized. Comparisons between treatment groups were based on log rank tests and Cox regression models for estimation of treatment effect sizes[13]. The distributions of time until the first event occurred were summarized using Kaplan-Meier plots[14]. Point estimates, p-values, and 95% confidence intervals were not adjusted for interim efficacy analyses due to the conservative O’Brien-Fleming error spending function used[15]. The analyses presented here reflect follow-up until July 7, 2015, when participants were informed about the Strategic Timing of AntiRetroviral Treatment (START) trial results[16] and all participants were offered ART. A two-sided p-value less than 0.05 was considered statistically significant. Statistical analyses were performed using Statistical Analysis System (SAS) software version 9.4 (SAS Institute).

### Interim monitoring

The study was reviewed by an independent National Institute of Allergy and Infectious Diseases (NIAID)-sponsored Data Safety and Monitoring Board (DSMB). The DSMB reviewed annual interim analyses of safety, study logistics, and an assessment of the accuracy of the assumed annualized primary endpoint rate, and two interim analyses of efficacy and futility. Due to slow accrual, the DSMB approved curtailing enrollment at a minimum of 1630 participants in November 2014. The reduced sample size retained 88% power to detect the original effect size due to the additional follow-up time contributed by those enrolled early in the trial. In November 2015, the DSMB recommended that the primary analysis should include only follow-up until July 7, 2015, when all participants were offered ART, and that the study team should be unblinded so that they could begin the primary analysis and publish the results.

## Results and discussion

### Study patients

From January 2010 through November 2014, 1653 women at 52 were randomly assigned to continue or discontinue ART within 42 days after delivery. One woman withdrew consent and discontinued study follow-up on the day she was randomized and was not included in the final analysis. The two study groups were well balanced at entry (Table 1). The median age was 28 years and major racial categories were Black African (28%), Asian (25%), and White (15%). Median entry CD4+ T-count was 696 cells/mm^3^, median ART exposure prior to delivery was 19 weeks (IQR 13–24) and 94% had an entry HIV-1 RNA < 1000 copies/ml. The median follow-up time was 2.3 years (range: 10, 279 weeks) and duration of follow-up did not differ significantly across the randomization arms.

**Fig 1:**
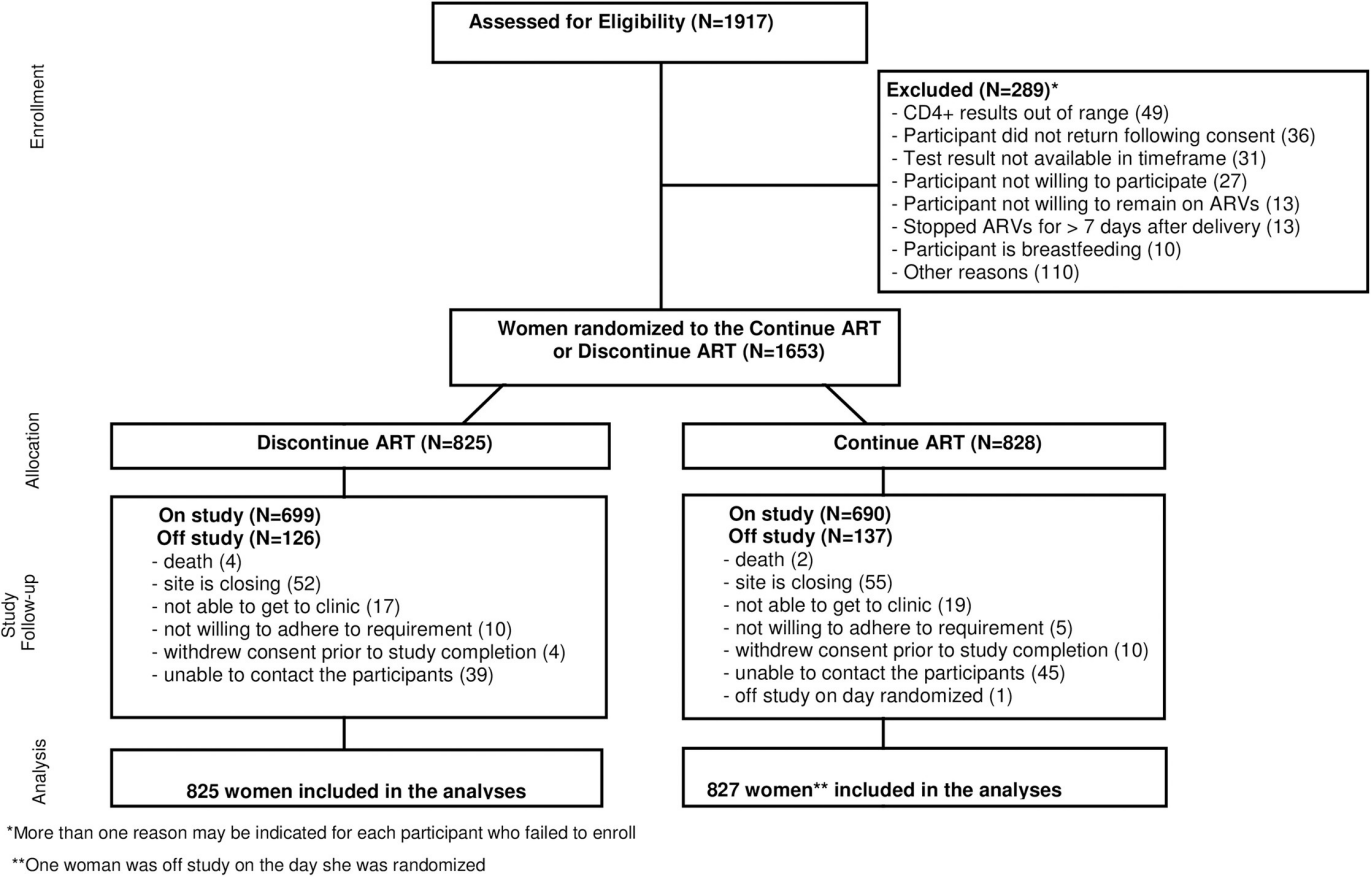
CONSORT Flow Diagram.

Of the 1652 women included in the analysis, 149 women (9%) discontinued follow-up prematurely. An additional 107 women (7%) were taken off study due to site closure. A total of 136 women in the continue arm and 126 in the discontinue arm were off study. Study participant disposition is shown in Fig 1. Reasons participants went off study are summarized in S1 Fig.

**Table 1:** Baseline Characteristics.

Characteristic		Continue ART (N = 827)	Discontinue ART (N = 825)	Total (N = 1652)
Country	Argentina Botswana Brazil China Haiti Peru Thailand USA	20(2%) 230 (28%) 259 (31%) 52 (6%) 32 (4%) 6 (1%) 156 (19%) 72 (9%)	25 (3%) 227 (28%) 255 (31%) 52 (6%) 29 (4%) 9 (1%) 151 (18%) 77 (9%)	45(3%)
Race^a^	Asian	209 (25%)	203 (25%)	412 (25%)
	Black or African American	58 (7%)	58 (7%)	116 (7%)
	White	123 (15%)	127 (15%)	250 (15%)
	American Indian	1 (0%)	1 (0%)	2 (0%)
	Alaska Native	0 (0%)	1 (0%)	1 (0%)
	Black African	230 (28%)	227 (28%)	457 (28%)
	Black of African origin	77 (9%)	70 (8%)	147 (9%)
	Mestizo	6 (1%)	8 (1%)	14 (1%)
	Mixed Black	73 (9%)	76 (9%)	149 (9%)
	Mixed Native	0 (0%)	4 (0%)	4 (0%)
	Native (native Brazilian-Xavante/Kaigang/Guarani etc)	1 (0%)	1 (0%)	2 (0%)
	Other	31 (4%)	31 (4%)	62 (4%)
	Subject does not know	4 (0%)	2 (0%)	6 (0%)
	Race not available to clinic	14 (2%)	16 (2%)	30 (2%)
BMI at Entry (kg/m^2^)	N	812	801	1613
	Median (Q1-Q3)	24.4 (21.7–28.0)	24.6 (21.8–28.3)	24.5 (21.8–28.1)
	# missing	15	24	39
WHO Stage at Entry	Clinical Stage I	810 (98%)	811 (99%)	1,621 (98%)
	Clinical Stage II	16 (2%)	11 (1%)	27 (2%)
	Clinical Stage III	1 (0%)	1 (0%)	2 (0%)
	# missing	0	2	2
Duration of ART prior to study entry, weeks	N	827	825	1652
	Median (Q1-Q3)	17 (11–23)	17 (11–23)	17 (11–23)
	# missing	0	0	0
ART regimen prior to Entry	HAART including Boosted PI	620 (75%)	612 (74%)	1,232 (75%)
	HAART including Non-boosted PI	12 (1%)	16 (2%)	28 (2%)
	HAART including NNRTI [EFV]	180 (22%)	172 (21%)	352 (21%)
	HAART including NNRTI [NVP]	4 (0%)	8 (1%)	12 (1%)
	HAART including NNRTI [RPV]	1 (0%)	1 (0%)	2 (0%)
	HAART including NNRTI and PI	1 (0%)	0 (0%)	1 (0%)
	Three or more NRTIs	3 (0%)	11(1%)	14 (1%)
Hepatitis B surface antigen positive	Positive	29 (4%)	28 (3%)	57 (3%)
	Negative	771 (93%)	768 (93%)	1,539 (93%)
	Indeterminate	1 (0%)	0 (0%)	1 (0%)
	Not obtained, Hep B antibody +ve	20 (2%)	23 (3%)	43 (3%)
	Not obtained	6 (1%)	6 (1%)	12 (1%)
Hepatitis B surface antibody positive	Positive	263 (32%)	248 (30%)	511 (31%)
	Negative	424 (51%)	425 (52%)	849 (51%)
	Indeterminate	1 (0%)	2 (0%)	3 (0%)
	Not obtained, Hep B antigen +ve or -ve	138 (17%)	148 (18%)	286 (17%)
	Not obtained	1 (0%)	2 (0%)	3 (0%)
Screening CD4+ cell count on ART (cells/mm^3^)	N	827	825	1652
	Median (Q1-Q3)	696 (575–870)	695 (575–868)	696 (575–869)
	# missing	0	0	0
Pre-ART CD4+ cell count (cells/mm^3^)	N	827	825	1652
	Median (Q1-Q3)	550 (461–682)	548 (463–677)	549 (462–680)
	# missing	0	0	0
Plasma Viral Load at Entry (copies/ml)	<400	744 (91%)	742 (91%)	1,486 (91%)
	400–1000	30 (4%)	24 (3%)	54 (3%)
	1000- <10000	32 (4%)	29 (4%)	61 (4%)
	10000 - <100000	15 (2%)	24 (3%)	39 (2%)
	> = 200000	1 (0%)	0 (0%)	1 (0%)
	# missing	5	6	11

^a^ For Race: Other—The 62 were coded as having a Race/Ethnicity of Hispanic (regardless of race).

### Use of antiretroviral therapy

Of the women enrolled, 1,266 (77%) were receiving a protease inhibitor (PI) containing ART regimen just prior to randomization. Ninety percent of the study participants randomized to continue ART were treated with a ritonavir boosted protease inhibitor regimen: LPV/RTV in 76%, atazanavir/ritonavir in 13% and other PIs in the remaining 1%. Non-nucleoside reverse transcriptase inhibitor (NNRTI) based ART was used by 8% of participants with efavirenz accounting for 7% overall. Integrase inhibitor-based ART was used by <1% of study participants. Boosted PI were predominantly used across all countries with the exception of Haiti, where approximately half of the participants used efavirenz-based ART. Post randomization ART usage among those randomized to continue ART is summarized in Table 2.

**Table 2:** ART use within 72 hours after randomization for the continue ART arm.

	Continue ART (N = 825)	
**Antiretroviral Regimen**		
**Boosted Protease Inhibitor Regimens**	**744**	**(90%)**
Lopinavir/r	625	
Atazanvir/r	104	
Darunavir/r	10	
Fosamprenavir/r	3	
Indinavir/r	2	
**Non-Boosted Protease Inhibitor Regimens**	**8**	**(<1%)**
Atazanvir	6	
Nelfinavir	2	
**Non-Nucleoside Reverse Transcriptase Inhibitor Regimens**	**65**	**(8%)**
Efavirenz	56	
Rilpivirine	8	
Nevirapine	1	
**Nucleoside Reverse Transcriptase Regimens**	**2**	**(<1%)**
ZDV+3TC+ABC	2	
**Intergrase Inhibitor Based Regimens**	**4**	**(<1%)**
Raltegravir	2	
Elvitegravir/Cobicistat	2	
**No Follow-up after the Entry Visit**	**2**	

### Adherence to randomization strategy

As of July 7, 2015 ART had been started in 102 (12%) of the discontinue group prior to reaching a CD4 count of 350 cells/mm^3^ (5% per year). In contrast 119 (15%) of those randomized to continue ART prematurely discontinued ART (6.5% per year).

### Study endpoints

After a median follow-up of 2.3 years, ten participants experienced a primary composite endpoint event with an overall rate of 0.26%, significantly lower than expected and not significantly different between arms: 0.21 per 100 person years (py) in the continue arm, compared to 0.31 per 100 py in the discontinue arm, HR 0.68 (95% CI: 0.19, 2.40; p 0.54). The events are described in Table 3.

**Table 3:** Clinical Endpoints.

Endpoint (time to first event)	Continue ART no. no./ 100 py		Discontinue ART no. no./ 100 py		Hazard Ratio^c^	p-value
Primary Composite Endpoint (first of AIDS,Death,or Serious Non-AIDS Cardio/Renal/Hepatic	4	0.21	6	0.31	0.68 (0.19, 2.40)	0.54
AIDS Defining (WHO 4)	2	0.10	3	0.15	0.67 (0.11, 4.01)	0.66
Death (including after AIDS event)	2	0.10	4	0.20	0.52 (0.09, 2.81)	0.44
Secondary Endpoints						
Composite Endpoint of HIV/AIDS related events^a^ and WHO stage 2–3 events	57	3.09	97	5.37	0.58 (0.42, 0.80)	<0.001
HIV/AIDS related events						
Single Bacterial Pneumonia	10	0.52	14	0.73	0.72 (0.32, 1.62)	0.42
Grade 4 Bacterial infections	5	0.26	2	0.10	2.52 (0.49,12.98)	0.25
Bacterial infections resulting in hospitalization	14	0.73	14	0.73	1.00 (0.48, 2.11)	0.99
Bacterial infections causing death	0	NA	0	NA	NA	NA
Composite Endpoint of HIV/AIDS related event or death	27	1.42	30	1.57	0.91 (0.54, 1.52)	0.71
WHO 2–3 events (including WHO 2–3 diagnoses after oserved WHO IV events)	39	2.08	80	4.36	0.48 (0.33, 0.70)	<0.001
Toxicity Events^b^						
Grade 2 Toxicity and above	260	18.4	234	15.6	0.86 (0.72, 1.03)	0.10
Grade 3 and 4 Toxicity	188	12.0	161	9.8	1.13 (0.86, 1.49)	0.08

^a^HIV/AIDS related events refers to the WHO Clinical Stage 4 illnesses, pulmonary tuberculosis, and other serious bacterial infections.

^b^Toxicity Event is defined as composite of the time to the first grade 3 or 4 sign or symptom, or grade 2, 3, or 4 hematology or chemistry event, whichever comes first.

^c^ The Hazard Ratio is for the Continue ART arm as compared to the Discontinue ART arm.

**Fig 2:**
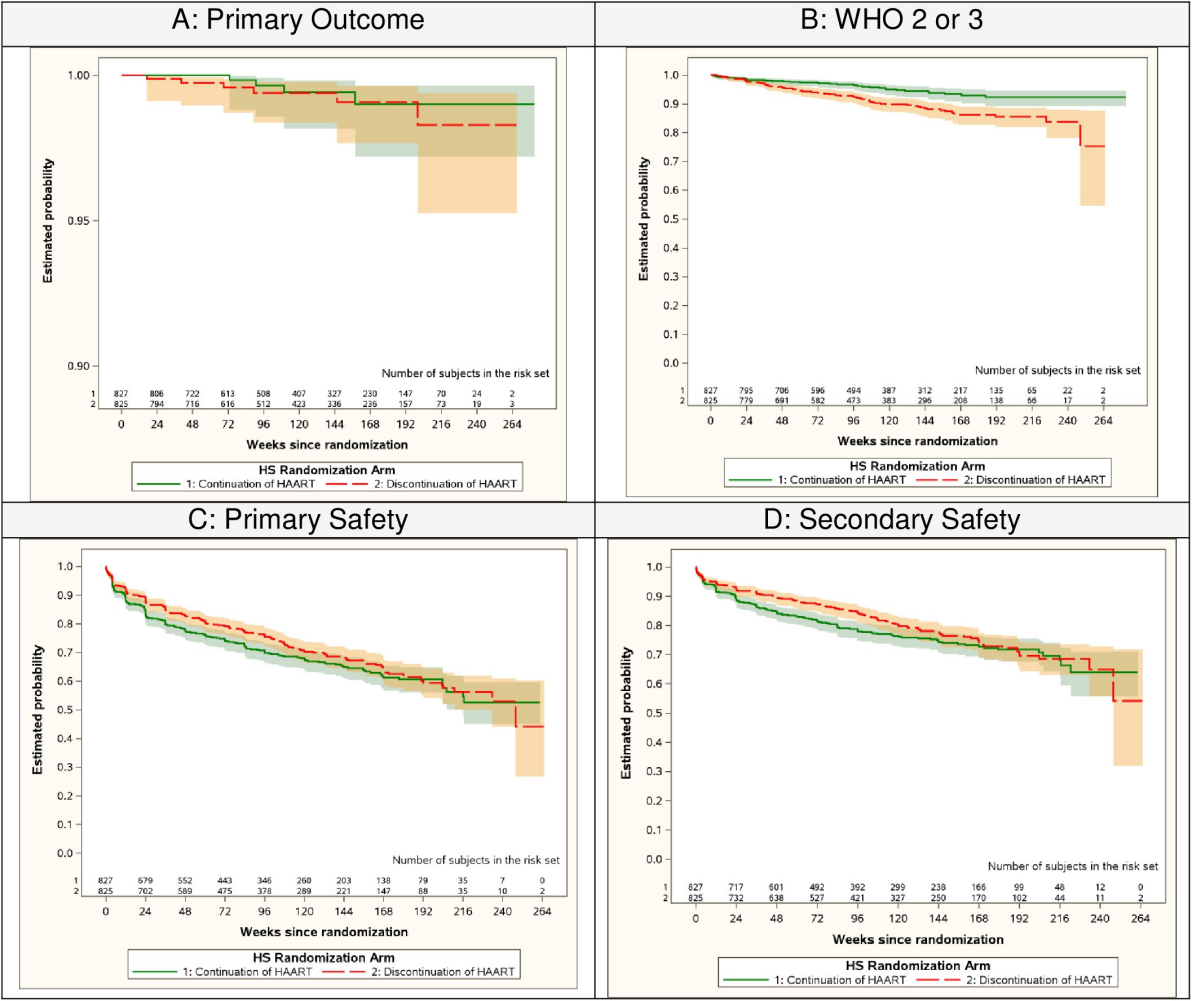
Survival Curves for the Primary and Secondary Endpoints. A) Composite endpoint of the first AIDS-defining illness, serious non-AIDS defining cardiovascular, renal or hepatic event or death. B) Composite endpoint of the first WHO 2 or 3 event. C) Composite of first grade 3 or 4 sign or symptom, or grade 2,3, or 4 hematology or chemistry event. D) Composite of the first grade 3 or 4 sign or symptom, or hematology or chemistry event.

Participants randomized to continue ART experienced a significantly lower rate of the pre-specified secondary endpoint that included a range of clinical outcomes (HIV/AIDS related event or WHO stage 2–3 event). For this endpoint, 57 women (3.09 events per 100 py) in the continue arm, compared to 97 (5.37 events per 100 py) in the discontinue arm experienced an event, with a hazard ratio of 0.58 (95% CI: 0.42, 0.80; p < 0.001). This difference was primarily due to differences in rates of WHO Stage 2 and 3 events: 2.08 events per 100 py in the continue arm vs 4.36 events per 100 py in the discontinue arm, with a HR of 0.48 (95% CI: 0.33, 0.70; p< 0.001). The distributions of the time until the first event occurred are shown in Fig 2 for the primary and secondary efficacy and safety endpoints.

**Fig 3:**
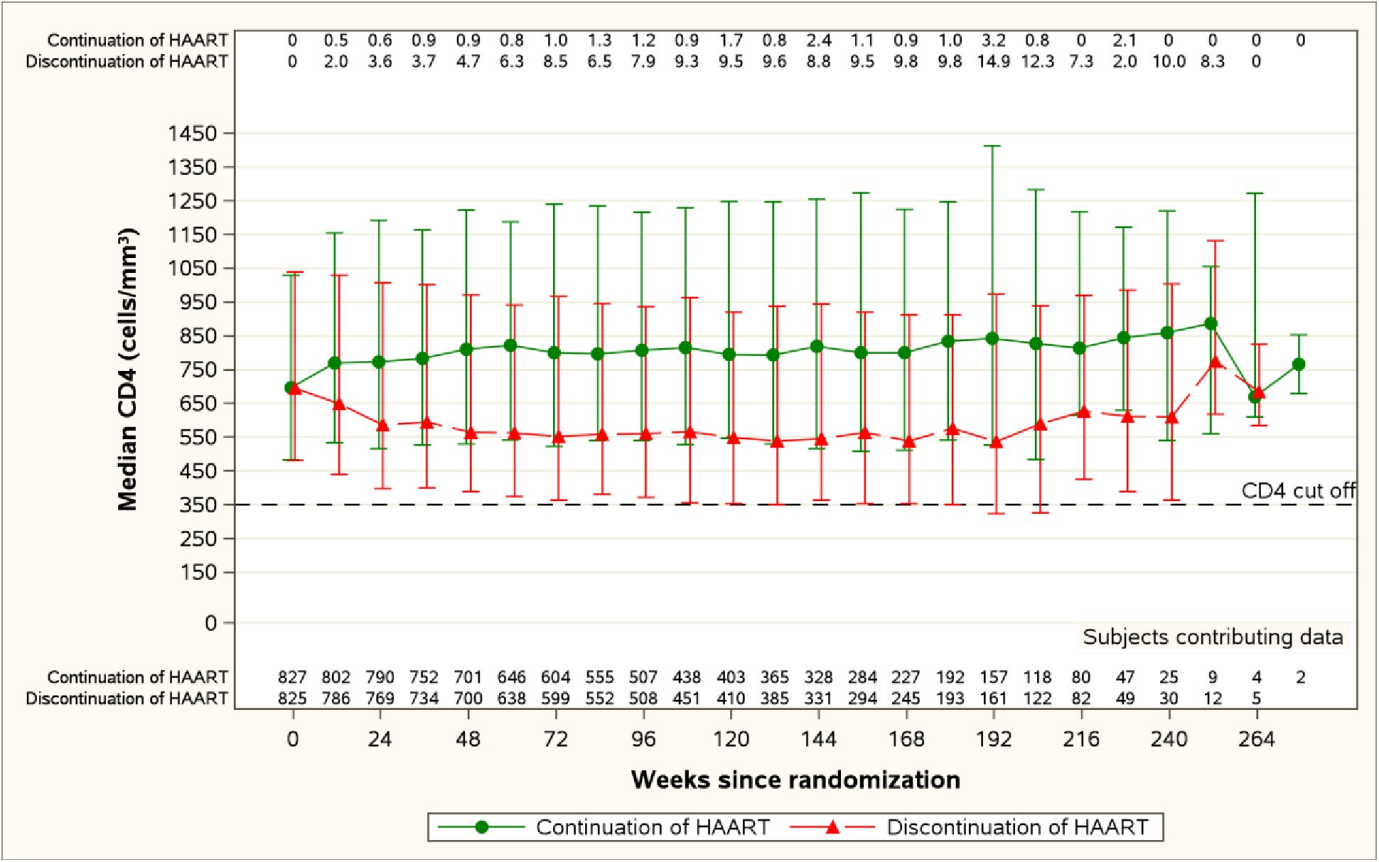
CD4 Cell Counts Over time (Median, 10^th^ and 90^th^ percentile, and the proportion <350 cells/mm). The median CD4 cell counts in the continue (green) and discontinue (red) arms are show at each follow-up visit. The proportion with a CD4 count below 350 cells/mm3 are shown across the top of the figure at each timepoint.

Trajectories of CD4 + T-cell counts over time are shown in Fig 3. The percentage of participants in the discontinue arm with CD4+ T-cell counts below 350 cells/mm^3^ was less than <10% throughout follow-up, with the exception of week 192 when it rose transiently to 15%. In contrast, among those randomized to continue ART, the percentage of participants whose CD4+ T-cell count had dropped below 350 cells was less than 4% throughout follow-up.

The primary safety endpoint of selected grade 2 and grades 3 and 4 hematologic and chemistry abnormalities occurred in 260 of the continue arm participants (18.4 per 100 py) and in 234 of the discontinue arm (15.6 per 100 py) yielding a HR of 1.16 (95% CI: 0.97, 1.38 p = 0.10). A complete listing of the specific laboratory adverse events by treatment arm is included in Table 4. Notably, grade 2–4 renal events occurred in less than one percent of the study population. Thrombocytopenia was more common in the discontinue group (18 events) compared to the continue group (four events). When grade 3 and 4 adverse event rates were compared between treatment arms, the rates were numerically but non-significantly higher in the continue group compared to the discontinue group (12.0 per 100 py. vs. 9.8 per 100py respectively, HR 1.20, 95% CI: 0.98, 1.49; p = 0.08), with transaminase elevations and cholesterol elevations accounting for the higher rate in the continue group.

**Table 4:** Laboratory Adverse Events^a^.

Toxicities	Continue ART (n = 827) Grade				Discontinue ART (n = 825) Grade			
	2	3	4	Total	2	3	4	Total
**Any Chemistry** **Event**	**24 (3%)**	**81 (10%)**	**22 (3%)**	**127 (15%)**	**18 (2%)**	**65 (8%)**	**15 (2%)**	**98(12%)**
**Any Hepatic**	26 (3%)	13 (2%)	7 (< 1%)	46 (6%)	20 (2%)	12 (2%)	4 (< 1%)	36 (4%)
ALT	16	10	7	33	21	12	2	35
AST	12	10	5	27	10	5	3	18
Indirect Bilirubin	5	2	0	7	0	0	0	0
**Renal (Creatinine)**	**5 (<1%)**	**0(0%)**	**0 (0%)**	**5 (<1%)**	**1 (< 1%)**	**0 (0%)**	**1 (<1%)**	**2 (< 1%)**
**Any General Chemistry**	**0 (0%)**	**25 (3%)**	**13 (2%)**	**38 (5%)**	**0 (0%)**	**24 (3%)**	**8 (1%)**	**32 (4%)**
Phosphorus	0	19	2	21	0	14	0	14
Sodium	0	5	7	12	0	8	5	13
Potassium	0	4	2	6	0	2	2	4
Alkaline Phosphatase	0	0	2	2	0	0	0	0
Bicarbonate	0	0	0	0	0	0	1	1
**Any Metabolic**	**0 (0%)**	**44 (5%)**	**2 (< 1%)**	**46 (6%)**	**0 (0%)**	**30 (4%)**	**2 (< 1%)**	**32 (4%)**
LDL (fasting)	0	36	0	36	0	25	0	25
LDL (non-fasting)	0	1	0	1	0	0	0	0
Total cholesterol (fasting)	0	21	0	21	0	14	0	14
Total cholesterol (non-fasting)	0	1	0	1	0	0	0	0
Glucose (non-fasting)	0	3	0	3	0	3	1	4
Triglycerides (fasting)	0	4	2	6	0	0	1	1
HDL (fasting)	0	0	0	0	0	1	0	1
**Any Endocrine Metabolic**	**0 (0%)**	**4 (<1%)**	**0 (0%)**	**4 (<1%)**	**0 (0%)**	**5(<1%)**	**0(0%)**	**5(<1%)**
Glucose (fasting)	0	4	0	4	0	5	0	5
**Any Hematologic Event**	**68 (8%)**	**25 (3%)**	**10 (1%)**	**98 (12%)**	**71 (9%)**	**26 (3%)**	**14 (2%)**	**111 (14%)**
Platelets	3	0	1	4	14	1	3	18
Hemoglobin	21	7	8	36	15	8	9	32
White Blood Cells	1	0	0	1	4	1	0	5
Absolute Neutrophil Count	43	18	1	62	44	16	2	62

^a^Each participant is counted once at the highest grade for the specific safety event, once for the safety category total, and once for the overall total.

### Virologic failure and HIV-1 drug resistance

Virologic failure among the women randomized to continue ART was defined as two successive measurements of HIV-1 RNA above 1000 copies/ml at or after 24 weeks of ART. A total of 293 women had at least one HIV-1 RNA above 1000 copies/ml, of these 92 re-suppressed on the next measurement and 12 were lost to follow-up, hence 189 (23%) met the definition of virologic failure. Of the 189 with confirmed virologic failure, 156 women had antiretroviral drug resistance testing performed with available results. Fifty-two (33%) women had the presence of at least one mutation (includes polymorphisms) and 36 had evidence of HIV drug resistance defined using the Stanford database (version 6.2). Among the 36 women with drug resistance, 17 had evidence of resistance mutations not selected by the current ART regimen and 19 had evidence of resistance selected by the current ART regimen. If all women who had virologic failure and available resistance data are considered, this translates to 19/156 (12%) overall who failed with resistance to their current ART. Virologic failure with resistance to the current regimen was more common in women failing NNRTI containing regimens (7/9, 78%) than those failing PI containing regimens (11/142, 8%). A summary of ART at the time of virologic failure and resistance is included in Table 5.

**Table 5:** Resistance mutations and ART use.

Class	Regimen Anchor Drug	Number Failed	Any Mutation Present	Any HIV Drug Resistance (%)	Resistance Selected by current ART (%)
Boosted PI + NRTI	LPV/RTV	101	32	18(17.8%)	7(6.9%)
	ATV/RTV	31	9	7(22.6%)	4(12.9%)
	DRV/r	5	1	1(20.0%)	0(0.0%)
	FPV	2	0	0(0.0%)	0(0.0%)
	IDV/r	1	0	0(0.0%)	0(0.0%)
	NFV	1	0	0(0.0%)	0(0.0%)
II	RAL	1	0	0(0.0%)	0(0.0%)
NNRTI	Efavirenz	8	7	7(87.5%)	7(87.5%)
	RPV	1	0	0(0.0%)	0(0.0%)
OTHER	ZDV+3TC	4	2	2(50.0%)	0(0.0%)
	LPV/RTV only	1	1	1(100.0%)	1(100.0%)
TOTAL		156	52	36(23.1%)	19(12.2%)

## Discussion

This is the first large multicenter randomized clinical trial to compare the strategy of stopping or continuing ART in postpartum women with early stage HIV infection. The study demonstrates a low rate of serious clinical events over 2.3 years of postpartum follow-up in both arms. However, continuing ART was associated with a clinical benefit evidenced by a 50% decrease in the rates of WHO 2 and 3 events compared with discontinuing ART. ART was safe with low rates of toxicity in this young population where the majority were receiving a boosted protease inhibitor combined with emtricitabine and tenofovir. These findings demonstrate a very low rate of serious non-AIDS events among young women as compared to rates previously reported among studies that included populations who were older[5,16,17]. There are several factors that may have contributed to these differences: 1) our study participants were younger than those in other studies; 2) their baseline CD4+ T-cell count was higher; 3) our study population exclusively consisted of women, whereas previous studies enrolled mostly men. Importantly, the smaller size of our study and relatively short follow-up reduces our power to detect differences between arms for the primary efficacy endpoint.

These findings help to inform current guidelines for the use of Test and Start and Option B+ globally by highlighting an important challenge associated with this strategy. The relatively high rates of virologic failure among women in the continue ART group underscore the critical need to remove barriers to long-term adherence during the postpartum period. The absence of HIV drug resistance among most participants with virologic failure on ART needs to be interpreted cautiously given that the majority of participants were receiving a boosted PI regimen, where resistance at the time of failure is often not associated with measurable drug resistance and participants can often re-suppress HIV-1 RNA with continuation of the same regimen[18]. However, It is important to highlight that standard genotypic testing, performed by conventional sequencing, does not readily detect variants below 20% of the circulating HIV-1 RNA population. In patients with poor adherence to a boosted PI regimen, deep sequencing may detect minority PI-resistant variants, which likely represent early events in resistance selection[19].

Our data portend higher rates of virologic failure with resistance in settings where efavirenz-based ART regimens are predominant. These findings underscore the importance of evaluating newer and potentially more durable antiretroviral agents such as integrase inhibitors with a higher genetic barrier to drug resistance in pregnant and post-partum women.

The low rate of clinical progression observed in those randomized to discontinue ART postpartum also provides some reassurance for women who cannot become virologically suppressed on currently available ART, due to toxicity or tolerability or, who require observation off ART due to complex multifactorial issues that preclude adherence. Previous observational follow-up studies of postpartum women in African settings identified a low rate of progression to WHO Stage 4 or death among women with CD4+ T-cell counts > 550 cells/mm^3^ and our data extend this finding[20]. These findings may also have implications for the design of cure research studies that include an interruption of treatment to evaluate the impact of an intervention to purge the reservoir by providing insight into the safety of discontinuing ART among younger patients without a history of immunodeficiency. It is important to note that the duration of ART in these participants prior to discontinuation was only 19 weeks on average, however.

The strengths of our study include the randomized design, the conduct in multiple countries including both lower and middle income settings, and rigorous assessments of safety, efficacy and virologic outcomes. Weaknesses include the smaller than planned sample size, lower than expected event rate resulting in reduced power, and the relatively short follow-up. The study also did not follow the infants and cannot provide any information about infant outcomes. Additionally, the use of a boosted PI regimen in our trial, which was based on US Guidelines at the time the trial was designed, may reduce the generalizability of our findings to settings where efavirenz has become the preferred agent for treatment. The efficacy of ART with efavirenz as compared to a boosted PI might be expected to be similar, as was previously demonstrated among pregnant women in Uganda[21], however the consequences of non-adherence with respect to the development of HIV-1 drug resistance when failure occurs may result in more clinically significant drug resistance with efavirenz.

## Conclusion

In conclusion, continuation of ART in the post-partum period is of clinical benefit to women with early HIV infection and preserved CD4 cell counts. Our study highlights the need to improve adherence to long term ART in this vulnerable population. These efforts will be critical in order to reach the collective goal of improving the health of all people with HIV infection and for reducing the rates of new infections.

## Supporting information

S1 CONSORT Diagram Checklist (PDF)

S1 Fig Reasons Participants Went off Study. (TIF)

S1 Protocol (PDF)
