# Supplementary figures and images for: Randomized trial of stopping or continuing ART among postpartum women with pre-ART CD4 ≥ 400 cells/mm3
Source: PLoS One. 2017 May 10;12(5):e0176009. doi: 10.1371/journal.pone.0176009 (PMC5425014; doi:10.1371/journal.pone.0176009)

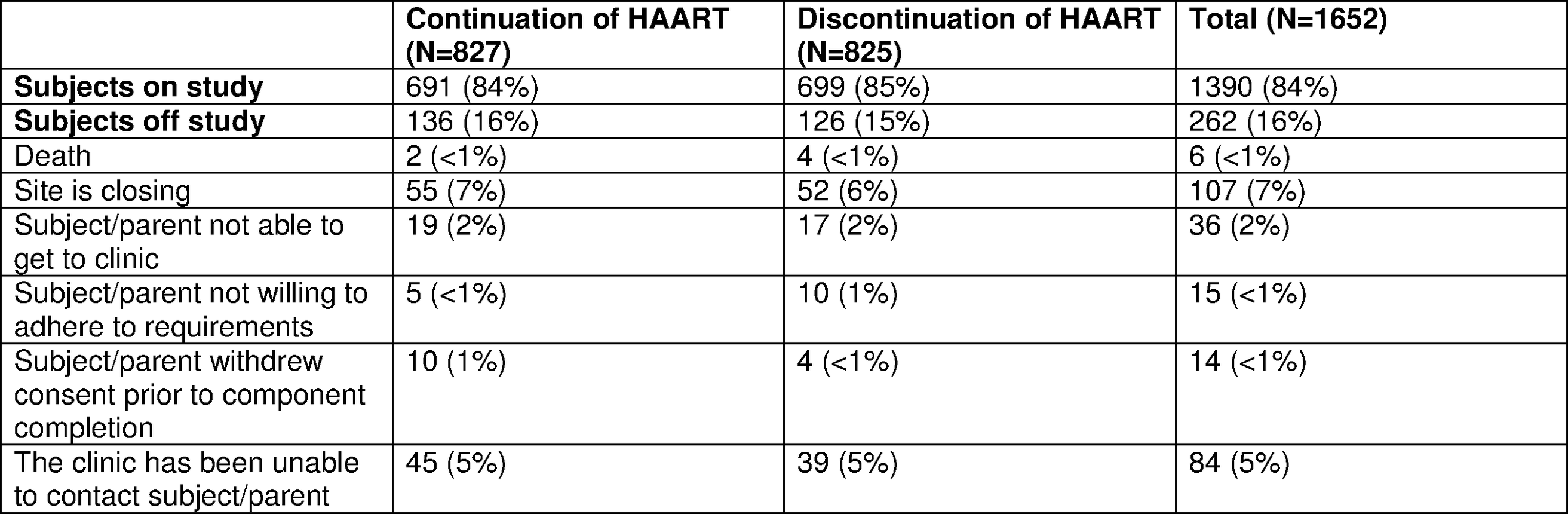

Supplement: S1 Fig — (TIF) [file pone.0176009.s002.tif]
