# Supplementary material for: Randomized trial of stopping or continuing ART among postpartum women with pre-ART CD4 ≥ 400 cells/mm3
Source: PLoS One. 2017 May 10;12(5):e0176009. doi: 10.1371/journal.pone.0176009 (PMC5425014; doi:10.1371/journal.pone.0176009)
Supplement: S1 Protocol — (PDF) [file pone.0176009.s003.pdf]

IMPAACT 1077HS

(DAIDS Document ID 10779)

HAART Standard Version of the PROMISE Study (Promoting Maternal and Infant Survival Everywhere)

A Multicenter, US Domestic and International Trial of the
International Maternal Pediatric Adolescent AIDS
Clinical Trials (IMPAACT) Group

Sponsored by:

The National Institute of Allergy and Infectious Diseases (NIAID)
and
The *Eunice Kennedy Shriver*
National Institute of Child Health and Human Development (NICHD)

Pharmaceutical Support Provided by:

Abbott, Bristol-Myers Squibb, Gilead Sciences, GlaxoSmithKline/ViiV, and Merck and Company, Inc.

**IMPAACT HIV Prevention Science Committee Chairs:**	Patricia Flynn, MD<br>Benjamin Chi, MD
**Protocol Chair:**	Judith S. Currier MD, MS
**Protocol Vice Chairs:**	Arlene Bardeguez, MD, FACOG, MPH<br>Jose Pilotto, MD
**NIAID Medical Officers and Medical Monitor:**	Lawrence Fox, MD, PhD<br>Karin L. Klingman, MD<br>Renee Browning, RN, MSN
**NICHD Medical Officers:**	Lynne Mofenson, MD<br>Heather Watts, MD

FINAL Version 2.0
9 October 2012
PROTOCOL TEAM ROSTER

Chair
Judith S. Currier, MD, MS
UCLA CARE Center
9911 W Pico Blvd., Suite 980
Los Angeles, CA 90035
Phone: (310) 557-1891
Fax: (310) 557-1899
Email: jscurrier@mednet.ucla.edu

Vice Chairs
Arlene Bardeguez, MD, FACOG, MPH
Department of Obstetrics, Gynecology & Women's Health
University of Medicine & Dentistry of New Jersey
185 South Orange Avenue
MSN E 506
Newark, NJ 07103-2714
Phone: (973) 972-5482
Fax: (973) 972-4574
Email: bardegad@umdnj.edu

Jose Henrique Pilotto, MD
Hospital Geral de Nova Iguaçu
Av. Henrique Duque Estrada Mayer 953
Nova Iguaçu – Alto da Posse.
Rio de Janeiro 26030-380
BRAZIL
Phone: 55 21 26673022
Fax: 55 21 26673022
Email: pilotto@unisys.com.br

NICHD Medical Officers
Lynne M. Mofenson, MD
Pediatric, Adolescent & Maternal AIDS Branch/CRMC
National Institute of Child Health & Human Development (NICHD)
National Institutes of Health
6100 Executive Boulevard, Room 4B11
Rockville, MD 20892
Phone: (301) 435-6870
Fax: (301) 496-8678
Email: LM65D@nih.gov
Email: Lynne.Mofenson@nih.hhs.gov

Heather Watts, MD
Pediatric, Adolescent & Maternal AIDS Branch/CRMC
National Institute of Child Health & Human Development (NICHD)
National Institutes of Health
6100 Executive Boulevard, Room 4B11G
Bethesda, MD 20892
Phone: (301) 435-6874
Fax: (301) 496-8678
Email: wattsh@mail.nih.gov

NIAID Medical Officers
Lawrence Fox, MD, PhD
TRP, DAIDS, NIAID, NIH
6700-B Rockledge Drive, MSC 7624
Bethesda, MD 20892
Phone: (301) 402-0129
Fax: (301) 480-4582
Email: lfox@niaid.nih.gov

Karin L. Klingman, MD
TRP, DAIDS, NIAID, NIH
6700-B Rockledge Drive, MSC 7624
Bethesda, MD 20892
Cell Phone: (240) 281-1511
Office Phone: (301) 435-3772
Fax: (301) 480-4582
Email: kklingman@niaid.nih.gov

NIAID Medical Monitor
Renee Browning, RN, MSN
Henry M. Jackson Foundation
NIAID, NIH
Bethesda, MD 20892-7620
Phone: (301) 435-3770
Email: browningr@niaid.nih.gov

Statisticians
Paula Britto, MS
Statistical & Data Analysis Center
Harvard School of Public Health
FXB Room 535A
651 Huntington Avenue
Boston, MA 02115-6017
Phone: (617) 432-2522
Fax: (617) 432-3163
Email: britto@sdac.harvard.edu

Sean Brummel, PhD
Statistical & Data Analysis Center
Harvard School of Public Health
Boston, MA 02115
Phone: (617) 423-1197
Fax: (617) 432-3163
Email: sbrummel@sdac.harvard.edu

David Shapiro, PhD
Statistical & Data Analysis Center
Harvard School of Public Health
651 Huntington Ave
Boston, MA 02115-6017
Phone: (617) 432-2426
Fax: (617) 432-2843
Email: shapiro@sdac.harvard.edu

Data Managers
Michael Basar, BA
Frontier Science & Technology Research Foundation
4033 Maple Road
Amherst NY 14226-1056
Phone: (716) 834-0900, Ext. 7271
Fax: (716) 834-8675
Email: basar.michael@fstrf.org

John Gaeddert, MPH
Frontier Science & Technology Research Foundation
4033 Maple Road
Amherst, NY 14226-1056
Phone: (716) 834-0900 Ext. 7477
Fax: (716) 834-8675
Email: gaeddert.john@fstrf.org

Kathleen Kaiser, AAS
Frontier Science & Technology Research Foundation
4033 Maple Road
Amherst, NY 14226-1056
Phone: (716) 834-0900, Ext. 7289
Fax: (716) 834-8675
Email: kaiser.kathleen@fstrf.org

Linda Marillo, B.A.
Frontier Science & Technology Research Foundation
4033 Maple Road
Amherst, NY 14226-1056
Phone: 716-834-0900
E-mail: marillo@fstrf.org

Linda Millar, BS
Frontier Science & Technology Research Foundation
4033 Maple Road
Amherst, NY 14226-1056
Phone: (716) 834-0900, Ext. 7240
Fax: (716) 834-8675
Email: millar.linda@fstrf.org

CEPAC Investigators
Andrea Ciaranello, MD
Massachusetts General Hospital
50 Staniford Street, 9th floor
Boston, MA 02114
Phone: (617) 726-3812
Fax: (617) 726-2691
Email: aciaranello@partners.org

Kenneth Freedberg, MD, MS
Massachusetts General Hospital/Harvard Medical School
50 Staniford Street, 9th floor
Boston, MA 02114-2696
Phone: (617) 724-3341
Fax: (617) 726-2691
Email: kfreedberg@partners.org

Investigators
Risa Hoffman MD, MPH
Division of Infectious Diseases
David Geffen School of Medicine at UCLA
10833 Le Conte Ave 37-121 CHS
Los Angeles, CA 90095
Phone: (310) 623-0617
Fax: (310) 825-3632
Email: rhoffman@mednet.ucla.edu

Anthony Ogwu, MD
Botswana Harvard Partnership
Private Bag BO 320
Gaborone, Botswana
Phone: +267 397 5999
Fax: +267-3901284
Email: aogwu@bhp.org.bw; anthony.ogwu@gmail.com

DAIDS Pharmacist
Lynette Purdue, PharmD
Pharmaceutical Affairs Branch
DAIDS, NIAID, NIH
Room 4107
6700-B Rockledge Drive MSC 7620
Bethesda, MD 20892-7620
Phone: (301) 435-3744
Fax: (301) 402-1506
Email: lpurdue@niaid.nih.gov

Field Representatives
Teri Flynn, RN, MSN, ANP
Massachusetts General Hospital/Harvard ACTU
55 Fruit Street
Boston, MA 02114
Phone: (617) 724-0072
Fax: (617) 643-2899
Email: tflynn@partners.org

Nina K. Sublette, PhD, APRN
The University of Tennessee
Department of Obstetrics and Gynecology
853 Jefferson Avenue
Memphis, TN 38103
Phone: (901)-448-1347
Fax: (901)-595-5068
E-mail: nsublett@utmem.edu

Mary Patricia Toye, RN, MS
Baystate Medical Center and Children's Hospital
759 Chestnut Street
Room C3353
Springfield, MA 01199
Phone: (413) 794-5399
Fax: (413) 794-3207
Email: maripat.toye@bhs.org

Virologist
Susan Fiscus, PhD
Department of Microbiology & Immunology
University of North Carolina School of Medicine
709 Mary Ellen Jones Building
Box# CB# 7290
Chapel Hill, NC 27599-7290
Phone: (919) 966-6872
Fax: (919) 966-9873
Email: fiscussa@med.unc.edu

Immunologist
Adriana Weinberg, MD
University of Colorado Denver
Mail Stop 8604
12700 E. 19th Avenue, Room 11126
Aurora, CO 80045
Phone: (303) 724-4480
Fax: (303) 724-4488
Email: adriana.weinberg@ucdenver.edu

CSS Representative
Morenike Giwa
PO Box 711403
Houston, TX 77271
Phone: (281) 236-6109
Fax: (713) 777-7942
Email: morenikegiwa@yahoo.com

Laboratory Data Coordinators
Amy Jennings, BS
Frontier Science and Technology Research Foundation
4033 Maple Rd
Amherst, NY 14226
Phone: (716) 834-0900, Ext. 7438
Fax: (716) 833-0655
Email: jennings@fstrf.org

Adam Manzella, MA
Frontier Science and Technology Research Foundation
4033 Maple Rd.
Amherst, NY 14226
Phone: (716) 834-0900, Ext. 7418
Fax: (716) 833-0655
Email: manzella@fstrf.org

Amanda Zadzilka, B.S.
Frontier Science and Technology Research Foundation
4033 Maple Rd.
Amherst, NY 14226
Phone: (716) 834-0900, Ext. 7282
Fax: (716) 833-0655
Email: zadzilka@fstrf.org

Laboratory Technologists
William B. Kabat, BS
Special Infectious Diseases Laboratory
The Children's Memorial Hospital
2300 Children's Plaza, Box 82
Chicago, IL 60614-3394
Phone: (312) 227-6290
Fax: (312) 227-9470
Email: bkabat@childrensmemorial.org

Amy James Loftis, BS
IMPAACT Central Laboratory
University of North Carolina at Chapel Hill
116 Manning Drive
Chapel Hill, NC 27599
Phone: (919) 966-6963
Fax: (919) 966-9873
Email: amy_james@med.unc.edu

Protocol Specialist
Anne Coletti, MS
Family Health International (FHI360)
12 Madison Street #2
Medford, MA 02155
Phone: (919) 544-7040 x11238
Fax: (919) 544-0207
Email: acoletti@fhi360.org

Westat Clinical Research Associate
Kathryn Myers, BA, CRA
Clinical Research Associate
Westat
1441 W. Montgomery Ave., WB215
Rockville, MD 20850
Phone: (301) 610-8803
Fax: (301) 279-4545
Email: kathrynmyers@westat.com

Pharmaceutical Industry Representatives
Marisol Martinez
Abbott Laboratories
13192 SW 141 Street
Miami, FL 33186
Phone: (305) 588-1750
Fax: (786) 293-6303
Email: marisol.martinez@abbott.com

Kevin Gray
Associate Director
Virology Collaborative Science Center of Excellence
Bristol-Myers Squibb
777 Scudders Mill Road
Plainsboro, NJ 08536
Phone: (609) 897-5887
Fax: (609) 897-6088
Email: kevin.gray@bms.com

Helene Hardy, PharmD
Associate Director, Virology Medical
Bristol-Myers Squibb
777 Scudders Mill Road
Plainsboro, NJ 08536
Phone: (609) 897-5089
Fax: (609) 897-6068
Email: helene.hardy@bms.com

James F. Rooney, MD
Gilead Sciences, Inc.
333 Lakeside Drive
Foster City, CA 94404
Phone: (650) 522-5708
Fax: (650) 522-5854
Email: jim.rooney@gilead.com

Danielle Porter
Gilead Sciences, Inc.
333 Lakeside Drive
Foster City, CA 94404
Phone: (650) 356-3270
Fax: (650) 522-5595
Email: danielle.porter@gilead.com

Oxana Ivanova
Gilead Sciences, Inc.
333 Lakeside Drive
Foster City, CA 94404
Phone: (650) 372-7656
Fax : (650) 522-5595
Email: Oxana.Ivanova@gilead.com

Wendy Snowden, PhD
Infectious Diseases Medicines Discovery & Development
GlaxoSmithKline Research & Development
1-3 Iron Bridge Road
Stockley Park West, Uxbridge, UB11 1BT
United Kingdom
Phone: (440) 208-9904254
Email: wendy.x.snowden@gsk.com

Helen Watson
Infectious Diseases Medicines Discovery & Development
GlaxoSmithKline Research & Development
1-3 Iron Bridge Road
Stockley Park West, Uxbridge, UB11 1BT
United Kingdom
Phone: (440) 208-9904256
Email: Helen.A.Watson@GSK.com

Randi Leavitt, MD, PhD
Senior Director
Infectious Diseases-Clinical Research
Merck and Company, Inc.
351 N Sumneytown Pike / UG3D-30
North Wales, PA 19454
Phone: (267) 305-7518
Fax : (267) 305-6530
Email: randi_leavitt@merck.com

TABLE OF CONTENTS

STUDY MANAGEMENT viii
GLOSSARY OF TERMS ix
SCHEMA 1

1.0 INTRODUCTION 4

2.0 STUDY OBJECTIVES 17
2.1 Primary Objective and Endpoint 17
2.2 Secondary Objectives 17

3.0 STUDY DESIGN 18

4.0 SELECTION AND ENROLLMENT OF PARTICIPANTS 19
4.1 Inclusion Criteria: Step 1 19
4.2 Exclusion Criteria: Step 1 21
4.3 Inclusion Criteria: Step 2 22
4.4 Exclusion Criteria: Step 2 22
4.5 Inclusion Criteria: Step 3 22
4.6 Exclusion Criteria: Step 3 22
4.7 Enrollment Procedures 22
4.8 Co-enrollment Guidelines 24

5.0 STUDY TREATMENT 24
5.1 Drug Regimens, Administration, and Duration 24
5.2 Drug Administration 26
5.3 Formulations of Study-Supplied Drugs 27
5.4 Drug Supply, Distribution, and Pharmacy 28
5.5 Concomitant Medications Guidelines 28
5.6 Prohibited Medications 28
5.7 Precautionary Medications 28
5.8 Contraception 29

6.0 SUBJECT MANAGEMENT 30
6.1 Timing of Evaluations 30
6.2 Instructions for Evaluations 32
6.3 Management of HAART Regimens 38
6.4 Monitoring and Management of Viral Loads 40
6.5 Management of Antiretroviral Drug Resistance 42
6.6 Management of Participants Who Become Pregnant On Study 42
6.7 Management of Participants Who Develop Active Tuberculosis 43
6.8 Discontinuation of HBV-active ART in HBsAg+ Participants 43
6.9 Toxicity Management 43
6.10 Criteria for Treatment Discontinuation 44
6.11 Criteria for Study Discontinuation 44

**7.0**	**ADVERSE EVENT REPORTING TO DAIDS**	**44**
7.1	Reporting Requirements	45
7.2	Severity Grading	45
7.3	Expedited AE Reporting Period	45
**8.0**	**STATISTICAL CONSIDERATIONS**	**45**
8.1	General Design Issues	45
8.2	Outcome Measures	45
8.3	Randomization and Stratification	46
8.4	Sample Size and Accrual	47
8.5	Monitoring	47
8.6	Analyses	49
**9.0**	**HUMAN SUBJECTS CONSIDERATIONS**	**50**
9.1	Institutional Review Board and Informed Consent	50
9.2	Participant Confidentiality	51
9.3	Study Discontinuation	51
**10.0**	**PUBLICATION OF RESEARCH FINDINGS**	**51**
**11.0**	**BIOHAZARD CONTAINMENT**	**51**
**12.0**	**REFERENCES**	**51**
**APPENDIX I**	**SCHEDULE OF EVALUATIONS**	**59**
**APPENDIX II**	**MATERNAL ENDPOINT DIAGNOSES**	**60**
**APPENDIX III**	**TOXICITY MANAGEMENT**	**63**
**APPENDIX IV**	**SAMPLE INFORMED CONSENT FORM FOR SCREENING AND ENROLLMENT**	**75**
**APPENDIX V**	**SAMPLE INFORMED CONSENT FORM FOR SPECIMEN STORAGE AND FUTURE USE**	**89**
**APPENDIX VI**	**SAMPLE INFORMED CONSENT FORMS FOR WOMEN WHO BECOME PREGNANT WHILE ON STUDY-SUPPLIED STUDY DRUG**	**94**

STUDY MANAGEMENT

For complete guidance on study management questions and communications, please see Section 1 of the IMPAACT 1077HS Manual of Procedures (MOP).

Email the Computer Support Group at the Data Management Center (DMC) (user.support@fstrf.org) to have relevant site personnel added to the protocol email group (promise.prot1077hs@fstrf.org) immediately after completing protocol registration. Inclusion in the protocol e-mail group will ensure that sites receive important information about the study during its implementation and conduct.

General Questions: Email questions concerning any aspect of protocol interpretation and/or study implementation not listed below, including administrative, ethical, regulatory, clinical, counseling, data and laboratory operations, to promise.questions@fstrf.org. See Figure 1-1 in Section 1 of the 1077HS MOP for more information on communication with the PROMISE Questions Email Group.

Clinical Management Questions and Notifications: Email questions concerning clinical management of study subjects and adverse experiences to the study Clinical Management Committee (CMC): promise.cmc1077hs@fstrf.org. Questions related to participant eligibility, potential enrollment of an ineligible participant, and/or deviation from other protocol requirements for screening and enrollment should also be directed to the CMC. See Figures 1-2 and 1-3 in Section 1 of the 1077HS MOP for more information on communications with the CMC. **Do not include the randomized/registered study arm in correspondence with the CMC unless specifically requested or necessary for the clinical management question being asked.**

Co-Enrollment: Email questions related to co-enrollment in 1077HS and other studies to the CMC (promise.cmc1077hs@fstrf.org).

Randomization/Registration: For randomization/registration questions or problems and study identification number (SID) lists, email rando.support@fstrf.org or call the DMC Randomization Desk at (716) 834-0900 x7301.

Computer and Screen Problems: For computer and screen problems, email user.support@fstrf.org or call the DMC at (716) 834-0900 x7302.

Product Package Inserts or Investigator Brochures: Product package inserts or investigator brochures may be accessed on the DAIDS Regulatory Support Center (RSC) web site: http://rsc.tech-res.com.

Study Drug: For questions or problems regarding study drug, dose, supplies, records, and returns, contact the DAIDS Protocol Pharmacist at Lpurdue@niaid.nih.gov or (301) 496-8213.

Study Drug Orders: Email the Clinical Research Products Management Center (BIO.CRPMC.Ph@Thermofisher.com) or call (301) 294-0741.

Expedited Adverse Event (EAE) Reporting/Questions: Contact the DAIDS RSC Safety Office via email (RCCSafetyOffice@tech-res.com) or phone (1-800-537-9979 or +1-301-897-1709) or fax (1-800-275-7619 or +1-301-897-1710). For questions about the DAIDS Adverse Experience Reporting System (DAERS), email DAIDS-ESSupport@niaid.nih.gov. Questions may also be sent from within the DAERS application.

GLOSSARY OF TERMS

3TC	Lamivudine
AE	Adverse event
ALT	Alanine aminotransferase
ANC	Absolute neutrophil count
APR	Antiretroviral pregnancy registry
ART	Anti-retroviral treatment
ARV	Antiretroviral
AST	Aspartate aminotransferase
ATV	Atazanavir
AUC	Area under the curve
BF	Breast-feeding
CDC	Centers for Disease Control and Prevention
CEPAC	Cost-Effectiveness of Preventing AIDS Complications
CLIA	Clinical Laboratory Improvement Act
CMC	Clinical Management Committee
CRF	Case Report Form
DAIDS	Division of AIDS
DBS	Dried Blood Spots
ddI	Didanosine
DHHS	Department of Health and Human Services
DMC	Data Management Center
d4T	Stavudine
DSMB	Data Safety Monitoring Board
DXA	Dual energy X-ray absorpitometry
EIA	Enzyme-linked immunoassay
EAE	Expedited Adverse Event Report
EC	Ethics Committee
EFV	Efavirenz
FDA	Food and Drug Administration
FDC	Fixed dose combination
FF	Formula feeding
FTC	Emtricitabine
GCLP	Good clinical laboratory practice
GL	Guidelines
HAART	Highly Active Antiretroviral Therapy
HBsAb	Hepatitis B surface antibody
HBsAg	Hepatitis B surface antigen
HBV	Hepatitis B virus
ICH	International Conference on Harmonization of Technical Requirements for Registration of Pharmaceuticals for Human Use
IRB	Institutional review board
ITT	Intent to treat
LAR	Legally authorized representative
LFT	Liver function test
LPC	Lab processing chart
LPV	Lopinavir
MOP	Manual of Procedures
MTCT	Mother-to-child HIV transmission
NFV	Nelfinavir
NRTI	Nucleoside reverse transcriptase inhibitor

NNRTI	Non-nucleoside reverse transcriptase inhibitor
NSAIDS	Non-steroidal anti-inflammatory drugs
NVP	Nevirapine
OHRP	Office for Human Research Protections
OI	Opportunistic infection
PBMC	Peripheral blood mononuclear cells
PI	Protease inhibitor
PMTCT	Prevention of mother-to-child transmission
PROMISE	Promoting Maternal and Infant Survival Everywhere
QALY	Quality-adjusted life year saved
QOL	Quality of life
RAL	Raltegravir
RSC	Regulatory Support Center
RPV	Rilpivirine
RTV	Ritonavir
sd	Single dose
SAE	Serious adverse event
SIP	Site implementation plan
SOC	Standard of Care
SOE	Schedule of Evaluations
TB	Tuberculosis
TDF	Tenofovir disoproxil fumarate
TRV	Truvada
ULN	Upper limit of normal
US	United States
VQA	Virology Quality Assurance
VSL	Virology specialty laboratory
WHO	World Health Organization
YLS	Year of life saved
ZDV	Zidovudine

SCHEMA

IMPAACT 1077HS
HAART Standard Version of the PROMISE Study
(DMC Enrollment Screen/CRF Identifier: 1077HS)

DESIGN: A randomized strategy trial conducted among women who received highly active antiretroviral therapy (HAART) during pregnancy for purposes of prevention of mother-to-child transmission (PMTCT) of HIV but do not otherwise meet criteria to initiate HAART for their own health

<u>Step 1</u>

Eligible participants will be randomized within 0-42 days after delivery or other pregnancy outcome to one of two study arms:

Arm A: Continuation of HAART
Arm B: Discontinuation of HAART

<u>Step 2</u>

Participants in Step 1 will be registered to Step 2 if they develop an indication for antiretroviral (ARV) therapy as defined in Section 6.3.2 and otherwise meet Step 2 eligibility criteria per Sections 4.3 and 4.4.

<u>Step 3</u>

Participants in Step 1, Arm A or Step 2 will be registered to Step 3 if they meet therapeutic indications to change HAART as defined in Section 6.3.1 and otherwise meet Step 3 eligibility criteria per Sections 4.5 and 4.6.

SAMPLE SIZE: 2,000 women

POPULATION: HIV-1 infected women who

- Had a documented CD4+ count ≥ 400 cells/mm^3 prior to initiation of HAART for PMTCT during pregnancy and do not currently meet criteria to initiate HAART for their own health,
- Are ARV-naïve except for prior PMTCT, and
- Are willing to be randomized to continue or discontinue HAART after delivery or other pregnancy outcome

STRATIFICATION: By screening CD4+ count 400-499 versus 500-749 versus ≥ 750 cells/mm^3

REGIMEN: The preferred study-supplied HAART regimen is lopinavir/ritonavir (LPV/RTV) plus fixed dose combination tenofovir/emtricitabine (TDF/FTC). Additional ARVs provided for use in this study include fixed dose combination lamivudine/zidovudine (3TC/ZDV), lamivudine (3TC), zidovudine (ZDV), tenofovir (TDF), fixed dose combination tenofovir/emtricitabine/rilpivirine (TDF/FTC/RPV), didanosine (ddI), atazanavir (ATV), raltegravir (RAL), and ritonavir (RTV).

While LPV/RTV plus TDF/FTC is the preferred study-supplied regimen, the study clinicians in conjunction with participants should determine the optimal drug combination for each participant. Likewise, secondary and subsequent regimens are not defined by this protocol and should be determined at the discretion of the study clinicians. ARVs not provided by the study may be used if the regimen meets the study definition of HAART, i.e., three or more agents from two or more classes of antiretroviral therapy, and the ARVs are provided by prescription.

DURATION: Women will be followed until 84 weeks after the last woman is randomized.

OBJECTIVES: Primary Objective and Endpoint

To determine whether continuation of HAART (Arm A) after delivery or other pregnancy outcome reduces morbidity and mortality compared to discontinuation and re-initiation of HAART according to current standards of care (Arm B). The primary combined endpoint includes death, AIDS-defining illness, and serious non-AIDS-defining cardiovascular, renal, and hepatic events.

Secondary Objectives

1. To determine, characterize, and compare the rates of AIDS-defining and HIV-related illnesses, opportunistic infections, immune reconstitution inflammatory syndromes, and other targeted medical conditions (see Appendix II), with regard to outcomes and survival.
2. To assess toxicity, both selected moderate (Grade 2) laboratory abnormalities (renal, hepatic, and hematologic) and severe (Grade 3 or Grade 4) laboratory values and signs and symptoms.
3. To compare emergence of HIV-1 resistance to ART during the 1st, 2nd, and 3rd years following randomization and at end of study.
4. To evaluate rates of self-reported adherence to HAART and its association with the primary endpoint and with CD4+ cell count, HIV-1 viral load, and HIV-1 resistance patterns at 1, 2, and 3 years following randomization.
5. To compare quality of life measurements between the study arms at 1, 2, and 3 years following randomization.
6. To investigate changes in plasma concentrations of inflammatory and thrombogenic markers between arms and to correlate these markers to clinical events.
7. To evaluate cost effectiveness and feasibility of the trial strategies.

STUDY SITES: IMPAACT sites and other NIAID- and NICHD-funded sites in the US, Brazil and other countries that meet study-specific participation criteria established by the IMPAACT leadership. The complete listing of participating sites is available on the Protocol Specific Web Page. Transfer from an enrolling IMPAACT site to an adjacent ACTG site for long term follow-up may be approved as part of the Site Implementation Plan and is encouraged at IMPAACT sites co-located at the same institution as an ACTG site.

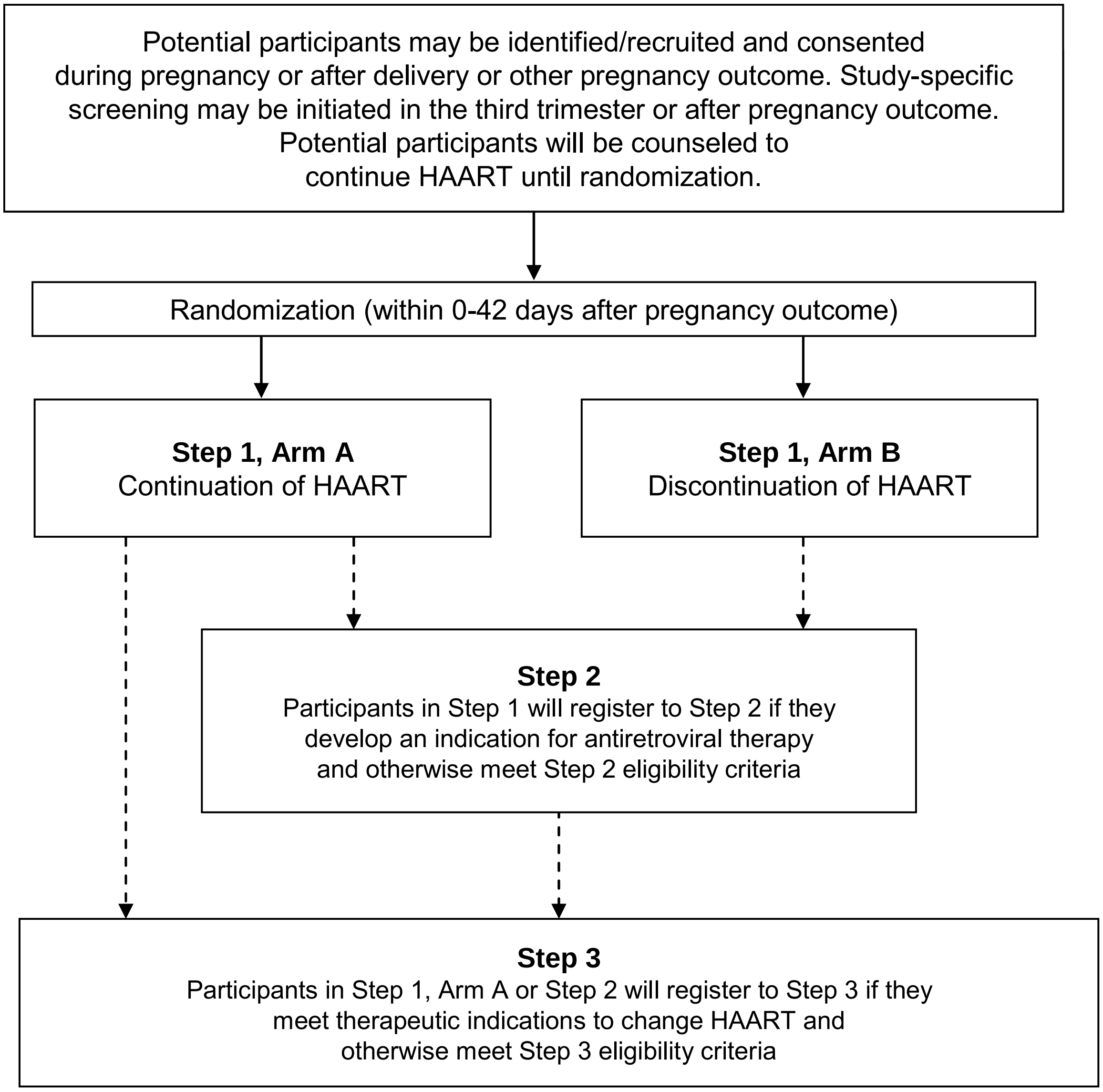

1.0 INTRODUCTION

Over the past two decades, considerable strides have been made in the prevention of mother-to-child transmission (PMTCT) of HIV. The administration during pregnancy of virologically suppressive antiretroviral drug combinations conventionally referred to as highly-active anti retroviral therapies (HAART), cesarean section delivery, and infant formula-feeding (FF) have led to a decrease of the risk of MTCT to less than 2% and the virtual elimination of new pediatric HIV infection in the U.S., Europe, and other resource-advantaged settings. Remaining issues essentially revolve around improved service delivery, in particular the detection of all HIV-infected women prior to conception and early enough during pregnancy so that appropriate counseling and appropriate ART can be provided. Determining the safety of stopping HAART when used solely for prophylaxis of MTCT remains an important unresolved issue.

Risks and Benefits of HAART after Pregnancy

The main question to be addressed by 1077HS is the risk and benefits of stopping HAART after completion of pregnancy for women who received this strategy solely to prevent MTCT. In settings where HAART is routinely used for PMTCT, HAART is typically discontinued after delivery in women who had CD4+ cell counts > 350 cells/mm^3 at initiation of HAART for PMTCT, although the safety of this approach has not been evaluated. A December 2009 revision of US treatment guidelines for adults and adolescents increased the recommended threshold for treatment of men and non-pregnant women from 350 to 500 cells/mm^3 based on observational data showing increased morbidity and mortality when HAART was delayed to the 350 cell/mm^3 threshold. More recently, a March 2012 update of US guidelines strengthened the recommendation to initiate HAART at CD4+ cell counts between 350 and 500 cells/mm^3, based on increasing evidence from both non-randomized studies and a randomized clinical trial. This update also included a moderate recommendation to initiate HAART at CD4+ cell counts above 500 cells/mm^3, based on expert opinion in the absence of data from randomized or non-randomized studies. This update noted the benefits of HAART for preventing secondary transmission of HIV but acknowledged that randomized data to support recommendations to initiate HAART at CD4+ cell counts above 500 cells/mm^3 are lacking. In addition, it remains unclear whether the data on which the recommendations were based can be generalized to postpartum women.

Limited data exist on long-term effects of stopping HAART after pregnancy. While data comparing women stopping ZDV at delivery to untreated women in PACTG 076 and women stopping or continuing ZDV monotherapy at delivery in PACTG 185 did not suggest harm from short-term ZDV use for PMTCT (1;2), recent data from studies comparing scheduled treatment interruptions to continuous therapy in non-pregnant adults have raised concerns that stopping HAART regimens may be detrimental in terms of clinical outcomes when compared to continued therapy. Small studies have not suggested harm from scheduled treatment interruptions, although all have shown lower CD4+ cell counts at the end of the study in treatment interruption groups (3-5). The CD4-guided therapy arm of the Trivacan trial in Africa was stopped early because of a significantly increased rate of serious morbidity in the interruption arm (15.2/100 person years) compared to the continuous therapy arm (6.7/100 person-years, RR 2.27, 95% CI 1.15-4.76) (6). At enrollment, all participants had CD4+ cell counts > 350 cells/mm^3 and HIV-1 RNA < 300 copies/mL. Therapy was reinstituted for a CD4+ count < 250 cells/mm^3. The largest trial reported to date, the SMART study, used similar inclusion and therapy interruption/reinstitution guidelines and included 5,472 participants (7). In

SMART, the rate of opportunistic disease or death was 3.3/100 person years in the therapy interruption group and 1.3/100 person years in the continuous therapy group (HR 2.6, 95% CI 1.9-3.7 for interruption compared to continuous group). In a subset of SMART participants who were either ART naïve at enrollment or off therapy for several months, a population similar to pregnant women likely to be initiated on HAART during pregnancy, similar inferior clinical outcomes were noted among those who stopped HAART in terms of clinical outcomes (7). Of note, the hazard ratio for major cardiovascular, renal, and hepatic disease was 1.7 (95% CI 1.1-2.5) for the group randomized to stop HAART compared to the continuous group, despite less overall ARV drug exposure in the interruption group, an unexpected result. These findings suggest that among patients with CD4+ cell counts > 350 cells/mm^3, being on HAART is associated with an increase in time to HIV disease progression compared to those who stop HAART. Updated results from the long term follow-up of the SMART study suggest that re-initiation of therapy after the interruption was associated with a delay in CD4+ T cell response with failure of mean CD4+ cell count to reach the baseline value in the interruption arm by end of the study (8). Other key findings from SMART suggest that interruption of HAART is associated with surprisingly rapid changes in inflammatory and coagulation markers, factors that may influence the risk of organ damage.

Scheduled treatment interruption studies vary widely in inclusion criteria, interruption schedules, and threshold for restarting therapy, thus making comparisons between studies and extrapolation to women receiving ARVs for PMTCT difficult. In addition, the risk versus benefit considerations for initiation of HAART with a CD4+ cell count over 350 cells/mm^3 with continuation indefinitely are unclear. The short-term risk of AIDS and death at CD4+ cell counts > 350 cells/mm^3 is low, and the potential absolute risk reductions associated with treatment in such patients are therefore small. Within the ART Cohort Collaboration, the absolute 3-year risk differences between those with counts 200 to 349 cells/mm^3 and those with CD4+ cell counts ≥ 350 cells/mm^3 were only 1.3% (for those with HIV-RNA < 100,000 copies/mL) and 1.7% (for those with HIV-RNA ≥ 100,000 copies/mL)(9). These differences were similar through 5 years of observation (10).

Data from the AIDS Therapy Evaluation Project, Netherlands (ATHENA), have demonstrated that patients who start therapy with CD4+ cell counts > 350 cells/mm^3 were significantly more likely to achieve CD4+ cell counts > 800 cells/mm^3 after seven years of HAART than those who initiated therapy at lower CD4+ cell counts (11). A long-term study based on the Johns Hopkins Clinical Cohort demonstrated that patients who initiated HAART with a CD4+ cell count < 350 cells/mm^3 were significantly less likely to achieve a CD4+ cell count > 500 cells/mm^3 after six years of HAART compared to those who started therapy at higher CD4+ cell counts (12).

Some of the non-AIDS defining complications of HIV infection, such as herpes zoster, idiopathic thrombocytopenic purpura, and neoplastic disorders constitute severe conditions that may affect the quality of life and add medical expenses. In the pre-HAART era, the incidence of herpes zoster was 3 to 5% per year in HIV-infected patients (13-15), which is 10 to 20 times higher the incidence of herpes zoster in the young adult population (16;17). Furthermore, HIV-infected individuals can also develop complications such as post-herpetic neuralgia, chronic skin lesions, retinitis, and encephalitis (18-20). Herpes zoster requires antiviral therapy in HIV-infected patients, added by other medical interventions when complications occur. These complications may be decreased by continuing HAART after delivery and will be evaluated in the 2 treatment arms of this study. In addition, non-AIDS defining malignancies associated

with opportunistic viral infections carry high morbidity and mortality and require expensive medical interventions. In this study, we will compare the incidence of neoplastic disorders (both AIDS-defining and non-AIDS-defining), between the two arms. Other malignant disorders such as breast, lung, or colon cancer, however, may not be affected by continuous HAART.

Factors that might support initiating therapy as early as possible include the potential negative impact of uncontrolled HIV replication on renal, hepatic, neurologic, cognitive and immunological functions (21). Earlier treatment of HIV infection may also have positive public health implications, as it may reduce HIV transmission (22). This may have significant implications among individuals in discordant relationships (i.e., HIV infected individuals with HIV-uninfected sexual partners) as was demonstrated in the HPTN 052 study (22A, 22B) and recognized by the World Health Organization in its recent guidance on couples HIV testing and counselling including antiretroviral therapy for treatment and prevention in serodiscordant couples (22C). HPTN 052 also demonstrated a lower rate of clinical illness when treatment was initiated at CD4+ cell counts between 350 and 550 cells/mm^3, compared to CD4+ cell counts below 250 cells/mm^3; however there was no difference observed in mortality in this study.

Despite possible benefits of treatment of persons with CD4+ cell counts > 350 cells/mm^3, there are also considerations that argue against earlier therapy. First, the potential relative reduction in risk of non-AIDS events/morbidity with ART as a result of CD4+ cell count increase and viral load suppression may not be large. Second, although there are now several reasonably safe and well-tolerated options for first-line regimens, the long-term toxicities remain unknown. Third, ART requires life-long adherence to therapy. Some patients may find that the need to take daily medications decreases quality of life, even without side effects, and lastly, non-adherence may increase the development of resistance, limiting future treatment options. Adherence in the early postpartum period is of special concern.

As the use of HAART during pregnancy for PMTCT continues to increase worldwide, the risks and benefits of continuing versus stopping therapy in reproductive age women with CD4+ cell counts > 350 cells/mm^3 must be evaluated. A critical issue in the management of HIV infection among women is to determine how interventions to reduce perinatal transmission impact maternal health in the short- and long-term. If women who receive HAART for PMTCT incur some penalty in terms of their own health, then this may offset any benefits of a maternal HAART strategy for PMTCT. Furthermore, if continuing HAART at the conclusion of the PMTCT among women with CD4+ cell counts > 350 cells/mm^3 is associated with reduced morbidity, these data will add to the growing body of evidence suggesting that earlier HAART may be associated with benefits. On the other hand, if HAART adherence patterns after delivery are sub-optimal with an increased risk of development of resistance to antiretrovirals or increased toxicities due to other co-morbidities, then the benefits of treatment continuation once started for this population would need careful reassessment. The design of the PROMISE study provides a unique opportunity to address this crucial question regarding optimal use of ARV therapy among childbearing HIV-infected women. A substudy to be conducted on stored specimens will evaluate the emergence of drug resistance among women participating in the study; the substudy is described briefly below.

Cost-Effectiveness Analysis

In addition to the critical clinical questions raised by continued versus interrupted HAART postpartum, an additional domain that can be addressed in 1077HS is the cost-effectiveness of the different therapeutic strategies. With the advent of successful ARV therapy, the cost of ARVs has emerged as a primary concern in both resource-limited and resource-advantaged settings. The PROMISE study will allow a detailed assessment of not only the cost but also the cost-effectiveness of these interventions, providing the needed data to inform policy decisions across a wide spectrum of health care delivery systems. To understand these policy implications, the team has added internationally recognized expertise in HIV cost-effectiveness analysis using the Cost-Effectiveness of Preventing AIDS Complications (CEPAC) model and team.

The CEPAC model is a widely published HIV simulation model, which incorporates data on natural history, treatment efficacy, cost, and quality of life, in order to project long-term outcomes and policy relevance from shorter-term clinical trial data (23-34). The team will conduct simulations to understand the cost-effectiveness of the trial strategy. The CEPAC analyses will report outcomes limited to the timeframe of the trial, as well as projected beyond the end of the trial (35;36), including opportunistic infections (OIs), mortality, total direct medical costs, and cost-effectiveness (dollars per year of life saved ($/YLS) and dollars per quality-adjusted life year saved ($/QALY)). These analyses will be presented in conjunction with the main trial reports. The CEPAC team has extensive experience producing policy-relevant analyses, which in many cases have led directly to changes in HIV guidelines for care (37-41).

ARV Resistance Investigations

As use of antiretroviral drugs (ARVs) for HIV-1 prevention and treatment increases globally, resistance to ARVs will likely become more common. Emergence of drug resistance may be related to several factors including: use of regimens that are not fully suppressive, poor adherence to ARV regimens (because of interruption in the availability of ARVs, toxicities, co-morbidities, and/or non-compliance), and the low HIV-1 genetic threshold for resistance to some drugs. Host genetic factors may also affect bioavailability of ARVs, influencing emergence of resistance in some settings. The PROMISE study provides a number of opportunities to explore the development of resistance in women exposed to different ARV regimens.

The PROMISE study will be conducted at sites worldwide (with different versions to be implemented in different settings depending on the local standard of care for prevention of mother-to-child HIV transmission); therefore, women infected with a variety of HIV-1 subtypes will be enrolled. Previous studies show that HIV-1 subtype can dramatically affect the emergence and persistence of ARV resistance in women and infants in the setting of PMTCT. PROMISE will be the first study to compare HIV transmission and the development/duration of ARV resistance in women infected with a large variety of HIV-1 subtypes.

Evaluations to be conducted include HIV-1 resistance testing, population sequencing, minority variants analysis and HIV-1 subtype determination. Other related studies may include characterization of HIV viruses from women (e.g., sequencing of regions other than pol, and assays measuring phenotypic resistance, replication capacity, and HIV tropism), and to evaluate the host response to HIV infection. These analyses may involve comparisons between groups, tests of association between resistance status

and clinical outcomes or analysis of descriptive information concerning various aspects of resistance. Because we will not know in advance which specimens will eventually be selected for resistance testing, specimens will be stored for resistance testing at selected study visits.

Rationale for Collecting Samples for Inflammatory Markers

Studies have shown that HIV replication increases postpartum, even in patients who are adherent to HAART (42-44). The etiology of this increased replication is unclear, but may be related to hormonal changes, adherence, viral resistance, or other physiologic changes leading to immune activation. Preliminary research has demonstrated that HIV-1-infected pregnant women have higher levels of the inflammatory markers neopterin and ß2-microglobulin, and these markers may remain elevated up to 3 to 6 months postpartum (45;46). Additionally, the coagulation marker D-dimer rises in the third trimester of pregnancy and continues to rise further, peaking at approximately 10 days postpartum, regardless of mode of delivery indicating that the postpartum period may represent a state of further heightened immune activation (47). Most studies of inflammatory and coagulation biomarkers in HIV have been undertaken in men and non-pregnant women, with the largest of these studies showing an association between elevated baseline concentrations of highly sensitive C-reactive protein, IL-6, and D-dimer, and all cause mortality (48). We propose to characterize inflammatory and coagulation biomarkers in pregnant and postpartum women with HIV-infection, and to compare elevations in these biomarkers in women who stop HAART after delivery versus those who continue HAART postpartum. We hypothesize that stopping HAART postpartum will be associated with a rise on these inflammatory and thrombogenic markers and that these markers will correlate with subsequent clinical events.

Rationale for Collecting Peripheral Blood Mononuclear Cells (PBMCs) for Immunologic Studies

This study offers a unique opportunity to increase our understanding of the immunopathogenesis of AIDS and non-AIDS complications of HIV infections as they relate to pregnancy. One of several potential factors contributing to the profound immunosuppression of HIV-infected individuals is excessive immune regulation. In patients infected with HIV, persistent antigenic stimulation may contribute to the expansion of regulatory T cells (T_R), which in turn may decrease the host's ability to contain viral replication (49-52). Naturally occurring human CD4+ T_R have been extensively characterized (53). These cells express FoxP3 in abundance, which appears to have an important role in activating the regulatory program and is used to discriminate T_R from other CD4+ cells with otherwise similar phenotypes (54-57). Other cell surface markers that have been used to identify T_R are CD25, CTLA4, GITR, GPR83, PD-1, and CD127.T_R that do not express CD25, characteristically secrete TGFβ or IL10 (58-63). Less is known about T_R invoked during the adaptive immune response or CD8+ T_R. Studies indicate that CD8+ T_R also contribute to deactivation of the immune response and tolerance (64-68). Th17 cells, characterized by the production of IL17, are deemed to mediate auto-inflammatory diseases and other disorders associated with exacerbated inflammatory reactions (69). They share a progenitor with the T_R (70). Most authors agree that the genesis of both T_R and Th17 depends on TGFβ-mediated signals. In the presence of excess IL6, IL21, IL 23, and/or IL1β, Th17 cells are generated, whereas in the absence of IL6 and other pro-inflammatory cytokines the cell program is biased towards the development of T_R. (71-73) Pregnancy is associated with expansion of T_R, which play an important role in the tolerogenic effect of pregnancy. Several T_R

phenotypes have been described in association with pregnancy, excess estrogen or progesterone production, including C4+CD25+FoxP3+, CD4+CTLA4+, CD8+CTLA+, CD4+PD-1+, and CD8+IL10+ cells (74-78). Hence, HIV-infected pregnant women have multiple factors that may contribute to the expansion of both the T_R and the Th17 populations. Both excessive T_R and Th17 may have deleterious effects on the general health of the host: the first by decreasing immune defenses against infections and neoplasias and the second by increasing the risk of inflammatory complications. It is hypothesized that HIV-infected women who remain on HAART after pregnancy have lower frequencies of T_R and Th17 cells.

Rationale for Selection of CD4+ Cell Counts for Study Entry and Re-Initiation of HAART

A CD4+ cell count at or above 400 cells/mm^3 was chosen for inclusion in this study and a CD4+ cell count below 350 cells/mm^3 was chosen as the threshold for re-initiation of HAART in this study. The rationale for these selections is provided below.

CD4+ Cell Threshold for Study Entry

CD4+ cell counts are expected to rise during pregnancy in response to HAART and to increase further in the postpartum period as a result of reversal of hemodilution of pregnancy. CD4+ cell counts also are expected to remain elevated for a prolonged period after HAART, based on recent data from the DREAM cohort in which CD4+ cell counts remained above baseline for more than one year after discontinuation of HAART for PMTCT in women with CD4+ counts > 350 cells/mm^3 (79). Similar data on durable (up to three years) CD4+ cell response after HAART for PMTCT have been reported from Brazil (79A). Additionally, in unpublished data from the IMPAACT P1025 study, among women who have a pre-antiretroviral CD4+ cell count above 400 and are treatment-naïve as defined in IMPAACT 1077HS, the pre-antiretroviral CD4+ cell count was at least 500 cells/mm^3 for 68% of women and the CD4+ cell count at or near delivery was at least 500 cells/mm^3 for 87% of women. Based on these data, most women enrolled in IMPAACT 1077HS are expected to have postpartum CD4+ cell counts above 500 cells/mm^3 at the time of randomization and, for such women, there are no data from randomized trials indicating the risk-benefit profile associated with treatment deferral. PROMISE will provide randomized data to answer this important question and to better characterize the risks and benefits of treatment for women during this period of time.

CD4+ Cell Threshold for Re-Initiation of HAART

A CD4+ cell count below 350 cells/mm^3 was chosen as the threshold for re-initiation of HAART in this study. This is consistent with current WHO guidelines for treatment of HIV-infected adults and adolescents. It is acknowledged, however, that current US treatment guidelines recommend initiating HAART at higher CD4 cell counts, such that women started on a HAART regimen during pregnancy could continue HAART postpartum (lifelong), regardless of CD4 count. While evidence in support of this approach is accumulating, it is not based on data from randomized trials, and the observational studies that support this approach have included more men than women, as well as older patient populations; as such, their results may not be generalizable to younger pregnant and postpartum women — the population of interest for PROMISE. While randomized data from HPTN 052 demonstrated a clinical benefit for patients who started HAART at CD4+ cell counts between 350 and 500 (versus below 250), half of the observed clinical events occurred among patients with CD4 cell counts below 350

and the difference between arms was driven solely by extrapulmonary TB. Additionally, age was a strong predictor of risk and the median duration of follow-up was only 1.7 years, limiting the ability to fully evaluate longer term outcomes. Thus, the risks and benefits of continuing HAART among otherwise healthy women of reproductive age have not yet been clearly established. The selection of 350 as the CD4+ cell count threshold for re-initiation of HAART reflects the objective of this study to provide randomized data to characterize the risk-benefit profile in this population. It is important for study participants in the US to acknowledge that the option to remain on HAART after delivery is available outside of the study.

Study Drugs

While this is a strategy trial rather than an evaluation of specific drug regimens, selected drugs will be available through the study to assure access to potent drugs for all women. Summary information for drugs in the first-line regimen is provided here.

Lopinavir/Ritonavir (ABT-378/Ritonavir; LPV/RTV; Kaletra®, Aluvia®)

Lopinavir (LPV, ABT-378) is a potent inhibitor of HIV-1 protease. When co-formulated with LPV, ritonavir (RTV) inhibits the CYP3A metabolism of LPV, thereby providing increased plasma levels of LPV. Lopinavir/ritonavir (LPV/RTV) in a single fixed-dose combination capsule (Kaletra) was evaluated and approved by the US FDA in 2000 for use in combination with other ARVs for the treatment of HIV-1 infection. A tablet formulation of LPV/RTV received US FDA approval in October 2005.

Clinical Experience

A phase III study (M98-863) evaluated the safety and efficacy of LPV/RTV plus d4T and 3TC versus nelfinavir (NFV) plus stavudine d4T and 3TC (80) in treatment naïve patients. The primary efficacy analyses included the proportion of participants with HIV-1 RNA level < 400 copies/mL at week 24 and the duration of virologic response through week 48. Overall, 326 participants were assigned to the LPV/RTV group and 327 to the NFV group. Baseline HIV-1 RNA level was 4.9 $\log_{10}$ for each group. Baseline CD4+ cell counts were approximately 260 cells/mm^3 for each group. At 48 weeks, the proportion of participants with HIV-1 RNA levels < 400 (< 50) copies/mL by intention to treat (ITT) (missing value = failure, M = F) analysis were 75% (67%) for the LPV/RTV group compared with 63% (52%) for the NFV group (p< 0.001) [Proportion < 400 (< 50) copies/mL on treatment was 93% (83%) versus 82% (68%)]. Mean changes in CD4+ cell counts were 207 cells/mm^3 for the LPV/RTV group and 195 cells/mm^3 for the NFV group. Overall, 2% of participants in the LPV/RTV group and 4% in the NFV group discontinued therapy at or before week 48 because of study drug-related adverse events (AEs). No significant differences between the two treatment groups were noted in AEs, except for increases in triglycerides. Moderate and severe AEs for the LPV/RTV and NFV groups, respectively, were as follows: diarrhea (16% and 17%), nausea (7% and 5%), asthenia (4% and 3%), abdominal pain (4% and 3%), SGPT/ALT > 5x upper limit of normal (ULN) (4% each), total cholesterol > 300 mg/dL (9% and 5%), triglycerides > 750 mg/dL (9% and 1%, p < 0.001), and amylase > 2 x ULN (3% and 2%).

Durability of response has been demonstrated with LPV/RTV in ARV-naive patients in the above study with 79% of the 326 participants on the LPV/RTV arm maintaining virologic suppression (viral load of < 400 copies/mL) at 96 weeks, compared with 58% on the NFV arm (81).

LPV/RTV has been studied in combination with tenofovir disoproxil fumarate (TDF) and emtricitabine (FTC). Study 418 is a randomized, open-label, multicenter trial comparing treatment with LPV/RTV 800/200 mg once-daily plus TDF and FTC versus LPV/RTV 400/100 mg twice-daily plus TDF and FTC in 190 ARV-naïve patients. Patients had a mean age of 39 years (range: 19-75), 54% were Caucasian, and 78% were male. Mean baseline CD4+ cell count was 260 cells/mm^3 and mean baseline plasma HIV-1 RNA was 4.8 log copies/mL. Through 48 weeks of therapy, 71% in the LPV/RTV once-daily arm and 65% in the LPV/RTV twice-daily arm achieved and maintained HIV-1 RNA < 50 copies/mL (95% confidence interval for the difference, -7.6%-19.5%). Mean CD4+ cell count increases at Week 48 were 185 cells/mm^3 for the LPV/RTV once-daily arm and 196 cells/mm^3 for the LPV/RTV twice-daily arm.

Safety Profile

LPV/RTV has been studied in patients as combination therapy in phase I/II and phase III trials. The most common AEs associated with LPV/RTV therapy were diarrhea and nausea, which were generally of mild-to-moderate severity. Rates of discontinuation of randomized therapy due to AEs were 5.8% in LPV/RTV-treated and 4.9% in NFV-treated patients in study M98-863. Pancreatitis has been reported in patients receiving LPV/RTV, although a causal relationship has not been established. The most common laboratory abnormalities in patients receiving LPV/RTV were elevations in triglycerides and cholesterol, which may be marked, and less commonly elevations in AST and ALT.

There has been no evidence of teratogenicity with administration of LPV/RTV to pregnant rats or rabbits. In rats treated with maternally toxic dosage (100 mg LPV/50 mg RTV/kg/day), embryonic and fetal developmental toxicities (early resorption, decreased fetal viability, decreased fetal body weight, increased incidence of skeletal variations and skeletal ossification delays) were observed. In rabbits, no embryonic or fetal developmental toxicities were observed with maternally toxic dosage, where drug exposure was 0.6-fold for LPV and 1.0-fold for RTV of the exposures in humans at recommended therapeutic dose. In the Antiretroviral Pregnancy Registry (APR), sufficient numbers of first trimester exposures to LPV/RTV have been monitored to be able to detect at least a 2-fold increase in risk of overall birth defects. No such increase in birth defects has been observed with LPV/RTV. The prevalence of birth defects with first trimester LPV + RTV exposure was 1.8% (95% CI: 0.7% -3.9%) compared with total prevalence of birth defects in the US population based on CDC surveillance of 2.7% (82). 79% of these women evaluated postpartum had area under the curve (AUC) values above the percentile. As with ritonavir, placental passage of lopinavir was limited.

A recent labeling update approved by the US FDA described the effects of LPV/RTV on electrocardiogram. QTcF interval was evaluated in a randomized, placebo and active (moxifloxacin 400 mg once-daily) controlled crossover study in 39 healthy adults (M06-809), with 10 measurements over 12 hours on Day 3. The maximum mean time-matched (95% upper confidence bound) differences in QTcF interval from placebo after baseline-correction were 5.3 (8.1) and 15.2 (18.0) mseconds (msec) for 400/100 mg twice-daily and supratherapeutic 800/200 mg twice-daily lopinavir/ritonavir, respectively. Lopinavir/ritonavir 800/200 mg twice daily resulted in a Day 3 mean Cmax approximately 2-fold higher than the mean Cmax observed with the approved once daily and twice daily lopinavir/ritonavir doses at steady state.

PR interval prolongation was also noted in subjects receiving lopinavir/ritonavir in the same study on Day3. The maximum mean (95% upper confidence bound) difference from placebo in the PR interval after baseline-correction were 24.9 (21.5, 28.3) and 31.9 (28.5, 35.3) msec for 400/100 mg twice-daily and supratherapeutic 800/200 mg twice-daily lopinavir/ritonavir, respectively.

Additional information can be found in the most recent Kaletra® package insert, which states that LPV/RTV prolongs the PR interval in some patients and should be used with caution in those who have pre-existing structural heart disease, conduction system abnormalities, or other cardiac diseases.

<u>Emtricitabine (FTC, Emtriva®)</u>

Emtricitabine (FTC) (5-fluoro-1-(2R,5S)-[2-(hydroxymethyl)-1,3-oxathiolan-5-yl] cytosine) is a synthetic nucleoside analogue with activity against HIV-1 reverse transcriptase. FTC is the negative (-) enantiomer of a thio analogue of cytidine, which differs from cytidine analogues in that it has a fluorine in the 5-position. FTC is phosphorylated by cellular enzymes to form the active intracellular metabolite, emtricitabine 5'-triphosphate (FTC-TP), which is a competitive inhibitor of HIV-1 RT and terminates the growing DNA chain.

<u>Clinical Experience</u>

Two phase III controlled studies (FTC-301A and FTC-303) provide the most information concerning the safety and efficacy of FTC in HIV-1-infected adults treated for extended periods with combinations of ART (83).

Study FTC-301A was a 48-week, double-blind, active-controlled, multicenter study comparing FTC (200 mg) QD to d4T (stavudine) in combination with QD open-label didanosine (ddI) and EFV in 571 ARV-naïve patients with plasma HIV-1 RNA 5,000 copies/mL. Patients had a mean age of 36 years (range: 18-69), 85% were male, 52% Caucasian, 16% African American and 26% Hispanic. Patients had a mean baseline CD4+ cell count of 318 cells/mm^3 (range: 5-1317) and median baseline plasma HIV-1 RNA of 4.9 log_{10} copies/mL (range: 2.6-7.0). Thirty-eight percent of patients had baseline viral loads > 100,000 copies/mL and 31% had CD4+ cell counts <200 cells/mL.

At week 48, FTC was statistically superior to d4T with 81% of the patients in the FTC treatment group achieving and maintaining plasma HIV-1 RNA < 400 copies/mL compared with 68% of the patients in the d4T treatment group. Likewise, the proportion of patients who achieved and maintained plasma HIV-1 RNA < 50 copies/mL was statistically significantly different with 78% of patients in the FTC treatment group compared with 59% of patients in the d4T treatment group. Additionally, FTC-treated patients had a statistically greater increase in CD4+ cell count at week 48 with a mean increase from baseline of 168 cells/mm^3 for the FTC group and 134 cells/mm^3 for the d4T group. The proportion of patients with virologic failure was 3% in the FTC group and 11% in the d4T group. A statistically greater proportion of patients in the d4T group experienced an adverse event that led to study drug discontinuation through Week 48 than in the FTC group (13% versus 7%).

Study FTC-303 was a 48-week, open-label, active-controlled, multicenter study comparing FTC to 3TC in combination with d4T or ZDV and a protease inhibitor (PI) or NNRTI in 440 patients who were on a 3TC-containing triple-ARV regimen for at least 12 weeks prior to study entry and had plasma HIV-1 RNA ≤ 400 copies/mL.

Patients were randomized 1:2 to continue therapy with 3TC (150 mg BID) or to switch to FTC (200 mg QD). All patients were maintained on their stable background regimen. Patients had a mean age of 42 years (range: 22-80), 86% were male, 64% Caucasian, 21% African American, and 13% Hispanic. Patients had a mean baseline CD4+ cell count of 527 cells/mm^3 (range: 37-1909) and median baseline plasma HIV-1 RNA of 1.7 $\log_{10}$ copies/mL (range: 1.7-4.0). The median duration of prior ART was 27.6 months.

Through 48 weeks of therapy, there was no statistically significant difference between treatment groups in efficacy outcomes. The proportion of patients with virologic failure was 7% in the FTC arm and 8% in the 3TC arm. Through 48 weeks of therapy, the proportion of patients who achieved and maintained plasma HIV-1 RNA < 400 copies/mL was 77% in the FTC arm and 82% in the 3TC arm. The difference was largely attributed to attrition from the study and not loss of virological activity. Likewise, the proportion of patients who achieved and maintained plasma HIV-1 RNA < 50 copies/mL was 67% in the FTC arm and 72% in the 3TC arm. The mean increase from baseline in CD4+ cell counts was 29 cells/mm^3 in the FTC arm and 61 cells/mm^3 in the 3TC arm. These findings support equivalent efficacy of FTC 200 mg once-daily and 3TC 150 mg administered twice daily.

Safety Profile

More than 2,000 adult patients with HIV infection have been treated with FTC alone or in combination with other ARVs for periods of 10 days to 200 weeks in phase I-III clinical trials. Assessment of adverse events is based on data from studies FTC-301A and FTC-303 in which 571 treatment naïve (FTC-301A) and 440 treatment experienced (FTC-303) patients received FTC 200 mg (n = 580) or comparator drug (n = 431) for 48 weeks.

The most common AEs that occurred in patients receiving FTC with other ARVs in clinical trials were headache, diarrhea, nausea, and rash event, which were generally mild to moderate severity. Approximately 1% of patients discontinued participation in the clinical studies due to these events. All AEs were reported with similar frequency in FTC and control treatment groups with the exception of skin discoloration, which was reported with higher frequency in the FTC-treated group. Skin discoloration, manifested by hyperpigmentation on the palms and/or soles was generally mild and asymptomatic.

Laboratory abnormalities in these studies occurred with similar frequency in the FTC and comparator groups.

Embryofetal toxicology studies of FTC in mice and rabbits have not shown increased incidence of malformations (84). FTC is FDA category B. There was no evidence of impaired fertility or fetal harm in studies of mice at 60 times the human exposure and in rabbits at 120 times the human exposure. Defects have been noted in 6/194 first trimester exposures and 1/103 later exposures in pregnancy reported to the APR. The APR does not compute rates of confidence intervals until at least 200 exposures in a given time period have been reported.

Tenofovir Disoproxil Fumarate (TDF, Viread®)

Tenofovir disoproxil fumarate (TDF), (9-[(R)-2-[[bis[[(isopropoxycarbonyl)oxy] methoxy]phosphinyl]methoxy]propyl] adenine fumarate (1:1)) (formerly known as PMPA prodrug or GS-4331-05) is approved for the treatment of HIV-1 infection and hepatitis B virus infection. TDF is an orally bioavailable prodrug of tenofovir (TVF), an acyclic nucleotide analogue with activity in vitro against retroviruses, including HIV-1 and HIV-2, and against hepadnaviruses. TDF is absorbed in the gut and rapidly converted to tenofovir, which circulates in the bloodstream and is taken up intracellularly. There it is metabolized to the active metabolite, tenofovir diphosphate (PMPApp), which is a competitive inhibitor of HIV-1 reverse transcriptase that terminates the growing DNA chain. Although TDF is a nucleotide analogue, it has the same mechanism of action and resistance pattern as NRTIs. Therefore, for simplification of discussion, TDF will be referred to as an NRTI in this study.

Clinical Experience

Gilead Study 903 was a 144-week randomized, double-blind trial designed to compare the efficacy and safety of a treatment regimen of TDF, 3TC, and EFV to a regimen of d4T, 3TC and EFV in 600 ARV-naive participants with HIV infection. Following the completion of the double-blind portion of the trial, there was an additional 2 year single arm open label portion of the trial in selected sites, wherein all patients received TDF, 3TC, and EFV as once daily regimen. (Patients originally randomized to the d4T arm switched to receive TDF.)

In a 144-week analysis, when missing observations in the ITT population were treated as having plasma HIV-1 RNA concentrations greater than 400 copies/mL, 76% of participants in the TDF group and 72% of participants in the d4T active control group achieved plasma HIV-1 RNA concentrations < 400 copies/mL. Plasma HIV-1 RNA concentrations < 50 copies/mL at week 144 were seen in 73% and 69% of participants in the TDF and d4T active control groups, respectively. The mean increases in CD4+ cell count from baseline to week 144 were 263 and 283 cells/mm^3 for the TDF and d4T active control groups, respectively.

Tenofovir-Emtricitabine Compared to Zidovudine-Lamivudine

Gilead Study 934 was a phase III, randomized, open-label, multicenter study designed to compare a regimen of EFV with either TDF 300 mg/FTC 200 mg QD or ZDV 300 mg/3TC 150 mg BID as FDC Combivir®.

Interim analysis at 48 weeks revealed discontinuation occurred more frequently in the ZDV/3TC group (9%) than TDF/FTC (4%), mostly because of AEs such as anemia and nausea (85). The 48-week data demonstrated that using the time to loss of virologic failure as the primary analysis in which missing or switch is counted as a failure, the proportion of participants with plasma HIV-1 RNA levels less than 400 copies/mL in an ITT population (n = 487) was 84% in the TDF/FTC group compared to 73% in the ZDV/3TC-treated participants (p = 0.002). The proportion of participants with plasma HIV-1 RNA levels < 50 copies/mL was 80% in the TDF/FTC group versus 70% in the ZDV/3TC group (p = 0.021). These results were supported by 96 week data (86).

Safety Profile for TDF

More than 10,000 patients have received TDF 300 mg once daily alone or in combination with other ARVs in phase I-IV clinical trials. Over 11,000 patients have received TDF in expanded access programs. The cumulative patient exposure to marketed TDF from first approval to December 31, 2008 is estimated to be approximately 1,200,000 patient-years of treatment.

TDF is FDA pregnancy category B. In studies of rats and rabbits at 14-19 times the exposure in humans, there was no evidence of impaired fertility or increased risk of malformations. Among cases reported to the APR, birth defects were seen in 11 (2.2%) of 491 first trimester exposures (95% CI 1.1-4.2) and in 4 (1.3%) of 309 later exposures during pregnancy (95% CI 0.4- 3.3).

In Gilead Study 903, Tenofovir (TDF) and stavudine (d4T) had comparable renal safety profiles with no patient in the TDF arm discontinuing the study for a renal-related abnormality and less than one percent of patients in each arm experiencing serum creatinine levels of more than 2 mg/dL. Toxicities that have been attributed to mitochondrial toxicity (peripheral neuropathy, lipodystrophy, and lactic acidosis) were reported in 100 patients, 83 (28%) of 301 in the d4T group and 17 (6%) of 299 in the TDF group ($p < .001$). Neuropathy was observed in 31 (10%) of 301 and 9 (3%) of 299 patients in the d4T and TDF groups, respectively ($p < .001$). Investigator-defined lipodystrophy was reported more often in patients receiving d4T vs. TDF (58 [19%] of 301 vs. 9 [3%] of 299, respectively; $p < .001$).

Using whole body dual energy X-ray absorptiometry (DXA), significantly less total limb fat was observed in the d4T group at week 96 (7.9 kg TDF [n = 128] vs. 5.0 kg d4T [n = 134], $p< .001$) and week 144 (8.6 kg TDF [n = 115] vs. 4.5 kg d4T [n = 117], $p< .001$). Mean decreases in lumbar spine and hip bone mineral densities after three years of treatment were less than three percent in both arms of the study. Bone mineral density reduction observed in Study 903 was non-progressive, with no substantial changes from the 24- and 48-week intervals to week 144. At 144 weeks, a total of five fractures were observed in the TDF arm compared to eleven fractures in d4T-treated patients.

Further information can be found in the TDF Investigator Brochure and the most recent Viread® package insert.

Safety Data on TDF in pregnancy

Chronic dosing of rats in pregnancy noted no growth, or reproductive problems when TDF was administered at doses not associated with maternal toxicity. At high doses of exposure (25 time the AUC achieved with therapeutic dosing), no fetal structural changes were seen.

Chronic exposure of fetal monkeys to TDF at a high dose of 30 mg/kg (25 times the AUC levels achieved with therapeutic doses in humans) from days 20-150 of gestation did not result in gross structural abnormalities (Tarantal 2002). However significantly lower fetal circulating insulin-like growth factor levels were reported and were associated with body weights 13% lower than untreated controls. A slight reduction in fetal bone porosity was also observed within 2 months of maternal treatment. However, a macaque treated for over 10 years with 10 mg/kg/day of TDF has given birth over several years to three infant macaques, all of whom were normal and had no bone abnormalities at birth (Van Rompay 2008).

TDF is designated as FDA pregnancy Category B based on animal and clinical data. In the APR, sufficient numbers of first trimester TDF exposures have been monitored to detect at least a 2 fold increase in risk of overall birth defects but no such increase in birth defects has been observed. Through January 31, 2008, the APR contains data on tenofovir use in pregnancies resulting in 800 live births, most to women with HIV, and mostly used in combination with other antiretrovirals. Rates of congenital abnormalities in women who used tenofovir during pregnancy were comparable to those in the US Centers for Disease Control and Prevention (CDC) population-based birth defects surveillance system (2.72 per 100 live births) and were similar to rates associated with other antiretroviral drugs (ARVs) in the APR. The prevalence of birth defects after first trimester TDF exposure was 11 (2.2%) of 491 (95%CI 1.1-4.0%).

Studies of intravenous TDF administration in pregnant cynomolgus monkeys reported a fetal/maternal concentration of 17% indicating some placental transfer.(Tarantal 1999) In three studies of pregnant women the cord to maternal blood ratio ranged from 0.60 to 0.99 indicating high placental transfer (Rodman 2006; Burchett 2007; Hirt 2008). A dose of 600 mg of TDF in labor resulted in levels in the women similar to levels in non-pregnant adults after a 300 mg dose; suggesting higher doses are required for adequate levels in term pregnant women (Hirt 2008). In PACTG 394, administration of 900 mg TDF and 600 mg FTC (as 3 Truvada tablets) during active labor in 15 women resulted in a favorable safety profile. Pharmacokinetic assessments demonstrated TFV Cmax increased by 83% compared to 600 mg dosing but AUC was similar (Flynn 2009).

TDF pharmacokinetics during pregnancy among 19 pregnant women were assessed in P1026s in the last trimester between weeks 30-36 and also at 6-12 weeks post delivery. The proportion of pregnant women with AUC exceeding the target of 2 µg hour/ml was slightly lower in the third trimester (74%) than post partum (86%) but trough levels were comparable at both time points. A recent case series found TDF to be well tolerated among 76 pregnant women, with two stopping therapy, one for rash and one for nausea. All 78 infants were healthy with no signs of toxicity, and all were HIV-uninfected (Habert 2008).

Emtricitabine and Tenofovir Disoproxil Fumarate Fixed Dose Combination Tablet (FTC/TDF, Truvada®)

Gilead Sciences developed Truvada, a product containing FTC 200 mg and TDF 300 mg in a fixed-dose combination (FDC) tablet formulation. A New Drug Application (NDA) for the FDC was filed with the US FDA on March 12, 2004 and was approved on August 2, 2004. As a component of the NDA, two phase I studies evaluating the pharmacokinetics of co-administered FTC and TDF tablet formulation have been completed.

Overall, Study GS-US-104-172 demonstrated bioequivalence between the FTC/TDF combination tablet and the FTC capsule and TDF tablet formulations when administered separately. Administration of the FTC/TDF combination tablet with either a high-fat meal or light meal increased tenofovir exposure by approximately 30% compared with fasted-state administration. Clinical experience with TDF indicates that the effect of food on tenofovir exposure is not of clinical relevance. FTC and TDF, either administered as a combination tablet (containing FTC 200 mg/ TDF 300 mg) or co-administered as FTC 200 mg capsule and TDF 300 mg tablet were well tolerated.

Hepatitis B Virus (HBV)

Exacerbations of HBV have been reported in patients after discontinuation of FTC or TDF. Patients, who are co-infected with HBV, may have increased values on liver function tests and exacerbation of hepatitis symptoms when FTC or TDF is stopped. Usually these symptoms are self-limiting; however, serious complications have been reported. The causal relationship to FTC or TDF discontinuation is unknown. Patients co-infected with HBV and HIV should be closely monitored with both clinical and laboratory assessments follow-up for several months after stopping FTC or TDF treatment.

Additional information can be found in the most recent Emtriva, Viread®, or Truvada® package insert.

Because the second-line drugs to be used in this study are not dictated by the protocol, the background on and safety profile of all possible drug choices are not detailed here. Information on each drug can be found in its package insert.

2.0 STUDY OBJECTIVES

2.1 Primary Objective and Endpoint

To determine whether continuation of HAART (Arm A) after delivery or other pregnancy outcome reduces morbidity and mortality compared to discontinuation and re-initiation of HAART according to current standards of care (Arm B). The primary combined endpoint includes death, AIDS-defining illness, and serious non-AIDS-defining cardiovascular, renal, and hepatic events.

2.2 Secondary Objectives

2.2.1 To determine, characterize, and compare the rates of AIDS-defining and HIV-related illnesses, opportunistic infections, immune reconstitution inflammatory syndromes, and other targeted medical conditions (see Appendix II), with regard to outcomes and survival.

2.2.2 To assess toxicity, both selected moderate (Grade 2) laboratory abnormalities (renal, hepatic, and hematologic) and severe (Grade 3 or Grade 4) laboratory values and signs and symptoms.

2.2.3 To compare emergence of HIV-1 resistance to ART during the 1st, 2nd, and 3rd years following randomization and at the end of the study.

2.2.4 To evaluate rates of self-reported adherence to HAART and its association with the primary endpoint and with CD4+ cell count, HIV-1 viral load, and HIV-1 resistance patterns at 1, 2, and 3 years following randomization.

2.2.5 To compare quality of life measurements between the study arms at 1, 2, and 3 years following randomization.

2.2.6 To investigate changes in plasma concentrations of inflammatory and thrombogenic markers between arms and to correlate these markers to clinical events.

2.2.7 To evaluate cost effectiveness and feasibility of the trial strategies.

3.0 STUDY DESIGN

This is a randomized strategy trial conducted among women who received HAART during pregnancy for PMTCT but do not otherwise meet criteria to initiate HAART for their own health. Potential participants may be identified/recruited and consented during pregnancy or after delivery or other pregnancy outcome. Study-specific screening may be initiated in the third trimester or after pregnancy outcome. Women who are screened for the study will be counseled to continue their HAART until they are randomized. Randomization will occur within 0-42 days after pregnancy outcome. Women who do not carry their pregnancy to the third trimester but otherwise meet study eligibility criteria may be enrolled.

<u>Step 1</u>

Participants will be randomized within 0-42 days after delivery or other pregnancy outcome to one of two study arms:

Arm A: Continuation of HAART
Arm B: Discontinuation of HAART

<u>Step 2</u>

Participants in Step 1 will be registered to Step 2 if they develop an indication for antiretroviral therapy as defined in Section 6.3.2 and otherwise meet Step 2 eligibility criteria per Sections 4.3 and 4.4.

<u>Step 3</u>

Participants in Step 1, Arm A or Step 2 will be registered to Step 3 if they meet therapeutic indications to change HAART as defined in Section 6.3.1 and otherwise meet Step 3 eligibility criteria per Sections 4.5 and 4.6.

4.0 SELECTION AND ENROLLMENT OF PARTICIPANTS

4.1 Inclusion Criteria: Step 1

4.1.1 Women age ≥ 18 years or who have attained the minimum age of independent consent, as defined by the local IRB, and are willing and able to provide written informed consent

Additionally, at sites with IRB approval to enroll younger participants, women age 16-17 years who are willing and able to provide written assent and whose parent or legal guardian is willing and able to provide written informed consent

4.1.2 Confirmed HIV infection, documented by positive results from two samples collected at different time points prior to study entry:

- Sample #1 may be tested in a non-study laboratory (e.g., laboratories used by participant's non-study medical providers and/or by PEPFAR and other public health programs). However, documentation of both the result and the sample collection date must be available for filing in the participant's study chart. If such documentation is not available, Sample #1 should be tested in a CAP/CLIA approved laboratory (for US sites) or a laboratory that operates according to good clinical laboratory practice (GCLP) guidelines, participates in appropriate external quality assurance programs, and is approved by the IMPAACT Central Laboratory (for non-US sites).

  Sample #1 may be tested using any of the following:

  - Two rapid antibody tests from two different manufacturers or based on different principles and epitopes
  - One EIA OR Western blot OR immunofluorescence assay OR chemiluminescence assay
  - One HIV DNA PCR
  - One quantitative HIV RNA PCR (>5,000 copies/mL)
  - One qualitative HIV RNA PCR
  - One total HIV nucleic acid test

- Sample #2 must be tested in a CAP/CLIA approved laboratory (for US sites) or a laboratory that operates according to GCLP guidelines, participates in appropriate external quality assurance programs, and is approved by the IMPAACT Central Laboratory.

  Sample #2 may be tested using any of the following:

  - One EIA confirmed by Western blot OR immunofluorescence assay OR chemiluminescence assay
  - One HIV DNA PCR
  - One quantitative HIV RNA PCR (>5,000 copies/mL)
  - One qualitative HIV RNA PCR
  - One total HIV nucleic acid test

4.1.3 Documentation of hepatitis B surface antibody (HBsAb) status and hepatitis B surface antigen (HBsAg) status (if antibody is negative) within 12 months prior to study entry

NOTE: When HBsAg and HBsAb status are not documented in medical records within 12 months prior to study entry, testing should be performed as part of the study screening process. Sites may choose to perform HBsAb testing first and then perform HBsAg testing only for those who test HBsAb negative. Alternatively, sites may test for HBsAg first; in this case, HBsAb testing is not required.

4.1.4 Within 0-42 days after pregnancy outcome

4.1.5 Antiretroviral treatment naïve, defined as < 14 days of one or more antiretroviral agents, prior to therapy initiated during current pregnancy

NOTE: History of receipt of ARV for PMTCT during prior pregnancies and immediately postpartum is permitted. Receipt of single ARV regimens in prior pregnancies is permitted, as is receipt of a single ARV regimen prior to initiation of a triple ARV (HAART) regimen in the current pregnancy.

4.1.6 Receipt of at least four weeks of HAART prior to study entry, at least two weeks of which must have been prior to pregnancy outcome (up to seven consecutive days of missed therapy is permitted).

NOTE: HAART is defined as three or more agents from two or more classes of antiretroviral therapy (ART). Women on a triple nucleoside regimen during pregnancy are eligible but should be switched to a regimen meeting the study definition of HAART if randomized to continue HAART (Arm A). See also Section 5.1.1 below.

4.1.7 CD4+ cell count ≥ 400 cells/mm^3 on a specimen obtained within 120 days prior to initiation of HAART for current pregnancy

NOTE: This result will be obtained from the participant's medical records.

4.1.8 CD4+ cell count ≥ 400 cells/mm^3 on a specimen obtained on HAART and within 45 days prior to study entry

4.1.9 The following laboratory values on a specimen obtained within 45 days prior to study entry:

- Absolute neutrophil count (ANC) ≥ 750/mm^3
- Hemoglobin ≥ 7.0 g/dL
- Platelet count ≥ 50,000/mm^3
- AST (SGOT), ALT (SGPT), and alkaline phosphatase ≤ 2.5 x ULN

4.1.10 Estimated creatinine clearance of ≥ 60mL/min within 45 days prior to entry using the following formula:
{[140 – Age (yr) x [weight (kg)] ÷ [72 x serum Cr(mg/dL)]} x 0.85

4.1.11 Intent to remain in current geographical area of residence for the duration of the study

4.1.12 Willingness to attend study visits as required by the study

4.2 Exclusion Criteria: Step 1

4.2.1 Previous participation in PROMISE (P1077)

4.2.2 Clinical indication for HAART including any WHO Clinical Stage 3 or 4 condition, prior or current tuberculosis disease (a positive PPD test alone is not considered exclusionary), and/or any other clinical indication per country-specific treatment guidelines

4.2.3 Clinically significant illness or condition requiring systemic treatment and/or hospitalization within 30 days prior to study entry

NOTE: Clinical significance should be assessed based on the opinion of the site investigator. Women may be enrolled after completion of therapy or after being assessed as clinically stable on therapy, in the opinion of the investigator, provided completion of therapy or assessment as clinically stable on therapy occurs at least 30 days prior to study entry.

4.2.4 Social or other circumstances which, in the opinion of the site investigator, would hinder long-term follow up

4.2.5 Use of any prohibited medications within 14 days prior to study entry (refer to the study MOP for a list of prohibited medications)

4.2.6 Current compulsory detention (involuntary incarceration) in a correctional facility, prison, or jail for legal reasons or compulsory detention in a medical facility for treatment of either a psychiatric or physical (e.g., infectious disease) illness

4.2.7 Currently breastfeeding or planning to breastfeed

4.2.8 Current documented conduction heart defect (specialized assessments to rule out this condition are not required; a heart murmur alone and/or type 1 second-degree atrioventricular block (also known as Mobitz I or Wenckebach) is not considered exclusionary)

4.2.9 Known evidence of HBV DNA levels >2000 IU/mL (approximately 10,000 copies/mL) in the presence of elevated (grade 1 and higher) ALT (HBV DNA testing is not required for study screening or enrollment but should be considered to determine whether treatment for HBV is indicated)

4.3 Inclusion Criteria: Step 2

4.3.1 On Step 1 of the study

4.3.2 Develops an indication for HAART as defined in Section 6.3.2

4.3.3 Willing and able to reinitiate HAART (if on Step 1, Arm B) or to continue HAART (if on Step 1, Arm A)

4.4 Exclusion Criteria: Step 2

None

4.5 Inclusion Criteria: Step 3

4.5.1 On Step 1, Arm A or Step 2 of the study

4.5.2 Meets therapeutic indications to change HAART as defined in Section 6.3.1

4.5.3 Willing and able to continue HAART on an alternate regimen

4.6 Exclusion Criteria: Step 3

4.6.1 On Step 1, Arm B

4.7 Enrollment Procedures

4.7.1 Protocol Registration

All sites must have a site implementation plan (SIP) that has been approved by the 1077HS protocol team. The SIP should detail the arrangements for following women on HAART after delivery or other pregnancy outcome. Transfer from an enrolling IMPAACT site to a co-located ACTG site for long term follow-up is encouraged and should be included as part of the SIP. Each site's SIP must be approved prior to submission of protocol registration documents (described below).

Prior to implementation of this protocol, and any subsequent full version amendments, each site must have the protocol and informed consent forms (ICFs) approved by their local institutional review board/ethics committee (IRB/EC) and any other applicable regulatory entity (RE). Upon receiving final approval, sites will submit all required protocol registration documents to the DAIDS Protocol Registration Office (PRO) at the Regulatory Support Center (RSC). The DAIDS PRO will review the submitted protocol registration packet to ensure that all of the required documents have been received.

Site-specific ICFs will be reviewed and approved by the DAIDS PRO and sites will receive an Initial Registration Notification from the DAIDS PRO that indicates successful completion of the protocol registration process. A copy of the Initial Registration Notification should be retained in the site's regulatory files.

Upon receiving final IRB/EC and any other applicable RE approvals for a full protocol amendment, sites should implement the amendment immediately. Sites are required to submit an amendment registration packet to the DAIDS PRO at the RSC. The DAIDS PRO will review the submitted protocol registration packet to ensure that all the required documents have been received. Site-specific ICFs will not be reviewed and approved by the DAIDS PRO and sites will receive an Amendment Registration Notification when the DAIDS PRO receives a complete registration packet. A copy of the Amendment Registration Notification should be retained in the site's regulatory files.

For additional information on the protocol registration process and specific documents required for initial and amendment registrations, refer to the current version of the DAIDS Protocol Registration Manual.

4.7.2 Screening and Enrollment/Randomization of Participants

After obtaining an Initial Registration Notification from the DAIDS PRO, and meeting all other study activation requirements, each site will be issued a site-specific study activation notice. At each site, recruitment and screening of potential participants may begin upon receipt of the activation notice.

Potential participants may be identified/recruited and consented during pregnancy or after pregnancy outcome. Once a potential participant has been identified, details will be carefully discussed with her and she will be asked to read and sign the approved site-specific ICF. If the participant is unable to read, the process for consenting illiterate participants, as defined by the local IRB, should be followed. Written informed consent for study participation must be obtained before any study-specific procedures are performed.

For all potential participants who provide written informed consent, a Screening Checklist must be entered through the DMC Subject Enrollment System.

- For potential participants who provide informed consent but are determined to be ineligible, or who do not enroll in the study for any reason, a Screening Failure Results form must be completed and keyed into the database.

- For potential participants who provide informed consent and are determined to be eligible, enrollment/randomization is done through the DMC Subject Enrollment System according to standard data management procedures. The appropriate enrollment screen for this component of PROMISE is identified as 1077HS.

4.8 Co-enrollment Guidelines

US sites are encouraged to co-enroll participants in ACTG A5128. Co-enrollment in A5128, "Plan for Obtaining Informed Consent to Use Stored Human Biological Materials (HBM) for Currently Unspecified Analyses," does not require permission from the 1077HS protocol chairs. Likewise, sites may also co-enroll participants in IMPAACT P1025, "Core Perinatal Protocol" and IMPAACT P1026s, "Pharmacokinetic Properties of Antiretroviral Drugs During Pregnancy", without permission from the 1077HS protocol chairs. When co-enrolling in IMPAACT P1026s, the P1026s postpartum pk sampling should be completed prior to the participant's enrollment in 1077HS.

Non-US sites are encouraged to co-enroll participants in ACTG A5243. Co-enrollment in A5243, "Plan for Obtaining Human Biological Samples at Non-US Clinical Research Sites for Currently Unspecified Genetic Analyses," does not require permission from the 1077HS protocol chairs.

Co-enrollment in P1078, "A Phase IV Randomized Double-Blind Placebo-Controlled Trial to Evaluate the Safety of Immediate (Antepartum-Initiated) Versus Deferred (Postpartum-Initiated) Isoniazid Preventive Therapy Among HIV-Infected Women in High Tuberculosis (TB) Incidence Settings" is not permitted and PROMISE participants are discouraged from participating in other clinical trials of investigational agents during the time of the trial. Co-enrollment in other clinical trials may be considered on a case-by-case basis and requires the approval of the protocol chairs of both trials.

5.0 STUDY TREATMENT

5.1 Drug Regimens, Administration, and Duration

The preferred study-supplied HAART regimen is Lopinavir/Ritonavir (LPV/RTV) plus fixed dose combination Emtricitabine/Tenofovir disoproxil fumarate (FTC/TDF). Additional study-supplied ARVs available for use in this study include fixed dose Lamivudine/Zidovudine (3TC/ZDV), Lamivudine (3TC), Zidovudine (ZDV), Tenofovir disoproxil fumarate (TDF), fixed dose combination Emtricitabine/Tenofovir disoproxil fumarate/Rilpivirine (FTC/TDF/RPV), Didanosine (ddI), Atazanavir (ATV), Raltegravir (RAL), and Ritonavir (RTV). However, all study-supplied ARVs may not be available at all study sites; availability will be based on the status of drug regulatory approval for each ARV in each country.

While LPV/RTV plus TDF/FTC is the preferred study-supplied regimen, the study clinicians in conjunction with participants should determine the optimal drug combination for each participant. Likewise, the specific second-line and subsequent regimens are not defined by this protocol, and should be determined at the discretion of the study clinicians. Regimens not provided by the study may be used if they meet the study definition of HAART, i.e., three or more agents from two or more classes of antiretroviral therapy, and are provided by prescription.

All ARVs should be prescribed consistent with current package inserts. Fixed dose FTC/TDF/RPV may be used as an alternative first line regimen for participants who are not able tolerate or adhere to LPV/RTV or ATV/RTV. FTC/TDF/RPV has thus far only been studied as a first line regimen and use in postpartum women has been limited; the frequency and severity of adverse events experienced by women on FTC/TDF/RPV in this study may therefore differ from experience described in the package insert. Consultation with the CMC is required in advance of prescribing FTC/TDF/RPV for any study participant.

5.1.1 Step 1, Arm A

Women on Arm A may continue to receive the ART regimen that they were receiving prior to study entry as long as it meets the protocol definition of HAART and they have continued access to the drugs or they may opt to receive study-supplied HAART.

Women on Arm A who were on a triple nucleoside regimen during pregnancy should be switched to a regimen meeting the study definition of HAART. Should a site investigator determine that continuing a triple nucleoside regimen, rather than switching to a study-defined HAART regimen, would be in the best interest of a participant, consultation with the study Clinical Management Committee (CMC) is required.

Women on Arm A should initiate their study regimen within 72 hours after randomization.

5.1.2 Step 1, Arm B

Women on Arm B should discontinue the HAART regimen they were taking during pregnancy within 72 hours after randomization. However, if a woman on Arm B was receiving an NNRTI as part of her HAART regimen during pregnancy, she may remain on the NRTI component of the regimen for seven days after discontinuing the NNRTI to reduce the chance of developing resistance.

5.1.3 Step 2

Step 1, Arm A participants may enter Step 2 and continue HAART if they develop an indication for antiretroviral therapy as defined in Section 6.3.2 and otherwise meet Step 2 eligibility criteria (see Sections 4.3 and 4.4) but do not meet criteria to enter Step 3 and switch to a second line regimen.

Step 1, Arm B participants may enter Step 2 and reinitiate HAART if they develop an indication for antiretroviral therapy as defined in Section 6.3.2 and otherwise meet Step 2 eligibility criteria (see Sections 4.3 and 4.4). For these participants, Step 2 entry evaluations should be completed prior to the first dose of HAART.

Step 2 participants may receive study-supplied ART or they may receive ART of their choice from outside the study, provided the regimen meets the protocol definition of HAART.

Preferred Study-Supplied Regimen

- Emtricitabine/Tenofovir disoproxil fumarate (FTC/TDF) fixed dose combination tablet 300 mg/200 mg orally daily
  PLUS ONE OF THE FOLLOWING:
  - Lopinavir/Ritonavir (LPV/RTV) fixed dose combination tablet 200 mg/50 mg x 2 tablets for a dose of 400mg/100mg orally twice daily. Total daily dose is 800mg LPV and 200mg RTV; or
  - Lopinavir/Ritonavir (LPV/RTV) fixed dose combination tablet 200 mg/50 mg x 4 tablets for a dose of 800mg/200mg orally once daily. Total daily dose is 800mg LPV and 200mg RTV.

5.1.4 Step 3

Step 1, Arm A or Step 2 participants who meet therapeutic indications to change HAART as defined in Section 6.3.1 may enter Step 3 and initiate a second-line regimen of three or more ARV drugs chosen by the site investigator. Step 3 entry evaluations should be completed prior to the first dose of the second-line regimen.

5.2 Drug Administration

All study drugs may be given with or without food, with the exception of Atazanavir (ATV) and Emtricitabine/Tenofovir disoproxil fumarate/Rilpivirine (FTC/TDF/RPV), which must be given with food.

5.3 Formulations of Study-Supplied Drugs

Generic name<br>Abbreviation<br>Trade name	Formulation	Appearance	Storage
Zidovudine<br>ZDV<br>Retrovir®	300 mg tablets	Biconvex, white, round, film-coated tablets	15-25°C (59-77°F).
Lamivudine<br>3TC<br>Epivir®	300 mg tablets	Gray, modified diamond-shaped, film-coated tablets	25°C (77°F). Excursions permitted to 15-30°C (59-86°F). See USP Controlled Room Temperature.
Lamivudine/Zidovudine<br>3TC/ZDV<br>Combivir®	150 mg/300 mg tablets	White, modified capsule shaped, film-coated tablets	2-30°C (36-86°F).
Tenofovir Disoproxil Fumarate<br>TDF<br>Viread®	300 mg tablets	Almond-shaped, light blue, film-coated tablets	25°C (77°F). Excursions permitted to 15-30°C (59-86°F). See USP Controlled Room Temperature.
Tenofovir/Emtricitabine<br>TDF/FTC<br>Truvada®	300 mg/200 mg tablets	Blue, capsule shaped, film-coated tablets	25°C (77°F). Excursions permitted to 15-30°C (59-86°F). Keep container tightly closed. Each bottle contains a silica gel desiccant canister that should remain in the original container to protect the product from humidity. See USP Controlled Room Temperature.
Tenofovir/Emtricitabine/<br>Rilpivirine<br>TDF/FTC/RPV<br>Complera®	300 mg/200 mg/<br>25 mg<br>tablets	Purplish-pink, capsule-shaped, film-coated tablets with "GSI" on one side	25°C (77°F). Excursions permitted to 15-30°C (59-86°F). See USP Controlled Room Temperature. Keep container tightly closed.
Lopinavir/Ritonavir<br>LPV/RTV<br>Kaletra®<br>Aluvia®	200 mg/50 mg tablets	Ovaloid, film-coated tablets that will be either red or yellow	20-25°C (68-77°F). Excursions permitted to 15-30°C (59-86°F). See USP Controlled Room Temperature.
Atazanavir<br>ATV<br>Reyataz®	300 mg capsules	Capsules with red cap and blue body	25°C (77°F). Excursions permitted between 15-30°C (59-86°F).
Ritonavir<br>RTV<br>Norvir®	100 mg tablets	White, film-coated ovaloid tablets	20-25°C (68-77°F). Excursions permitted between 15-30°C (59-86°F). See USP Controlled Room Temperature.
Didanosine<br>ddI<br>Videx®	400 mg and 250 mg capsules	White, opaque, delayed-release capsules	25°C (77°F). Excursions permitted between 15-30°C (59-86°F). See USP Controlled Room Temperature. Store in tightly closed containers.
Raltegravir<br>RAL<br>Isentress®	400 mg tablets	Pink, oval-shaped, film-coated tablets with "227" on one side	20-25°C (68-77°F). Excursions permitted to 15-30°C (59-86°F). See USP Controlled Room Temperature.

5.4 Drug Supply, Distribution, and Pharmacy

LPV/RTV and RTV will be provided by Abbott Laboratories. ATV and ddI will be provided by Bristol-Myers Squibb. TDF/FTC/RPV, TDF/FTC, and TDF will be provided by Gilead Sciences. ZDV/3TC, 3TC, and ZDV will be provided by GlaxoSmithKline/ViiV. RAL will be provided by Merck and Company, Inc. As noted in Section 5.1, all study-supplied ARVs may not be available at all sites. ART that is not provided by the study may be used if it meets the study definition of HAART and is provided by prescription.

The study products provided through this study will be distributed to the study sites by the NIAID Clinical Research Products Management Center (CRPMC). The Clinical Research Site Pharmacist of Record can obtain the study products for this protocol by following the instructions provided in the latest version of the Pharmacy Guidelines and Instructions for DAIDS Clinical Trials Networks in the Study Product Management Responsibilities section.

The Clinical Research Site Pharmacist of Record is required to maintain records of all study products received, dispensed to study participants, and final disposition of all study products. All unused study products at US sites must be returned to the NIAID CRPMC after the study is completed or terminated. The Clinical Research Site Pharmacist of Record at non-US clinical research sites must follow the instructions in the Pharmacy Guidelines and Instructions for DAIDS Clinical Trials Networks (DAIDS Pharmaceutical Affairs) for the destruction of unused study products in the Study Product Management Responsibilities section and following the instructions for the Study Product Destruction Form.

5.5 Concomitant Medications Guidelines

Sites must refer to the most recent study medication's package insert or investigator's brochure to access additional current information on prohibited and precautionary medications. To avoid drug interaction and adverse events, the manufacturer's package inserts of the antiretroviral and concomitant agent should be referred to whenever a concomitant medication is initiated or dose changed.

Concomitant use of ingested traditional medicines is strongly discouraged while participants are taking HAART.

Information on drugs without trade names, with many marketed forms, or those not available in the US may be found at:
http://www.hiv-druginteractions.org/Interactions.aspx
http://www.nccc.ucsf.edu/hiv_clinical_resources/pharmacy_central

5.6 Prohibited Medications

Medications that are prohibited with study-supplied drugs are listed in the MOP.

5.7 Precautionary Medications

Medications that should be used cautiously with study-supplied drugs are listed in the MOP.

5.8 Contraception

Counseling regarding contraception is the standard of care for women during pregnancy, early postpartum, and postpartum. All women on study will be provided contraception counseling as well as counseling on the importance of condom use for the prevention of HIV and other sexually transmitted infections, even if other contraceptives are used. Although one recent observational study (89A) provided evidence of a relationship between injectable hormonal contraception and increased female-to-male HIV transmission, current WHO recommendations reinforce that women living with HIV can continue to use all existing hormonal contraceptive methods without restriction (89B). Study sites are encouraged to provide a range of contraceptive options on site or to facilitate referrals for contraceptive services. Women in the study will be asked about their contraceptive choices and this information will be recorded on the GYN case report form.

Site staff should refer to the latest US package insert for information regarding contraceptives and possible drug interactions with the drugs in the HAART regimen. For example, if the regimen includes EFV (a class D drug), participants must agree to use two reliable methods of contraception: a barrier method of contraception (e.g., condoms, diaphragm, or cervical cap with or without spermicide) together with another reliable form of contraceptive (e.g., a second barrier method, an IUD, or a hormone-based contraceptive) while receiving EFV and for 12 weeks after stopping EFV.

Antiretrovirals such as NVP, LPV/RTV, NFV, and RTV decrease ethinyl estradiol levels and norethindrone levels, which may affect the efficacy of hormonal contraceptives. ATV, indinavir (IDV), and EFV increase levels of ethinyl estradiol and norethindrone, which may accentuate their side effects/toxicity. Because of the varied interactions between oral hormonal contraceptives and the different classes of ART with resulting unknown effects on contraceptive efficacy, oral contraceptives should be used with caution in HIV-seropositive women. Also, the effects of oral contraceptives on the levels of ART drugs are unknown but could alter treatment efficacy. Consideration should be given to alternative methods or to use of back-up forms of contraception. No clinically significant interactions were seen between depot medroxyprogesterone and nucleoside agents NVP, EFV, or NFV in the ACTG 5093 study, suggesting that this agent may be a reasonable contraceptive choice for women on ART (90). The long term effects regarding risk of HIV progression or other metabolic effects of contraceptive use in HIV+ women are not well known.

6.0 SUBJECT MANAGEMENT

6.1 Timing of Evaluations

6.1.1 Screening Evaluations

After informed consent is obtained, study-specific screening evaluations may be performed beginning in the third trimester of pregnancy or after delivery or other pregnancy outcome. All screening evaluations must be performed within 45 days prior to entry. If required screening laboratory test results dated within 45 days prior to entry are available and adequately documented in participant medical records, these results may be used for study purposes, and need not be repeated after obtaining informed consent. Reassessment of lab values and clinical conditions may be required, for example, if too much time passes after the initial evaluations and before study entry or if the investigator believes that exclusionary abnormalities identified initially may no longer be present. Participants will be counseled at the time of screening to continue to take their HAART regimen until they are randomized.

In addition to collection of data on women who are enrolled in the study, demographic, clinical and laboratory data on screening failures will also be captured in a screening log and entered into the IMPAACT/ACTG database.

6.1.2 Entry Evaluations

If a woman is found to be eligible based on the screening evaluations, she will be randomized at the entry visit, and additional assessments will be performed as shown in the Schedule of Evaluations (SOE).

Participants who are randomized to Arm A who want to switch to a study-provided regimen are to do so within 72 hours after randomization.

Participants randomized to Arm B are to discontinue treatment within 72 hours after randomization; however, if a woman is receiving an NNRTI as part of her HAART regimen during pregnancy and is randomized to discontinue HAART, she may remain on the NRTI component of the regimen for seven days after discontinuing the NNRTI to reduce the chance of developing resistance.

6.1.3 Post-Entry Evaluations

Evaluations must occur after the entry visit.

In Step 1, post-entry visits are scheduled to take place 4 weeks after entry, 12 weeks after entry, and every 12 weeks (q12) thereafter. The target dates for all visits are counted from the date of study entry (date of entry = day 0).

For participants who enter Step 2 or Step 3, an additional Step Entry visit is required, followed by a Step Change Week +4 visit. The Step Change Week +4 visit should take place four weeks after Step Entry. Thereafter, the q12 week visit schedule established in Step 1 (based on the date of study entry) should be resumed. If the target date for the Step Change Week +4 visit falls within the targeted window of the next q12 visit (defined below), a combined visit should be conducted. In the case of a combined visit, all required evaluations for both the Step Change Week +4 visit and the q12 visit should be performed only once and only one set of case report forms (CRFs) should be completed to record the evaluations.

All study visits should be conducted per the schedule specified in the SOE ±14 days. That is, the targeted visit window is ±14 days for all post-entry visits. In addition, for all post-entry visits other than the Step 1 Week 4 visit and the Step Change +4 visit, an allowable window of ±28 days is specified. *Every effort should be made to conduct all visits within the targeted window;* however, visits are permitted to be conducted within the allowable window.

NOTE: In the event that a scheduled visit cannot be conducted within the allowable window, study sites may consult the protocol team on possible options for participant follow-up, including options for conducting visits outside the allowable window. Visits conducted outside allowable window will be considered deviations from the protocol-specified visit schedule. As such, the protocol team cannot approve the conduct of such visits per se; nonetheless the team acknowledges that conducting such visits, rather than waiting for the next scheduled visit window to open, may be in the best interest of the participant as well as the study. In the event that visits are conducted outside of the allowable window, the deviation from the protocol-specified visit schedule should be documented in the participant's study records and every effort should be made to conduct the participant's next scheduled visit as close as possible to the target date for the visit.

6.1.4 Discontinuation Evaluations

Premature Discontinuation of Therapy Evaluations
Participants who prematurely discontinue ART (whether study-provided or locally-provided) should complete the “Premature Treatment D/C” evaluations listed in the SOE, ideally within 14 days after stopping study drugs. They will be asked to continue off-therapy/on-study and receive all study evaluations per the SOE, through to completion of the study.

Study Discontinuation Evaluations
All participants should complete the “Study D/C” evaluations listed in the SOE at their final study visits. For participants who choose to prematurely discontinue participation in the study for any reason, these evaluations should ideally be completed within 30 days of the decision to withdraw from the study.

6.1.5 Final Study Contacts

Given that Study Discontinuation visits will include laboratory testing, a final contact will be required to provide final study test results to participants, as well as referrals to non-study care sources, if applicable. In addition, for participants who are pregnant at the time of their final study visits, an additional contact will be required to ascertain the pregnancy outcome.

6.2 Instructions for Evaluations

All clinical and laboratory information required by this protocol is to be present in the source documents. Sites must comply with the DAIDS policy on Requirements for Source Documentation in DAIDS Funded and/or Sponsored Clinical Trials; refer to the operational guidance provided in the appendix of this policy, entitled Source Documentation Requirements. The policy and appendix are available at:

www.niaid.nih.gov/LabsAndResources/resources/DAIDSClinRsrch/pages/clinicalsite.aspx

The results of all study-specified evaluations are to be recorded on the CRFs and keyed into the database unless otherwise specified.

6.2.1 Documentation of HIV-1 Infection

Confirmed HIV infection must be documented prior to study entry per Inclusion Criterion 4.1.2.

6.2.2 Medical History

- The medical history must include all diagnoses identified in Appendix II (Maternal Endpoint Diagnoses). Any allergies to any medications and their formulations must be documented.
- Medical history including cardiovascular risk history and family myocardial infarction/sudden cardiac death history as well as smoking status and alcohol status will be collected.
- All viral loads results obtained during pregnancy will be collected.

6.2.3 Medication History

A medication history must be present, including start and stop dates, in the source documents and on the CRFs, including:

- Complete HIV-1 treatment history, including start and stop dates of any ARV medication (estimated if the exact dates cannot be obtained), and immune-based therapy, or HIV-related vaccines, including blinded study medications.
- All prescription medications taken within 30 days prior to study entry, including actual or estimated start and stop dates.
- The following nonprescription medications taken within 30 days prior to study entry: antacids, H2 blockers, proton pump inhibitors, aspirin, and non-steroidal anti-inflammatory drugs (NSAIDS).

In addition to the above, alternative/complementary therapies taken within 30 days prior to study entry should be recorded in source documents, as should allergies to any medications and their formulations.

6.2.4 Clinical Assessments

Complete Physical Exam
A complete physical examination at screening is to include at a minimum an examination of the skin, head, mouth, and neck; auscultation of the chest; cardiac exam; abdominal exam; and examination of the lower extremities for edema. The complete physical exam will also include signs and symptoms, diagnoses, and vital signs (temperature, pulse, respiration rate, and blood pressure); however, normal temperature, pulse, and respiration rate need not be recorded on the CRFs.

Targeted Physical Exam
A targeted physical examination is to include vital signs (temperature, pulse, respiration rate, and blood pressure) and is to be driven by any previously identified or new signs or symptoms including diagnoses that the participant has experienced within 30 days prior to screening/since the last visit. This examination will be performed according to the SOE and recorded on the CRFs (with the exception that normal temperature, pulse, and respiration rate need not be recorded on the CRFs).

Height
Collect height once at or prior to study entry.

Weight
Collect weight at all study visits.

Cardiovascular Risk History
Collect at study entry.

Smoking Status
Collect at study entry and then every 48 weeks.

Alcohol Status
Collect at study entry and then every 48 weeks.

Gynecologic Status
Collect at week 12, week 48 and then every 48 weeks.

Substance Use Self-Report
Collect at study entry and then every 48 weeks.

WHO Clinical Staging
Collect at every study visit.

Signs and Symptoms
At entry, all signs and symptoms of any Grade occurring ≤ 30 days before study entry must be recorded. Post-entry, only signs and symptoms Grade ≥ 3 must be recorded on the CRFs. All signs and symptoms that lead to a change in treatment, regardless of grade, must be recorded.

All Grade 4 signs and symptoms and signs and symptoms of any grade that lead to a change in treatment will be further evaluated and may require additional supporting information in order to assess for relationship to study drugs. The additional evaluation(s) must be recorded on the CRFs.

Sites must refer to the Division of AIDS Table for Grading the Severity of Adult and Pediatric Adverse Events, Version 1.0, December 2004 with Clarification dated August 2009, which can be found on the DAIDS RSC Web site: http://rsc.tech-res.com.

Diagnoses
At entry, all diagnoses identified by the Pediatric/Maternal Diagnoses criteria during the most recent pregnancy are to be recorded. After entry, all diagnoses identified by the Pediatric/Maternal Diagnoses criteria since the last study visit are to be recorded. The Pediatric/Maternal Diagnoses can be found in the appropriate appendix (as directed on the relevant diagnosis CRF) on the IMPAACT Data Management Center website: www.fstrf.org.

With the exception of WHO Stage 2 Clinical Events, the diagnoses identified in Appendix II (Maternal Endpoint Diagnoses) will be further evaluated and may require additional supporting information to assess the relationship to study drugs and for study endpoint verification. The additional evaluation(s) must be recorded on the appropriate CRF.

Concomitant Medications
Information on all medications/preparations received by participants since the last study visit (prescription and non-prescription medications/preparations and alternative/complementary therapies), including estimated start and stop dates, are to be recorded in the participants' source records, as this information will be needed for assessment of toxicities and adverse events; a subset of these — prescription medications and contraceptives — will also be recorded on applicable CRFs for entry into the study database.

Study Treatment Modifications
During the study, all modifications of study drug regimens, including participant-initiated and/or protocol-mandated interruptions, modifications, and permanent discontinuation of treatment will be recorded on the CRFs at each visit. Participant-initiated and protocol-mandated interruptions include both inadvertent and deliberate interruptions of study drugs for a period of > 7 consecutive days. The study CMC must be notified of the substitution or permanent discontinuation of any study drug.

6.2.5 QOL/Adherence/Resource Utilization Questionnaires

The Quality of Life (QOL)/Adherence/Resource Utilization questionnaires will be completed at the time points indicated on the SOE.

NOTE: Because the self-reported adherence assessment also includes components of quality of life, it will be performed even if participants prematurely discontinue therapy.

6.2.6 Laboratory Evaluations

Sites must refer to the Division of AIDS Table for Grading the Severity of Adult and Pediatric Adverse Events (DAIDS AE Grading Table), Version 1.0, December 2004 with Clarification dated August 2009, which can be found on the DAIDS RSC Web site: http://rsc.tech-res.com.

At screening and entry all laboratory values must be recorded. For post entry assessments, record all Grade ≥ 3 laboratory results. The only exceptions to this are serum creatinine (all grades of creatinine must be reported) and hematologic and hepatic abnormalities (all Grade ≥ 2 renal, hematologic and hepatic abnormalities must be recorded). All laboratory toxicities that lead to a change in treatment, regardless of grade, must be recorded.

All Grade 3 or higher creatinine, AST or ALT values, all other Grade 4 laboratory values, and any grade laboratory value that leads to a change in treatment will be further evaluated and may require additional supporting information to assess the relationship to study drugs. The additional evaluation(s) must be recorded on the appropriate CRF.

Hematology
Hemoglobin, white blood cell count (WBC), differential WBC, ANC, and platelet count will be performed in real time at the local laboratory

Urine
Dipstick for protein

Blood Chemistries
Blood urea nitrogen (BUN), creatinine, electrolytes (sodium, potassium, chloride, and CO_2/bicarbonate), and phosphate will be performed in real time at the local laboratory

Calculated Creatinine Clearance

Calculated CrCl is required as estimated by the Cockcroft-Gault equation (see Section 4.1.10). All grades of creatinine should be reported.

Liver Function Tests (LFTs)

Total bilirubin, indirect bilirubin, AST (SGOT), ALT (SGPT), and alkaline phosphatase will be performed in real time at the local laboratory

NOTE: If participant develops symptoms suggestive of hepatitis, perform LFTs as soon as possible (and prior to the next scheduled study visit).

Hepatitis B Serology

Documentation of hepatitis B surface antibody (HBsAb) status and hepatitis B surface antigen (HBsAg) status (if antibody status is negative) within 12 months prior to study entry is required per Inclusion Criterion 4.1.3. When required documentation is available, testing need not be repeated for the study. When required documentation is not available, testing should be performed as part of the screening process, with results available prior to study entry. Sites may choose to perform HBsAb testing first and then perform HBsAg testing only for those who test HBsAb negative. Alternatively, sites may test for HBsAg first; in this case, HBsAb testing is not required. Required results will be recorded on the CRFs.

Pregnancy Test

Pregnancy testing will be done when clinically indicated/suspected beginning at study week 4, with the exception that women receiving EFV will be tested for pregnancy at each study visit while on EFV and through 12 weeks after stopping EFV. Women of reproductive potential (who have not reached menopause, undergone hysterectomy, bilateral oophorectomy, or tubal ligation) will be tested with a serum or urine β-HCG pregnancy test (the urine test must have a sensitivity of at least 50 mIU/mL; urine pregnancy tests that are more sensitive than this, including those with lower detection limits at or below 20 mIU/mL, are acceptable). For management of pregnancy see Section 6.6.

Fasting Lipid Profile - Metabolic Studies

Total cholesterol, HDL cholesterol, LDL cholesterol (calculated), triglycerides, and glucose will be performed in real time at the local laboratory and all values will be recorded on the CRFs.

Participants will be fasting (nothing to eat or drink except water and required prescription medications for at least 8 hours). Sites are encouraged, but not required, to perform fasting labs in the morning to maximize participant compliance with the fasting requirement. If participants are in a non-fasting state, the metabolic panel labs will still be drawn and will be recorded on the CRFs, and the non-fasting state should be noted on the CRFs.

6.2.7 Immunologic Studies

CD4+/CD8+
Obtain absolute CD4+/CD8+ T-cell count and percentages within 45 days prior to entry from a CAP/CLIA-approved laboratory (for US sites) or a laboratory that operates according to GCLP guidelines, participates in appropriate external quality assurance programs, and is approved by the IMPAACT Central Laboratory (for non-US sites).

6.2.8 Virologic Studies

Plasma HIV-1 RNA
All HIV-1 RNA tests will be measured in real time using an FDA-approved HIV-1 RNA assay. HIV-1 RNA assays must be performed as indicated in the SOE by a CAP/CLIA-approved laboratory (for US sites) or a laboratory that operates according to GCLP guidelines, participates in appropriate external quality assurance programs, and is certified by the DAIDS VQA Program. Specimens will be processed and shipped according to the Lab Processing Chart (LPC). It is preferred, but not required that the same platform be used to measure each participant's plasma HIV-1 RNA throughout the study.

HIV-1 Resistance Testing Results (From Medical Records)
Previous HIV-1 resistance testing results verifiable by reports from a local CLIA-certified laboratory or equivalent any time prior to study entry should be collected at entry and included in the CRF. If more than one resistance testing result is available, only the most recent result prior to starting HAART is required, if available. Resistance testing results should be part of the source documentation.

HIV-1 Resistance Testing (Real Time)
When virologic failure is suspected, specimens for a confirmatory HIV-1 RNA and real-time resistance testing will be collected. The specimen collected at that visit will be sent to the Virology Specialty Laboratory (VSL) specified in the Manual of Procedures (MOP) for resistance testing once virologic failure has been confirmed. NRTI and PI resistance testing will be performed in real time and the results will be provided to the site.

6.2.9 Stored Samples

Stored Samples for Inflammatory/Thrombogenic Markers
Plasma will be collected and stored for later batch testing (laboratory to be determined) for high sensitivity C-reactive protein, interleukin-6, D-dimer and prothrombin fragments 1 and 2.

Stored Sample for HIV-1 Resistance Testing
HIV-1 resistance testing will be done retrospectively on a subset of stored samples. Results from HIV-1 resistance testing on blood specimens will be provided to the sites when available.

Stored plasma and DBS
Specimens will be collected, batched, and stored (see MOP and LPC) in the central specimen repository for future analyses and IMPAACT/ACTG-approved HIV-related research.

Cryopreserved PBMCs

Cryopreserved PBMCs will be collected from the first 200 women enrolled in Step 1 at sites that participate in the IQA cryopreservation proficiency testing program and that are able to guarantee long-term storage of the samples (> 4 weeks) in LN2 or at -150 C prior to shipment.

NOTE: All samples listed in the SOE should be collected from all participants, regardless of whether the participant provides informed consent for long-term (post-study) specimen storage and future use. These samples will be used for study-specific purposes as indicated above and as described in the ICF for screening and enrollment. After all study-specific testing has been performed, only specimens left over from participants who provided consent for long term storage and future use will be retained. Leftover specimens from participants who did not provide informed consent for long term storage and future use will be destroyed after all study-specific testing has been performed.

6.3 Management of HAART Regimens

6.3.1 Criteria for Switching HAART and Entering Step 3

Participants on Step 1, Arm A or Step 2 who meet one or more of the following criteria will be counseled on ARV treatment adherence and offered a switch to an alternate regimen. Participants who elect to switch regimens and otherwise meet eligibility criteria per Sections 4.5 and 4.6 will be enrolled in Step 3 (using the DMC Subject Enrollment System). Step 3 entry evaluations must be completed before the first dose of the second line regimen.

The criteria for switching to an alternate HAART regimen are:

- virologic failure (defined as two successive measurements of plasma HIV-1 RNA > 1000 copies/mL, with the first measurement taken at or after at least 24 weeks on HAART; see note below for more information on counting weeks on HAART) OR
- clinical failure (defined as development of a new or recurrent AIDS defining/WHO Stage 4 condition or any other clinical condition that is considered an indication for HAART by country-specific guidelines) OR
- immunologic failure (defined as a confirmed decrease in CD4+ cells to either <50% of the participant's Step 1 maximum or to <350 cells/mm^3 OR
- significant toxicity on HAART requiring a change in the backbone of the regimen or otherwise requiring a change in more than one class of study drug if approved by the CMC in advance OR
- country-specific standard indication for a complete change of HAART regimen OR
- otherwise requires a change to an alternate HAART regimen as determined in consultation with the CMC

NOTE: For purposes of defining virologic failure, the 24 weeks referenced above refers to the number of continuous weeks on a HAART regimen and includes time on a HAART regimen during pregnancy (prior to study entry) and after pregnancy outcome even if a different HAART regimen is taken after pregnancy outcome. See Section 6.4 for more information on monitoring and management of viral loads and please consult the CMC with any questions related to counting weeks on HAART and/or other aspects of defining failure.

NOTE: With regard to immunologic failure, CD4+ cell counts <50% of the Step 1 maximum or <350 cells/mm^3 should be confirmed with a second test ideally within 30 days and in all cases within 90 days. If the second test does not confirm the decrease, the test should be repeated again and the CMC should be consulted. If the third test confirms the decrease within 90 days of the first test, in consultation with the CMC, the decreased CD4+ cell count should be considered confirmed. The number of days between tests should be determined based on the date of specimen collection for each test.

Women in whom virologic, clinical, or immunologic failure is believed to be due to non-adherence, systemic illness, vaccination, or other circumstances determined by the study clinicians, will not be required to switch therapy and/or enter Step 3. The initial HAART regimen should be continued with laboratory evaluations as clinically indicated and as specified in the SOE.

Women who experience a drop in CD4 cell count despite ongoing viral suppression should be evaluated for other causes of immunologic failure such as inter-current illness, bone marrow disorders or use of concomitant medications that are marrow suppressive.

6.3.2 Criteria for Reinitiating (or Continuing) HAART and Entering into Step 2

Participants on Step 1, Arm B who meet one or more of the following indications for antiretroviral therapy will be counseled to reinitiate HAART; those who elect to do so, and otherwise meet eligibility criteria per Sections 4.3 and 4.4, will be enrolled in Step 2 (using the DMC Subject Enrollment System):

- develops an AIDS-defining/WHO Stage 4 illness OR
- has a confirmed CD4+ cell count <350 cells/mm^3 OR
- develops a clinical condition (other than pregnancy) that is considered an indication for HAART by country-specific guidelines OR
- otherwise requires HAART as determined in consultation with the CMC.

Participants on Step 1 Arm A who meet one or more of the above-listed criteria before meeting criteria to enter Step 3 and switch to a second line regimen will be counseled to continue HAART; those who elect to do so, and otherwise meet the eligibility criteria per Sections 4.3 and 4.4, will be enrolled in Step 2 (using the DMC Subject Enrollment System).

CD4+ cell counts < 350 cells/mm^3 should be confirmed with a second test ideally within 30 days and in all cases within 90 days. If a second test provides a CD4+ cell count of 350 or higher, the test should be repeated again and the CMC should be consulted. If the third test provides a CD4 cell count < 350 cells/mm^3 within 90 days of the first CD4+ cell count < 350 cells/mm^3, in consultation with the CMC, the CD4+ cell count should be considered confirmed. The number of days between tests should be determined based on the date of specimen collection for each test.

For participants entering Step 2 from Step 1, Arm B, Step 2 entry evaluations must be completed before the first dose of HAART. It is expected that the initial regimen when restarting HAART will be LPV/RTV and TDF/FTC, but an alternate regimen of study-supplied HAART may be used if resistance to or toxicity from these agents has occurred in the past.

6.4 Monitoring and Management of Viral Loads

6.4.1 Monitoring Viral Load

Step 1 - Arm A, Step 2 and Step 3
Virologic failure is not an endpoint in this trial, however, monitoring viral load is used to maximize the benefits of HAART and to determine when treatment should be changed and hence, virologic monitoring will be provided for all participants receiving HAART (those participants randomized to continue HAART (Step 1, Arm A), those who initiate HAART for their own health (Step 2) and those who change to a second line regimen (Step 3). For these women, HIV-1 RNA will be evaluated at baseline (entry into Step 1, Step 2 or Step 3), and at weeks 4 and 12 after entry and every 12 weeks thereafter (see Appendix I).

Note: For any women in Step 1 Arm A, Step 2, or Step 3 who stop HAART for any reason, virologic monitoring should continue per the study SOE.

Step 1 - Arm B
Women randomized to Arm B will have HIV-1 RNA evaluated at study entry, but will not have virologic monitoring on study until they re-initiate HAART for clinical indications. Study participants are allowed to have viral load monitoring done as standard of care outside of the study.

Note: For any women who start HAART in Step 1 Arm B, virologic monitoring is not required per the study SOE. However, virologic monitoring may be performed outside of the study if consistent with local standards of care. In this case, if study staff are able obtain documentation of such testing, the results should be recorded on study CRFs.

6.4.2 Definition of Virologic Failure

Virologic failure is defined as two successive measurements of plasma HIV-1 RNA >1,000 copies/mL, with the first measurement taken at or after at least 24 weeks on HAART. See note in Section 6.3.1 for more information on counting weeks on HAART.

6.4.3 Confirmation of Suspected Virologic Failure and Resistance Testing

Women receiving HAART, who have a plasma HIV-1 RNA level > 1,000 copies/mL at or after 24 weeks on initial or second-line therapy should return (if possible within 4 weeks) for confirmatory plasma HIV-1 RNA and for a plasma sample for real-time resistance testing.

The specimen collected will be sent to the laboratory designated in the MOP for resistance testing once the plasma HIV-1 RNA level has been confirmed to be > 1,000 copies/mL. Participants should continue their current regimen while resistance testing is being performed.

6.4.4 Management of Confirmed Virologic Failure

Women with confirmed virologic failure at or after 24 weeks of initial or second-line therapy are strongly encouraged to modify therapy. Study-provided medications will be available to these participants or participants may access therapy not provided by the study. Therapy choice should be guided by real-time resistance testing provided by the study and should meet the protocol definition of HAART.

In the event that a participant has confirmed virologic failure but does not wish to change her assigned regimen due to clinical and immunologic stability, she may, in consultation with her primary care provider, remain on her current HAART regimen and continue to be followed on study with clinical and laboratory monitoring. If either clinical or immunologic failure (defined in Section 6.3.1) occurs at any time, participants are strongly advised to enroll in Step 3 and switch therapy. The regimen should be selected by the primary care provider and/or site investigator using resistance testing data performed as close to the time of the change in therapy as available. Resistance testing at the time of confirmation of HIV-1 RNA > 1,000 copies/mL will be provided by the study.

6.4.5 Management of a Second (or Subsequent) Episode of Virologic Failure

Viral load and CD4+ cell counts will be monitored during follow-up on the second-line or subsequent regimen (see SOE). If confirmed virologic failure occurs at or after 24 weeks on a second-line or subsequent regimen and the study participant has maintained a CD4+ cell count > 350 cells the participant can decide with the study clinician whether to try to further modify the regimen with available HAART or to stop HAART. A switch to a third line regimen is not a requirement of the study. Participants who discontinue HAART will be followed off-therapy/on-study. Cases of second-line or subsequent- failure should be discussed with the study CMC.

Second-line failure due to non-adherence or intolerance may be managed with use of the study-provided medications and decisions will be made on a case by case basis. If the participant has never had CD4+ cell counts < 400 cells/mm^3 and is intolerant to available HAART, consideration may be given to careful observation off ART. Participants who discontinue HAART will be followed off-therapy/on-study.

6.5 Management of Antiretroviral Drug Resistance

The purpose of an alternative regimen is to provide a second treatment regimen for management of presumed and/or documented loss of potency due to the development of drug resistance with the ART used in initial therapy. All alternative regimens will consist of any potentially effective combination of three or more ARV drugs from two or more classes of ARV, either study-provided or not. Site investigators, according to expected patterns of resistance following failure, will construct alternate regimens. Initial regimens will contain the PI LPV/RTV and TDF/FTC. The CMC should be consulted as needed regarding second line regimen options.

It is expected that high-level 3TC and FTC resistance will occur during most initial regimen failures. However, because 3TC or FTC may provide continued benefit despite the presence of high-level resistance, 3TC or FTC will be allowed as a third NRTI in alternative regimens when potentially viable two-NRTI combinations are not available, at the discretion of the site investigator. In other words, 3TC or FTC would be a third NRTI (e.g., using TDF/ZDV/3TC + a PI or NNRTI (or other class)).

RTV must be used to boost ATV whenever ATV is used.

6.6 Management of Participants Who Become Pregnant On Study

Participants who become pregnant while on study-supplied study drug at all NIAID-funded and NICHD-funded sites must provide informed consent to continue study-supplied study drug during their pregnancy (Appendix VI).

Participants who become pregnant on Step 1, Arm A will continue a HAART regimen during pregnancy. They will be provided information and counseling on their current regimen and what is known about the safety of the ARVs they are currently taking during pregnancy. Participants on LPV-RTV may have a dose increase in the third trimester of pregnancy if consistent with local standards of care. Participants on tenofovir will be informed of the option to switch to zidovudine. Other components of each regimen will be evaluated on a case-by-case basis based on available data regarding use in pregnancy.

Participants on Step 1, Arm B who become pregnant off HAART will receive ARVs for PMTCT according to the local standard of care for pregnant women.

Participants on Step 2 or Step 3 who become pregnant while on HAART will be counseled the same as women on Step 1, Arm A.

Pregnancy outcomes should be ascertained and recorded on study CRFs. For participants who are pregnant at their Study Discontinuation visits, additional post-study contacts should be completed to ascertain pregnancy outcomes. Outcomes may be ascertained based on participant report but medical records should be obtained whenever possible to supplement participant reports.

Sites are encouraged to prospectively register pregnant participants in the Antiretroviral Pregnancy Registry (APR) by calling the following number in the US: +1-800-258-4263. Outside of the US, please see the APR website (www.apregistry.com) for additional toll-free numbers.

6.7 Management of Participants Who Develop Active Tuberculosis

Participants on Step 1 Arm B who develop TB should enter Step 2 and start HAART. Participants who develop TB and need rifampin-containing TB treatment while on a HAART regimen that includes LPV/RTV may be offered EFV in place of LPV/RTV if they can use appropriate contraceptives as outlined below. Women who do not want to use EFV can use a triple nucleoside combination while on rifampin. All participants on TB treatment may continue to receive FTC/TDF.

These study drug changes will be made available for the duration of the rifampin-based TB treatment, and for 30 days after stopping rifampin. Thereafter, the participant will return to her prior study drug regimen.

NOTE: Participants who are participating in sexual activity that could lead to pregnancy and who are receiving EFV must agree to use two reliable methods of contraception, including a reliable barrier method of contraception together with another reliable form of contraception while receiving EFV and for 12 weeks after stopping EFV. For such participants, pregnancy testing should be performed at every study visit while on EVF and for 12 weeks after stopping EFV.

6.8 Discontinuation of HBV-active ART in HBsAg+ Participants

HBsAg+ participants who discontinue HBV-active ART (including TDF, FTC, or 3TC) may be at risk of rebound HBV viremia and subsequent transaminitis. HIV/HBsAg+ participants who discontinue HBV-active ART as part of the protocol will have transaminases measured at 4 and 12 weeks after randomization. If, after HAART discontinuation, a woman experiences a ≥ Grade 3 transaminase elevation or is symptomatic (jaundice, severe fatigue), study clinicians should contact the study CMC regarding further management.

6.9 Toxicity Management

Toxicity management is described in Appendix III. The DAIDS Table for Grading the Severity of Adult and Pediatric Adverse Events, Version 1.0, dated December 2004 with Clarification dated August 2009, (which can be viewed at the following website: http://rsc.tech-res.com) must be followed. The CMC should be informed and consulted as needed or required.

6.10 Criteria for Treatment Discontinuation

Study drugs may be discontinued for any of the following reasons:

- Drug-related toxicity (see Appendix III)
- Requirement for prohibited concomitant medications
- Failure by the participant to attend 3 consecutive clinic visits
- Participant repeatedly noncompliant with study treatment as prescribed, as determined by the site investigator
- Clinical reasons believed life threatening by the site investigator, even if not addressed in the toxicity management of the protocol
- Request of the primary care provider if s/he thinks the study treatment is no longer in the best interest of the participant
- Request by the participant.

NOTE: Early discontinuation of study drugs is not a reason for study discontinuation; women who discontinue study drugs early will remain on study.

6.11 Criteria for Study Discontinuation

Participants will be discontinued from the study for any of the following reasons:

- Request by the participant to withdraw
- Request of the primary care provider if s/he thinks the study is no longer in the best interest of the participant
- Participant judged by the investigator to be at significant risk of failing to comply with the provisions of the protocol as to cause harm to self or seriously interfere with the validity of the study results
- At the discretion of the IMPAACT, ACTG, NIAID, NICHD, IRB or EC, Office for Human Research Protections (OHRP), local ministry of health or other required country-specific review boards, investigator, or pharmaceutical supporters
- Imprisonment or involuntary confinement in a medical facility (e.g., for psychiatric illness or infectious disease).

7.0 ADVERSE EVENT REPORTING TO DAIDS

Requirements, definitions and methods for expedited reporting of adverse events (AEs) are outlined in Version 2.0 of the Manual for Expedited Reporting of Adverse Events to DAIDS (DAIDS EAE Manual), which is available on the Regulatory Support Center (RSC) website: http://rsc.tech-res.com/safetyandpharmacovigilance.

The internet-based DAIDS Adverse Experience Reporting System (DAERS) must be used for expedited AE reporting to DAIDS. In the event of system outages or technical difficulties, expedited AEs may be reported via the DAIDS EAE Form. For questions about DAERS, please email DAIDS-ESSupport@niaid.nih.gov. Queries may also be sent from within the DAERS application itself.

7.1 Reporting Requirements

The “SUSAR” expedited AE reporting category, as defined in Version 2.0 of the DAIDS EAE Manual, will be used for this study. According to this category, all serious and unexpected AEs that are considered related to study-supplied study drug will be reported as EAEs. The definitions of the terms serious, unexpected, and related provided in the DAIDS EAE Manual will be used.

The study drugs for which expedited AE reporting is required are study-supplied atazanavir (ATV), didanosine (ddI), emtricitabine/tenofovir (FTC/TDF), emtricitabine/tenofovir/rilpivarine (FTC/TDF/RPV), lamivudine (3TC), lamivudine/zidovudine (3TC/ZDV), lopinavir/ritonavir (LPV/RTV), raltegravir (RAL), ritonavir (RTV), tenofovir (TDF), and zidovudine (ZDV).

In addition to the SUSAR EAE Reporting Category identified above, all fetal deaths occurring at or after 20 weeks gestation in new pregnancies must be reported as EAEs regardless of relatedness or expectedness.

7.2 Severity Grading

The most current version of the DAIDS Table for Grading the Severity of Adult and Pediatric Adverse Events will be used to grade the severity of EAEs. The table is available on the RSC website: http://rsc.tech-res.com/safetyandpharmacovigilance.

7.3 Expedited AE Reporting Period

The expedited AE reporting period for this study is from when a participant starts (is prescribed) one or more study-supplied study drugs until study completion or discontinuation of the participant from study participation for any reason.

After the above-described EAE reporting period, only SUSARs as defined in Version 2.0 of the DAIDS EAE Manual will be reported to DAIDS if study staff become aware of the events on a passive basis (from publicly available information).

8.0 STATISTICAL CONSIDERATIONS

8.1 General Design Issues

This randomized strategy trial addresses therapeutic questions for women from regions where antepartum HAART for PMTCT (for all CD4+ cell counts) and postpartum formula feeding is standard of care, and who also have both a pre-HAART CD4+ cell count $\geq$ 400 cells/mm^3 and a screening (on-HAART) CD4+ cell count $\geq$ 400 cells/mm^3. For these women, the objectives relate to the relative efficacy and safety of continuing HAART (when it is no longer used for PMTCT) versus discontinuing HAART.

8.2 Outcome Measures

The qualifying illnesses and conditions corresponding to the primary and secondary endpoints specified below are listed in Appendix II. Definitions of terms used below are as follows:

- "AIDS-defining illness" refers to the WHO Clinical Stage 4 illnesses listed in Appendix II
- "HIV/AIDS-related event" refers to the WHO Clinical Stage 4 illnesses, pulmonary tuberculosis, and other serious bacterial infections listed in Appendix II
- "Other metabolic events" refers to diabetes mellitus, lipodystrophy, and dyslipidemia as defined in Appendix II
- WHO Clinical Stage 2 and 3, cardiovascular, hepatic, and renal events, and other targeted medical conditions are listed in Appendix II

8.2.1 Primary Endpoint:

Composite endpoint of AIDS-defining illness, serious non-AIDS-defining cardiovascular, renal, or hepatic event, or death

8.2.2 Secondary Endpoints:

- Each component of the composite primary endpoint
- HIV/AIDS-related events
- Cardiovascular or other metabolic events
- Other targeted medical conditions
- Composite endpoint of HIV/AIDS-related event or death
- Composite endpoint of HIV/AIDS-related event or WHO Clinical Stage 2 or 3 event
- Composite endpoint of any condition outlined in Appendix II or death
- Selected Grade 2 laboratory abnormalities and all Grade 3 or higher laboratory values and signs and symptoms
- HIV-1 viral resistance to ART at 1, 2, and 3 years after randomization and at end of study
- Self reported adherence to HAART
- Quality of life measurements at 1, 2, and 3 years following randomization
- Plasma concentrations of inflammatory and thrombogenic markers
- Cost-Effectiveness

8.3 Randomization and Stratification

Women will be randomized in a 1:1 ratio to continue versus discontinue HAART, with balancing by site. The randomization will be stratified according to screening CD4+ count 400-499 versus 500-749 versus ≥ 750 cells/mm^3 (obtained while on HAART and within 45 days prior to study entry) to ensure balance with respect to screening CD4+ cell count in the two IMPAACT 1077HS randomized arms. The 500 cells/mm^3 cut-off was chosen because it is used in the US antiretroviral treatment guidelines (see Section 1.1). The 750 cells/mm^3 cut-off was chosen based on unpublished data from IMPAACT P1025 which suggest that among the women in the IMPAACT 1077HS population with screening CD4+ cell counts > 500 cells/mm^3, approximately equal proportions of women would have screening CD4+ cell counts of 500-749 cells/mm^3 and ≥ 750 cells/mm^3.

8.4 Sample Size and Accrual

There are very few empirical results on the long-term risk of AIDS/death in HIV-infected women from developed countries with high initial CD4+ cell counts (400-1,000 cells/mm^3), who will initiate HAART regimens at current CD4+ ‘triggers’ (typically ≤ 350 CD4+ cells). As a result, we used stochastic statistical models for patterns of CD4+ decline, effects of HAART initiation when CD4+ cell counts fall < 350 cells, and the association between current CD4+ cell count and AIDS/death to estimate the cumulative incidence of AIDS or death over time in women who discontinue HAART at delivery (or other pregnancy outcome). Using one set of models for the heterogeneity (across women) and rates of decline in CD4+ cell count over time, and the changing risk of opportunistic infection (OI)/death with changing CD4+ cell count, we estimate that about 4.6% of women randomized to discontinue HAART would have an AIDS/death event if followed for 3 years, corresponding to an average annual progression rate of 1.533%. From the SMART study, expanding the composite endpoint of AIDS progression or death to include serious non-AIDS events increased the event rate by 35% (27 events versus 20). Assuming that the same proportion would apply in 1077HS, we estimate that the annualized rate of the composite outcome of serious non-AIDS event or OI or death in 1077HS would be 2.07%. With an evaluable sample size of 1,700 women enrolled over 4 years, and an additional 1.75 years of follow-up of all women after the last one is randomized (average follow-up of 3.75 years), there would be 90% power to detect a 50% reduction in the rate of the composite endpoint of death, OI, or serious non-AIDS event in the group who continue HAART, based on a 2-sided Type I error of 5%. Adjusting this sample size by 3% for interim monitoring and a cumulative loss to follow-up rate of 14%, a total accrual of 2,000 women enrolled over 4 years, with an additional follow-up of 1.75 years, would provide the desired power. Because of the uncertainty in the estimated control group event rate on which these calculations are based, we also considered the predicted control group rate using a more sophisticated model that allows a biphasic effect of HAART discontinuation on CD4+ cell count. This model led to estimated rates about 50% greater than those in the first model; for example, an annualized rate for the composite endpoint of about 3% per year. This would lead to shorter or smaller trial than that based on the initial estimates. For example, with 4 years of accrual (same sample size) only an additional .57 years of follow-up would be needed. Alternatively, using the same study duration of 5.75 years, only about 2.3 years of accrual (1,150 women) would be needed. In both scenarios, a total of about 48 participants in the control arm (Arm B) are expected to experience the primary endpoint during the course of the study.

8.5 Monitoring

The core protocol team will review the status of the study regularly. This review will examine reports on accrual, characteristics of participants, retention, data and specimen completeness and adverse events. These reports will present overall results that are pooled across all randomized groups and not broken out according to groups. A full monitoring plan with specific details concerning the content and scheduling of these reports will be disseminated in a separate document before the study opens to accrual.

Accrual to this study will be monitored by the IMPAACT leadership in accordance with standard operating procedures. In addition, the team will monitor feasibility quarterly, first based on site protocol registration and then on accrual. Initially, the team will monitor site protocol registration quarterly to ensure that an adequate number of sites have registered to complete the protocol. If fewer than one-half of eligible IMPAACT sites

have registered after the protocol has been approved for 6 months, the team will re-assess the feasibility of the protocol and the reasons why sites have not registered, and will possibly amend the protocol accordingly. Once one-half of eligible IMPAACT sites have registered, the team will assess accrual on a quarterly basis. If fewer than 500 women have enrolled within 12 months after one-half of all eligible IMPAACT sites have opened to enrollment, the team will identify the reasons for lack of accrual and possibly amend the protocol accordingly.

The team will regularly monitor two types of treatment non-adherence at the site level: the proportion of women randomized to continue HAART who prematurely discontinue HAART, and the proportion of women randomized to discontinue HAART who actually reinitiate HAART prior to meeting the protocol-specified criteria for reinitiating HAART (Section 6.3.2). The study norms are that both proportions should be no greater than 10%. Discussions and remedial actions will be developed for any site that fails to meet either norm.

The study Toxicity Review Group, composed of the protocol chair and/or other designees, the NIAID and NICHD Medical Officers, the protocol statisticians, and data managers will review toxicity data at least monthly.

The study will also be reviewed by an NIAID-sponsored Data Safety and Monitoring Board (DSMB). Interim analyses of safety, study logistics, and an assessment of the accuracy of the assumed annualized rate (2.07%) of primary endpoints in the control (discontinue HAART) group will be undertaken annually after the first participant is enrolled. If the actual accrual and/or control group event rate(s) differ from the assumed rate(s), the sample size/trial duration will be modified accordingly. In addition, CD4+ cell counts at the time of randomization as well as adherence to the criteria for reinitiating HAART (Section 6.3.2) will be monitored. If the percentage of women with 400-500 CD4+ cell counts at randomization is large, it could lead to an insufficiently short time to re-initiation of HAART in the women randomized to discontinue HAART, thus diminishing the power of the study to detect a difference between the study arms. Women randomized to discontinue HAART will also be monitored for adherence, as described above, in order to determine whether they are re-initiating HAART according to the protocol. The reported adherence rates and norms described above, as well as the overall and site-specific adherence rates, will be included in each closed DSMB report (both pooled and by study arm). In the open DSMB report, the pooled results will be presented. These results will be discussed with the PROMISE team.

Under the assumed accrual rate and an additional 1.75 years of follow-up of all participants after the last is enrolled, approximately 16% of the expected number of primary endpoints in the control group would be expected to occur by 3 years after the first participant is enrolled, 35% by 4 years after the first participant is enrolled, and 65% by 5 years after the first participant is enrolled.

Interim analyses of efficacy will be undertaken annually once at least 25% of the expected number of primary endpoints in the control group have occurred, which is anticipated approximately 4 and 5 years after the first participant is enrolled into the study. The interim efficacy analysis schedule may be modified if accrual assumptions turn out to be inaccurate or if recommended by the DSMB. The Lan-DeMets approach and an O'Brien-Fleming spending function will be used. Because a transient benefit in the "continue HAART" arm might not be considered sufficient to change practice, consideration of stopping the trial for demonstration of benefit in the "continue HAART"

arm would be based on both a significant p-value and, by visual inspection of the estimated time-to-event distributions (see below), a lack of evidence that the observed effect is transient. In addition, consideration would be given to the consistency between effects seen on the primary endpoint and those seen in the secondary endpoints. Except for the endpoint of death from any cause, a significant difference between the “continue HAART” versus “discontinue HAART” arm with respect to a secondary endpoint, in the absence of strong evidence of a difference in the primary endpoint, would not be grounds for stopping the trial. On the other hand, strong evidence of a difference in the primary endpoint favoring one arm, but with evidence favoring the other arm with an important secondary endpoint, might support the continuation of the trial.

8.6 Analyses

Full details of the proposed analyses will be described in a statistical analysis plan that will be developed once enrollment to the study begins and prior to the commencement of analyses for the first review by the DSMB. Hence, here we limit the description of the proposed analyses to those for the primary efficacy endpoint.

Analyses will use the principle of intention-to-treat and will include all women randomized. Comparisons between treatment groups will be based on log rank tests for testing and Cox regression models for estimation of treatment effect sizes. In light of the conservative spending function that will be used in interim efficacy analyses, unadjusted point estimates, p-values, and 95% confidence intervals will be used to summarize the results in the final analysis. The distributions of time until events will be summarized using Kaplan-Meier estimators. Secondary efficacy endpoints will be analyzed similarly.

The analysis would become complicated if, during the conduct of PROMISE, the country-specific CD4+ cell count threshold for initiating HAART were to increase in one or more countries; for concreteness, the following discussion will use an increase in the threshold from 350 to 500 cells/mm^3 as an example, but similar considerations would apply to other threshold increases. Such a change could have two effects: (1) women in those countries with a screening CD4+ cell count between 400 and 500 cells/mm^3 may not be enrolled even though they are eligible according to the protocol, which may, on average, result in a healthier study population with lower event rates in those countries; and, (2) women in those countries who are randomized to Arm B (discontinue HAART) may restart HAART when their CD4+ cell count is above 350 cells/mm^3, which would make the strategies of continuing versus discontinuing HAART more similar. If such a change were to occur in all countries early during the PROMISE trial, the interpretation of the arms would be clear, but there might not be adequate power to detect a difference. On the other hand, if the change were to occur in all countries mid-way through the trial, the comparator arm to continue HAART would become harder to interpret because two policies for reinitiating HAART would contribute to the results. As discussed in section 8.5, interim analyses for DSMB review will include analyses of screening CD4+ cell count distributions, the accuracy of the assumed annualized rate (2.07%) of primary endpoints in the control (discontinue HAART) group, and adherence to the criteria for reinitiating HAART (Section 6.3.2) so that the DSMB may monitor these issues and recommend study modifications if needed.

If the CD4+ threshold for initiating HAART were to increase in some countries but not in others, then event rates and possibly treatment effects may also differ between countries. If this were to happen, such that the treatment effect differed significantly by country, then analyses would be performed to determine the effect of CD4+ threshold on

the treatment effect. The results of these analyses would be presented, in addition to presenting the overall treatment effect. However, it may not be possible to disentangle the relative impact of country and CD4+ cell count threshold on the variability in treatment effects without very strong statistical assumptions, since some women with screening CD4+ cell counts between 400 and 500 cells/mm^3 may not be enrolled, and the exclusion of these women may change over time and by country. Sensitivity analyses will be conducted to assess the potential impact of these issues on the conclusions of the study. For example, the sensitivity analyses may include and exclude the countries with higher CD4+ cell count thresholds, or include and exclude all women with screening CD4+ cell counts between 400 and 500 cells/mm^3 (note that the stratification of the randomization according to screening CD4+ cell count will ensure that approximately equal numbers of women screening CD4+cell counts above 500 cells/mm^3 will be randomized to each strategy arm).

9.0 HUMAN SUBJECTS CONSIDERATIONS

9.1 IRBs/ECs and Informed Consent

This protocol, site-specific ICFs based on the sample forms in Appendices IV, V, and VI, and any subsequent modifications must be reviewed and approved by the IRBs/ECs responsible for oversight of the study. Written informed consent must be obtained from the participant. The ICF for screening and enrollment (Appendix IV) describes the purpose of the study, the procedures to be followed, and the risks and benefits of participation. Consent for screening and enrollment must be obtained before any study-specific procedures are performed. In addition, all women who enroll in the study will also be asked to document their willingness or unwillingness for post-study storage and future use of leftover specimens (Appendix V). Women at all NICHD-funded and NIAID-funded study sites who become pregnant while on study-supplied study drug will be asked to provide informed consent for continuation of study-supplied study drug (Appendix VI). A copy of the ICFs will be offered to the participant.

NOTE: Appendix VI does not request permission for additional specimen collection. All protocol-specified specimens should be collected from all participants per the SOE, regardless of whether the participant provides informed consent for post-study specimen storage and future use. At the end of the study, only specimens left over from participants who provided consent for long term storage and future use will be retained. Leftover specimens from participants who did not provide informed consent for long term storage and future use will be destroyed after all protocol-specified testing has been completed.

9.2 Participant Confidentiality

All laboratory specimens, evaluation forms, reports, and other records that are transferred or transmitted off-site for processing will be identified only by a coded number to maintain participant confidentiality. The use of participant identifiers on study records must comply with DAIDS policies on Requirements for Source Documentation and Essential Documents at Clinical Research Sites Conducting DAIDS Funded and/or Sponsored Clinical Trials. All records will be kept in a secured area. All computer entry and networking programs will be done with coded numbers only. Clinical information will not be released without written permission of the participant, except as necessary for monitoring by the Office for Human Research Protections (OHRP), NIAID, NICHD, IMPAACT, ACTG, site IRBs/ECs, other required country-specific review boards, and/or the pharmaceutical supporters.

9.3 Study Discontinuation

The study may be discontinued at any time by NIAID, NICHD, OHRP, IMPAACT, site IRBs/ECs, other required country-specific review boards, the pharmaceutical supporters, and/or other governmental agencies as part of their duties to ensure that research participants are protected.

10.0 PUBLICATION OF RESEARCH FINDINGS

Publication of the results of this trial will be governed by IMPAACT policies. Any presentation, abstract, or manuscript will be made available for review by the pharmaceutical sponsors prior to submission.

11.0 BIOHAZARD CONTAINMENT

As the transmission of HIV and other blood borne pathogens can occur through contact with contaminated needles, blood, and blood products, appropriate blood and secretion precautions will be employed by all personnel in the drawing of blood and shipping and handling of all specimens for this study, as currently recommended by the Centers for Disease Control and Prevention.

All infectious specimens will be sent using the ISS-1 SAF-T-PAK mandated by the International Air Transport Association Dangerous Goods Regulations-Packing Instruction 602. Refer to individual carrier guidelines (e.g., Federal Express or Airborne) for specific instructions.

12.0 REFERENCES

(1) Bardeguez AD, Shapiro DE, Mofenson LM et al. Effect of cessation of zidovudine prophylaxis to reduce vertical transmission on maternal HIV disease progression and survival. J Acquir Immune Defic Syndr 2003; 32(2):170-181.

(2) Watts DH, Lambert J, Stiehm ER et al. Progression of HIV disease among women following delivery. J Acquir Immune Defic Syndr 2003; 33(5):585-593.

(3) Krolewiecki AJ, Zala C, Vanzulli C et al. Safe treatment interruptions in patients with nadir CD4 counts of more than 350 cells/microL: a randomized trial. J Acquir Immune Defic Syndr 2006; 41(4):425-429.

(4) Ananworanich J, Gayet-Ageron A, Le BM et al. CD4-guided scheduled treatment interruptions compared with continuous therapy for patients infected with HIV-1: results of the Staccato randomised trial. Lancet 2006; 368(9534):459-465.

(5) Marchou B, Tangre P, Charreau I et al. Structured treatment interruptions in HIV-infected patients with high CD4 cell counts and virologic suppression: results of a prospective, randomized, open-label trial (Window - ANRS 106). 13th Conference on Retroviruses and Opportunistic Infections, Denver, Colorado Abstract 104. 2-7-2006.

(6) Danel C, Moh R, Sorho S et al. The CD4-guided strategy arm stopped in a randomized structured treatment interruption trial in West-African adults: ANRS 1269 Trivacan trial. 13th Conference on Retroviruses and Opportunistic Infections, Denver, Colorado Abstract 105LB. 2-7-2006.

(7) Emery S, Neuhaus JA, Phillips AN et al. Major clinical outcomes in antiretroviral therapy (ART)-naive participants and in those not receiving ART at baseline in the SMART study. J Infect Dis 2008; 197(8):1133-1144.

(8) El-Sadr W, SMART Study Group. Re-initiation of ART in the CD4-guided ART interruption group in the SMART study lowers risk of opportunistic disease or death. Presented at the 15th Conference on Retroviruses and Opportunistic Infections, February, 2008, Boston, MA. 2008.

(9) Egger M, May M, Chene G et al. Prognosis of HIV-1-infected patients starting highly active antiretroviral therapy: a collaborative analysis of prospective studies. Lancet 2002; 360(9327):119-129.

(10) May M, Sterne JA, Sabin C et al. Prognosis of HIV-1-infected patients up to 5 years after initiation of HAART: collaborative analysis of prospective studies. AIDS 2007; 21(9):1185-1197.

(11) Gras L, Kesselring AM, Griffin JT et al. CD4 cell counts of 800 cells/mm^3 or greater after 7 years of highly active antiretroviral therapy are feasible in most patients starting with 350 cells/mm^3 or greater. J Acquir Immune Defic Syndr 2007; 45(2):183-192.

(12) Moore RD, Keruly JC. CD4+ cell count 6 years after commencement of highly active antiretroviral therapy in persons with sustained virologic suppression. Clin Infect Dis 2007; 44(3):441-446.

(13) Buchbinder SP, Katz MH, Hessol NA et al. Herpes zoster and human immunodeficiency virus infection. J Infect Dis 1992; 166(5):1153-1156.

(14) Rogues AM, Dupon M, Ladner J, Ragnaud JM, Pellegrin JL, Dabis F. Herpes zoster and human immunodeficiency virus infection: a cohort study of 101 coinfected patients. Groupe d'Epidemiologie clinique du SIDA en aquitaine. J Infect Dis 1993; 168(1):245.

(15) Veenstra J, Krol A, van Praag RM et al. Herpes zoster, immunological deterioration and disease progression in HIV-1 infection. AIDS 1995; 9(10):1153-1158.

(16) Buchbinder SP, Katz MH, Hessol NA et al. Herpes zoster and human immunodeficiency virus infection. J Infect Dis 1992; 166(5):1153-1156.

(17) Donahue JG, Choo PW, Manson JE, Platt R. The incidence of herpes zoster. Arch Intern Med 1995; 155(15):1605-1609.

(18) Glesby MJ, Moore RD, Chaisson RE. Clinical spectrum of herpes zoster in adults infected with human immunodeficiency virus. Clin Infect Dis 1995; 21(2):370-375.

(19) Hoppenjans WB, Bibler MR, Orme RL, Solinger AM. Prolonged cutaneous herpes zoster in acquired immunodeficiency syndrome. Arch Dermatol 1990; 126(8):1048-1050.

(20) Jacobson MA, Berger TG, Fikrig S et al. Acyclovir-resistant varicella zoster virus infection after chronic oral acyclovir therapy in patients with the acquired immunodeficiency syndrome (AIDS). Ann Intern Med 1990; 112(3):187-191.

(21) McCutchan JA, Wu JW, Robertson K et al. HIV suppression by HAART preserves cognitive function in advanced, immune-reconstituted AIDS patients. AIDS 2007; 21(9):1109-1117.

(22) Porco TC, Martin JN, Page-Shafer KA et al. Decline in HIV infectivity following the introduction of highly active antiretroviral therapy. AIDS 2004; 18(1):81-88.

(22A) Cohen MS, Chen YQ, McCauley M, et al. Prevention of HIV-1 infection with early antiretroviral therapy. N Engl J Med 2011; 365:493-505.

(22B)Grinsztein B et al. Effect of early versus delayed initiation of antiretroviral therapy on clinical outcomes in the HPTN 052 randomized clinical trial. Oral abstract THLBB05, XIX International AIDS Conference, July 2012.

(22C) World Health Organization. Guidance on couples HIV testing and counselling including antiretroviral therapy for treatment and prevention in serodiscordant couples: recommendations for a public health approach. April 2012.

(23) Freedberg KA, Scharfstein JA, Seage GR, III et al. The cost-effectiveness of preventing AIDS-related opportunistic infections. JAMA 1998; 279(2):130-136.

(24) Walensky RP, Weinstein MC, Smith HE, Freedberg KA, Paltiel AD. Optimal allocation of testing dollars: the example of HIV counseling, testing, and referral. Med Decis Making 2005; 25(3):321-329.

(25) Paltiel AD, Walensky RP, Schackman BR et al. Expanded HIV screening in the United States: effect on clinical outcomes, HIV transmission, and costs. Ann Intern Med 2006; 145(11):797-806.

(26) Walensky RP, Weinstein MC, Yazdanpanah Y et al. HIV drug resistance surveillance for prioritizing treatment in resource-limited settings. AIDS 2007; 21(8):973-982.

(27) Sax PE, Islam R, Walensky RP et al. Should resistance testing be performed for treatment-naive HIV-infected patients? A cost-effectiveness analysis. Clin Infect Dis 2005; 41(9):1316-1323.

(28) Freedberg KA, Hirschhorn LR, Schackman BR et al. Cost-effectiveness of an intervention to improve adherence to antiretroviral therapy in HIV-infected patients. J Acquir Immune Defic Syndr 2006; 43 Suppl 1:S113-S118.

(29) Schackman BR, Gebo KA, Walensky RP et al. The lifetime cost of current human immunodeficiency virus care in the United States. Med Care 2006; 44(11):990-997.

(30) Walensky RP, Paltiel AD, Losina E et al. The survival benefits of AIDS treatment in the United States. J Infect Dis 2006; 194(1):11-19.

(31) Sax PE, Losina E, Weinstein MC et al. Cost-effectiveness of enfuvirtide in treatment-experienced patients with advanced HIV disease. J Acquir Immune Defic Syndr 2005; 39(1):69-77.

(32) Walensky RP, Goldie SJ, Sax PE et al. Treatment for primary HIV infection: projecting outcomes of immediate, interrupted, or delayed therapy. J Acquir Immune Defic Syndr 2002; 31(1):27-37.

(33) Freedberg KA, Losina E, Weinstein MC et al. The cost effectiveness of combination antiretroviral therapy for HIV disease. N Engl J Med 2001; 344(11):824-831.

(34) Goldie SJ, Yazdanpanah Y, Losina E et al. Cost-effectiveness of HIV treatment in resource-poor settings--the case of Cote d'Ivoire. N Engl J Med 2006; 355(11):1141-1153.

(35) Yazdanpanah Y, Vray M, Meynard J et al. The long-term benefits of genotypic resistance testing in patients with extensive prior antiretroviral therapy: a model-based approach. HIV Med 2007; 8(7):439-450.

(36) Schackman BR, Scott CA, Sax PE et al. Potential risks and benefits of HIV treatment simplification: a simulation model of a proposed clinical trial. Clin Infect Dis 2007; 45(8):1062-1070.

(37) Sax PE, Islam R, Walensky RP et al. Should resistance testing be performed for treatment-naive HIV-infected patients? A cost-effectiveness analysis. Clin Infect Dis 2005; 41(9):1316-1323.

(38) Goldie SJ, Yazdanpanah Y, Losina E et al. Cost-effectiveness of HIV treatment in resource-poor settings--the case of Cote d'Ivoire. N Engl J Med 2006; 355(11):1141-1153.

(39) Paltiel AD, Weinstein MC, Kimmel AD et al. Expanded screening for HIV in the United States--an analysis of cost-effectiveness. N Engl J Med 2005; 352(6):586-595.

(40) Walensky RP, Wood R, Weinstein MC et al. Scaling up antiretroviral therapy in South Africa: the impact of speed on survival. J Infect Dis. In press.

(41) Paltiel AD, Goldie SJ, Losina E et al. Preevaluation of clinical trial data: the case of preemptive cytomegalovirus therapy in patients with human immunodeficiency virus. Clin Infect Dis 2001; 32(5):783-793.

(42) Watts DH, Lambert J, Stiehm ER et al. Progression of HIV disease among women following delivery. J Acquir Immune Defic Syndr 2003; 33(5):585-593.

(43) Melvin AJ, Burchett SK, Watts DH et al. Effect of pregnancy and zidovudine therapy on viral load in HIV-1-infected women. J Acquir Immune Defic Syndr Hum Retrovirol 1997; 14(3):232-236.

(44) Cao Y, Krogstad P, Korber BT et al. Maternal HIV-1 viral load and vertical transmission of infection: the Ariel Project for the prevention of HIV transmission from mother to infant. Nat Med 1997; 3(5):549-552.

(45) Burns DN, Nourjah P, Wright DJ et al. Changes in immune activation markers during pregnancy and postpartum. J Reprod Immunol 1999; 42(2):147-165.

(46) Mikyas Y, Aziz N, Harawa N et al. Immunologic activation during pregnancy: serial measurement of lymphocyte phenotype and serum activation molecules in HIV-infected and uninfected women. J Reprod Immunol 1997; 33(2):157-170.

(47) Epiney M, Boehlen F, Boulvain M et al. D-dimer levels during delivery and the postpartum. J Thromb Haemost 2005; 3(2):268-271.

(48) Kuller LH, Tracy R, Belloso W et al. Inflammatory and coagulation biomarkers and mortality in patients with HIV infection. PLoS Med 2008; 5(10):e203.

(49) Aandahl EM, Michaelsson J, Moretto WJ, Hecht FM, Nixon DF. Human CD4+ CD25+ regulatory T cells control T-cell responses to human immunodeficiency virus and cytomegalovirus antigens. J Virol 2004; 78(5):2454-2459.

(50) Andersson J, Boasso A, Nilsson J et al. The prevalence of regulatory T cells in lymphoid tissue is correlated with viral load in HIV-infected patients. J Immunol 2005; 174(6):3143-3147.

(51) Eggena MP, Barugahare B, Jones N et al. Depletion of regulatory T cells in HIV infection is associated with immune activation. J Immunol 2005; 174(7):4407-4414.

(52) Tsunemi S, Iwasaki T, Imado T et al. Relationship of CD4+CD25+ regulatory T cells to immune status in HIV-infected patients. AIDS 2005; 19(9):879-886.

(53) Jonuleit H, Schmitt E. The regulatory T cell family: distinct subsets and their interrelations. J Immunol 2003; 171(12):6323-6327.

(54) Allan SE, Passerini L, Bacchetta R et al. The role of 2 FOXP3 isoforms in the generation of human CD4+ Tregs. J Clin Invest 2005; 115(11):3276-3284.

(55) Fantini MC, Becker C, Monteleone G, Pallone F, Galle PR, Neurath MF. Cutting edge: TGF-beta induces a regulatory phenotype in CD4+. J Immunol 2004; 172(9):5149-5153.

(56) Fontenot JD, Rudensky AY. A well adapted regulatory contrivance: regulatory T cell development and the forkhead family transcription factor Foxp3. Nat Immunol 2005; 6(4):331-337.

(57) Gavin MA, Torgerson TR, Houston E et al. Single-cell analysis of normal and FOXP3-mutant human T cells: FOXP3 expression without regulatory T cell development. Proc Natl Acad Sci U S A 2006; 103(17):6659-6664.

(58) Levings MK, Gregori S, Tresoldi E, Cazzaniga S, Bonini C, Roncarolo MG. Differentiation of Tr1 cells by immature dendritic cells requires IL-10 but not CD25+CD4+ Tr cells. Blood 2005; 105(3):1162-1169.

(59) Myers L, Croft M, Kwon BS, Mittler RS, Vella AT. Peptide-specific CD8 T regulatory cells use IFN-gamma to elaborate TGF-beta-based suppression. J Immunol 2005; 174(12):7625-7632.

(60) Hansen W, Loser K, Westendorf AM et al. G protein-coupled receptor 83 overexpression in naive CD4+. J Immunol 2006; 177(1):209-215.

(61) Liu W, Putnam AL, Xu-Yu Z et al. CD127 expression inversely correlates with FoxP3 and suppressive function of human CD4+ T reg cells. J Exp Med 2006; 203(7):1701-1711.

(62) Seddiki N, Santner-Nanan B, Martinson J et al. Expression of interleukin (IL)-2 and IL-7 receptors discriminates between human regulatory and activated T cells. J Exp Med 2006; 203(7):1693-1700.

(63) Sugimoto N, Oida T, Hirota K et al. Foxp3-dependent and -independent molecules specific for CD25+CD4+ natural regulatory T cells revealed by DNA microarray analysis. Int Immunol 2006; 18(8):1197-1209.

(64) Endharti AT, Rifa'I M, Shi Z et al. Cutting edge: CD8+CD122+ regulatory T cells produce IL-10 to suppress IFN-gamma production and proliferation of CD8+ T cells. J Immunol 2005; 175(11):7093-7097.

(65) Koenen HJ, Fasse E, Joosten I. CD27/CFSE-based ex vivo selection of highly suppressive alloantigen-specific human regulatory T cells. J Immunol 2005; 174(12):7573-7583.

(66) Vlad G, Cortesini R, Suciu-Foca N. License to heal: bidirectional interaction of antigen-specific regulatory T cells and tolerogenic APC. J Immunol 2005; 174(10):5907-5914.

(67) Walker MR, Carson BD, Nepom GT, Ziegler SF, Buckner JH. De novo generation of antigen-specific CD4+CD25+ regulatory T cells from human CD4+. Proc Natl Acad Sci USA 2005; 102(11):4103-4108.

(68) Jesser RD, Li S, Weinberg A. Regulatory T cells generated during cytomegalovirus in vitro stimulation of mononuclear cells from HIV-infected individuals on HAART correlate with decreased lymphocyte proliferation. Virology 2006; 352(2):408-417.

(69) Wilson NJ, Boniface K, Chan JR et al. Development, cytokine profile and function of human interleukin 17-producing helper T cells. Nat Immunol 2007; 8(9):950-957.

(70) Manel N, Unutmaz D, Littman DR. The differentiation of human T(H)-17 cells requires transforming growth factor-beta and induction of the nuclear receptor RORgammat. Nat Immunol 2008; 9(6):641-649.

(71) Acosta-Rodriguez EV, Napolitani G, Lanzavecchia A, Sallusto F. Interleukins 1beta and 6 but not transforming growth factor-beta are essential for the differentiation of interleukin 17-producing human T helper cells. Nat Immunol 2007; 8(9):942-949.

(72) McGeachy MJ, Bak-Jensen KS, Chen Y et al. TGF-beta and IL-6 drive the production of IL-17 and IL-10 by T cells and restrain T(H)-17 cell-mediated pathology. Nat Immunol 2007; 8(12):1390-1397.

(73) Volpe E, Servant N, Zollinger R et al. A critical function for transforming growth factor-beta, interleukin 23 and proinflammatory cytokines in driving and modulating human T(H)-17 responses. Nat Immunol 2008; 9(6):650-657.

(74) Arruvito L, Sanz M, Banham AH, Fainboim L. Expansion of CD4+CD25+and FOXP3+ regulatory T cells during the follicular phase of the menstrual cycle: implications for human reproduction. J Immunol 2007; 178(4):2572-2578.

(75) Kayisli UA, Selam B, Guzeloglu-Kayisli O, Demir R, Arici A. Human chorionic gonadotropin contributes to maternal immunotolerance and endometrial apoptosis by regulating Fas-Fas ligand system. J Immunol 2003; 171(5):2305-2313.

(76) Polanczyk MJ, Carson BD, Subramanian S et al. Cutting edge: estrogen drives expansion of the CD4+CD25+ regulatory T cell compartment. J Immunol 2004; 173(4):2227-2230.

(77) Polanczyk MJ, Hopke C, Vandenbark AA, Offner H. Estrogen-mediated immunomodulation involves reduced activation of effector T cells, potentiation of Treg cells, and enhanced expression of the PD-1 costimulatory pathway. J Neurosci Res 2006; 84(2):370-378.

(78) Zenclussen AC. Regulatory T cells in pregnancy. Springer Semin Immunopathol 2006; 28(1):31-39.

(79) Marazzi MC, Palombi L, et.al. Maternal Outcomes following Interruption of HAART for PMTCT: A Drug Resource Enhancement against AIDS and Malnutrition Program, Prospective Cohort from Sub-Saharan Africa 16th CROI 2009.Montreal.

(79A) Palacios R, Senise J, Vaz M, Diaz R, Castelo A. Short-term antiretroviral therapy to prevent mother-to-child transmission is safe and results in a sustained increase in CD4 T-cell counts in HIV-1-infected mothers. HIV Med 2009; 10(3):157-62.

(80) Walmsley S, Bernstein B, King M et al. Lopinavir-ritonavir versus nelfinavir for the initial treatment of HIV infection. N Engl J Med 2002; 346(26):2039-2046.

(81) King MS, Bernstein BM, Walmsley SL et al. Baseline HIV-1 RNA level and CD4 cell count predict time to loss of virologic response to nelfinavir, but not lopinavir/ritonavir, in antiretroviral therapy-naive patients. J Infect Dis 2004; 190(2):280-284.

(82) The Antiretroviral Registry Steering Committee. Antiretroviral Pregnancy Registry International Interim Report for 1 January 1989 through 31 January 2008. 2008. Wilmington, NC, Registry Coordinating Center.

(83) Cahn P. Emtricitabine: a new nucleoside analogue for once-daily antiretroviral therapy. Expert Opin Investig Drugs 2004; 13(1):55-68.

(84) Szczech GM, Wang LH, Walsh JP, Rousseau FS. Reproductive toxicology profile of emtricitabine in mice and rabbits. Reprod Toxicol 2003; 17(1):95-108.

(85) Gallant JE, Staszewski S, Pozniak AL et al. Efficacy and safety of tenofovir DF vs stavudine in combination therapy in antiretroviral-naive patients: a 3-year randomized trial. JAMA 2004; 292(2):191-201.

(86) Arribas JR, Pozniak AL, Gallant JE et al. Tenofovir disoproxil fumarate, emtricitabine, and efavirenz compared with zidovudine/lamivudine and efavirenz in treatment-naive patients: 144-week analysis. J Acquir Immune Defic Syndr 2008; 47(1):74-78.

(87) Squires K, Lazzarin A, Gatell JM et al. Comparison of once-daily atazanavir with efavirenz, each in combination with fixed-dose zidovudine and lamivudine, as initial therapy for patients infected with HIV. J Acquir Immune Defic Syndr 2004; 36(5):1011-1019.

(88) Malan N, Krantz E, David N, et.al. Efficacy and safety of atazanavir-based therapy in antiretroviral-naive HIV-1-infected subjects, both with and without ritonavir: 48-week results from AI424089. 13th Conference on Retroviruses and Opportunistic Infections, Denver, CO, February 5-8, 2006 .

(89) Molina JM, Andrade-Villanueva J, Echevarria J, et.al. Efficacy and safety of once-daily atazanavir/ritonavir compared to twice-daily lopinavir/ritonavir, each in combination with tenofovir and emtricitabine in ARV naive HIV-1-infected subjects: The CASTLE study, 48 week results. 15th Conference on Retroviruses and Opportunistic Infections, Boston, MA, February 3-6, 2008.

(89A)Heffron R, Donnell D, Rees H, et al. Use of hormonal contraceptives and risk of HIV-1 transmission: a prospective cohort study. Lancet Infectious Diseases, 2012, 12:19-26.

(89B)World Health Organization. Hormonal contraception and HIV: technical statement. 16 February 2012

(90) Cohn SE, Park JG, Watts DH et al. Depo-medroxyprogesterone in women on antiretroviral therapy: effective contraception and lack of clinically significant interactions. Clin Pharmacol Ther 2007; 81(2):222-227.

APPENDIX I: SCHEDULE OF EVALUATIONS

Evaluation[a]	Screen	Entry	4	12	24	36	48	Repeat Evals[a]	Confirmation of Virologic Failure	Premature Treatment D/C	Study D/C[e]	Step 2/3 Entry	Step 2/3 Wk+4[d]
**CLINICAL EVALUATIONS**													
Documentation of HIV Infection	X												
Medical/Medication History	X												
Clinical Assessments	X	X	X	X	X	X	X	Q12W		X	X	X	X
QOL/Adherence/Resource Utilization Questionnaire		X		X	X		X	Q24W		X	X	X	X
**LABORATORY EVALUATIONS**													
Hematology	X		X	X	X	X	X	Q12W		X	X	X	X
Urine (Dipstick for Protein)		X					X	Q48W				X	
Blood Chemistries	X		X	X	X	X	X	Q12W		X	X	X	X
Calculated Creatinine Clearance	X		X	X	X	X	X	Q12W		X	X	X	X
Liver Function Tests (LFTs)	X		X	X	X	X	X	Q12W				X	X
Hepatitis B Serology	X												
Pregnancy test[b]			X	X	X	X	X	Q12W		X		X	X
Fasting Lipid/Metabolic Profile		X			X		X	Q48W		X	X	X	
CD4+/CD8+ (Percentage/Absolute)	X			X	X	X	X	Q12W		X	X	X	
HIV-1 RNA – Step 1 Arm A		X	X	X	X	X	X	Q12W	X	X	X		
HIV-1 RNA – Step 1 Arm B		X								X	X		
HIV-1 RNA – Step 2 and Step 3			X	X	X	X	X	Q12W	X	X	X	X	X
HIV-1 Resistance Testing Results (From Medical Records)		X											
HIV-1 Resistance Testing (Real Time)									X				
**STORED SAMPLES**													
Stored sample for Inflammatory/Thrombogenic Markers		X	X	X	X		X	Q24W		X	X	X	X
Stored plasma and DBS	X	X	X	X	X		X	Q24W		X	X	X	X
Stored sample for HIV-1 Resistance Testing (from Stored Sample Above)		X	X	X	X		X	Q24W		X	X	X	X
Cryopreserved PBMC (from Stored Sample Above)[c]		X			X		X	Q48W				X	

[a] See Section 6.0 for further explanation of scheduling and evaluations, including targeted and allowable visit windows. Repeat evaluations are to be done beginning from the last scheduled time point.
[b] Serum or urine test is acceptable. Perform for women of reproductive potential at specified time points as clinically indicated/suspected, unless on EFV, in which case testing is required at every visit while on EFV and through 12 weeks after stopping EFV.
[c] Cryopreserved PBMCs are collected at all indicated time points from only the first 200 women enrolled in Step 1 at sites meeting criteria in Section 6.2.9.
[d] The Step 2/3 Week +4 visit should take place four weeks after Step 2/3 Entry. Thereafter, the q12 week visit schedule established in Step 1 (based on the date of study entry) should be resumed. If the target date for the Step 2/3 Week +4 visit falls within the targeted window for the next q12 visit, a combined visit, including all evaluations listed for the Step 2/3 Week +4 visit and the next q12 visit, should be conducted.
[e] See Section 6.1.5 for more information on final study contacts required after Study Discontinuation (D/C) visits.

APPENDIX II: MATERNAL ENDPOINT DIAGNOSES

The following AIDS-defining illnesses (WHO Clinical Stage 4), WHO Stage 2 and Stage 3 clinical events, non-AIDS-defining cancers, and other targeted medical conditions have been identified for endpoint analysis.

The occurrence of these conditions during the study may trigger the collection of additional information for inclusion in the study database. The definitions of these conditions can be found on the DMC Web Site.

WHO Stage 4 Clinical Events

- Bacterial pneumonia, recurrent, severe (> 2 episodes in 12 months)
- Candidiasis of bronchi, trachea, lungs, esophagus
- Cryptococcosis, extrapulmonary including meningitis
- Cryptosporidiosis, chronic intestinal (> 1 month duration)
- Cytomegalovirus disease (retinitis or infection of other organs)
- Encephalopathy, HIV-related
- Herpes simplex, chronic (orolabial, genital, or anorectal site, > 1 month duration), or bronchitis, pneumonitis, esophagitis, or visceral at any site
- Isosporiasis, chronic intestinal (> 1 month duration) (confirmatory diagnostic testing required)
- Leishmaniasis, atypical, disseminated
- Mycobacterium avium complex (MAC) or M. kansasii, disseminated or extrapulmonary
- Mycobacterium tuberculosis (extrapulmonary)
- Mycobacterial infection, other species or unidentified species, disseminated or extrapulmonary
- Mycosis, disseminated (extrapulmonary histoplasmosis or coccidiomycosis)
- Penicilliosis, disseminated
- Pneumocystis pneumonia
- Progressive multifocal leukoencephalopathy (PML)
- Septicemia, recurrent, including non-typhoidal Salmonella
- Toxoplasmosis of brain/central nervous system
- Wasting syndrome due to HIV (involuntary weight loss > 10% of baseline body weight) associated with either chronic diarrhea (> 2 loose stools per day > 1 month) or chronic weakness and documented fever > 1 month
- Cervical carcinoma, invasive, confirmed by biopsy
- Kaposi Sarcoma
- Lymphoma (primary central nervous system/cerebral, B cell non-Hodgkin (confirmatory diagnostic testing required))
- Atypical disseminated leishmaniasis
- Symptomatic HIV-associated nephropathy
- Symptomatic HIV-associated cardiomyopathy
- Reactivation of American trypanosomiasis (meningoencephalitis or myocarditis)

WHO Stage 3 Clinical Events

- Acute necrotizing ulcerative stomatitis, gingivitis, or periodontitis
- Unexplained severe weight loss (> 10% body weight)
- Unexplained chronic diarrhea
- Unexplained persistent fever
- Oral candidiasis, persistent
- Oral hairy leukoplakia
- Pulmonary tuberculosis
- Severe bacterial infections (other than recurrent bacterial pneumonia)
- Acute necrotizing ulcerative stomatitis, gingivitis, or periodontitis
- Unexplained anemia (hemoglobin <8 g/dL)
- Neutropenia (neutrophils <500 cells/μL)
- Chronic thrombocytopenia (platelets <50,000 cells/μL)

WHO Stage 2 Clinical Events

- Moderate, unexplained weight loss (< 10% body weight)
- Upper respiratory tract infections, recurrent (sinusitis, tonsillitis, otitis media and pharyngitis)
- Herpes zoster
- Angular cheilitis
- Oral ulcerations, recurrent
- Papular pruritic eruptions
- Seborrhoeic dermatitis
- Fungal nail infections

Non-AIDS-Defining Cancers

- Lung Cancer
- Liver Cancer
- Anal Carcinoma
- Hodgkin Lymphoma
- Oropharyngeal Carcinoma
- Melanoma
- Colorectal Carcinoma
- Breast Cancer
- Burkitts Lymphoma

Other Targeted Medical Conditions

- Pulmonary Tuberculosis
- Severe Bacterial Infections (other than recurrent bacterial pneumonia)
- Diabetes mellitus
- Lipodystrophy (lipohypertrophy or lipoatrophy)
- Dyslipidemia
    - Hypertriglyceridemia - Grade 3 and Grade 4
    - Cholesterol - Grade 3 and Grade 4
- Idiopathic thrombocytopenia
- Malaria
- Idiopathic thrombocytopenic purpura
- Sensory peripheral neuropathy
- Malignancy, newly diagnosed, excluding squamous cell and basal cell cancer of the skin
- Renal insufficiency
    - Acute
    - Chronic
- Liver disease
    - Cirrhosis
    - Idiopathic sclerosing cholangitis
- Lactic acidosis
- Symptomatic HIV-associated nephropathy
- Immune reconstitution inflammatory syndrome (IRIS)

Major Cardiovascular Disease Outcomes

- Hypertension
- Congestive heart failure
- Stroke
- Transient Ischemia Event (TIA)
- Pulmonary Embolism
- Myocardial Infarction (MI)
    - Acute symptomatic (non-fatal myocardial infarction (MI) requiring hospitalization)
    - Silent (diagnosed by serial Q-wave change on electrocardiogram (ECG))
- Coronary Artery Disease requiring percutaneous or surgical intervention
- Coronary Artery Disease requiring medical therapy
- Deep Vein Thrombosis
- Peripheral Vascular Disease
- Symptomatic HIV-associated Cardiomyopathy

APPENDIX III: TOXICITY MANAGEMENT

Unanticipated and anticipated toxicities will be graded according to the Division of AIDS Table for Grading the Severity of Adult and Pediatric Adverse Events, Version 1.0, December 2004 with Clarification dated August 2009. As described in the remainder of this appendix, site investigators will manage toxicities based on severity grade and, in some cases, relationship to study drug. Site investigators should consult on toxicity management with the study Clinical Management Committee (CMC) as directed in this appendix and may additionally consult with the CMC when needed, at their discretion. When consulting with the CMC, site investigators should follow the CMC communication procedures contained in the study Manual of Procedures. ***Information on study step or randomization arm should NOT be included in correspondence with the CMC unless this information is specifically requested by the CMC.***

General Guidelines for Management of Toxicities Not Detailed in the Guidance on Toxicity Management Tables

The following general guidelines apply to management of study drug regimens in response to all toxicities, unless superseded by directions in the Guidance on Toxicity Management Tables (provided below) that give specific information on management of the following:

- Anemia and neutropenia
- Elevated ALT or AST
- Decreased creatinine clearance
- Rash
- Elevated serum triglycerides or cholesterol

For participants for whom study drug is held for toxicity management, relevant clinical and laboratory evaluations should be repeated per the grade- or toxicity-specific guidance provided below until the toxicity resolves or is stabilized.

For participants on a triple ARV regimen, if one ARV must be held for toxicity management, all ARVs in the regimen should be held concurrently.

For pregnant women, additional clinical evaluation may be required to rule out gestational diabetes, pre-eclampsia, or other treatable causes of anemia.

Toxicities assessed as related to non-study drugs (concomitant medications) should be handled according to the relevant package inserts and the best medical judgment of the site investigator.

General Guidelines for other Grade 1 or Grade 2 Toxicities

Participants who develop a Grade 1 or Grade 2 toxicity may continue study drug without alteration, with the exceptions noted in the tables below for specific toxicities.

General Guidelines for other Grade 3 Toxicities

For Grade 3 laboratory abnormalities, the site investigator should attempt to repeat the test to confirm the Grade 3 value as soon as possible and generally within 3 working days of site awareness. If the test cannot be repeated within 3 working days, it should be repeated as soon as possible and the CMC notified when the result is available. The result of the repeat test should be used to guide management of the toxicity.

If the result of the repeat test is Grade 1 or 2, the relevant management guidelines (Grade 1 or 2) should be followed. In this case, the initial grade 3 result should be recorded on case report forms (and included in EAE reports, if applicable).

For Grade 3 clinical and laboratory toxicities, alternate explanations for the toxicity should be sought prior to holding study drug.

For Grade 3 clinical and laboratory toxicities assessed as possibly, probably or definitely related to study drug, with the exception of isolated Grade 3 hyperbilirubinemia attributed to ataznavir (ATV), the implicated study drug(s) should be replaced or the entire regimen held, unless the site investigator feels that continuation of the current regimen is in the participant's best interest. If the site investigator feels that continuation of the current regimen is in the participant's best interest, the CMC should be informed. For Grade 3 isolated hyperbilirubinemia attributed to ATV, ATV may be continued unless associated with jaundice or scleral icterus that presents an intolerable cosmetic concern to the participant.

For Grade 3 clinical and laboratory toxicities assessed as probably not or not related to study drug, study drug may be continued.

For all Grade 3 toxicities, the participant should be re-evaluated weekly until the toxicity improves to Grade $\leq$ 2 or until stabilized.

If a study drug regimen is held due to a Grade 3 toxicity, the site investigator may resume the regimen once the toxicity improves to Grade $\leq$ 2. Following resumption of study drug, if the Grade 3 toxicity recurs, the implicated study drug(s) should be permanently discontinued. If one or more study drugs are not clearly implicated, the site investigator should consult the CMC prior to permanent discontinuation.

Participants experiencing Grade 3 toxicities requiring permanent discontinuation of an implicated study drug should be re-evaluated at least weekly until improvement to Grade $\leq$ 2 or until stabilized and no longer in need of such frequent monitoring, as determined by the site investigator.

Grade 3 and 4 acute and worsening depression, including suicidal ideation and suicide attempts, have been reported infrequently with use of FTC/TDF/RPV. Participants on FTC/TDF/RPV should be counseled to report severe depressive symptoms immediately because discontinuation of FTC/TDF/RPV may be required. In the event that a participant experiences treatment-limiting (in the opinion of the site investigator) depressive symptoms attributed to FTC/TDF/RPV, FTC/TDF/RPV should be permanently discontinued.

Guidelines for Grade 4 Toxicities

For Grade 4 laboratory abnormalities, the site investigator should attempt to repeat the test to confirm the Grade 4 value as soon as possible and generally within 3 working days of site awareness. Study drug (entire regimen) should be held pending the result of the repeat test. If the test cannot be repeated within 3 working days, it should be repeated as soon as possible and the CMC notified when the result is available. The result of the repeat test should be used to guide management of the toxicity (based on severity grade).

If the result of the repeat test is Grade 1, 2, or 3, the relevant management guidelines (Grade 1, 2, or 3) should be followed. In this case, the initial grade 4 result should be recorded on case report forms (and included in EAE reports, if applicable).

For all Grade 4 toxicities, with the exception of isolated Grade 4 hyperbilirubinemia attributed to ataznavir (ATV), all study drugs should be held until improvement of the toxicity to Grade ≤ 2. Alternatively, the site investigator may continue study drug only if he or she has compelling evidence that the toxicity is NOT related to study drug. In this case, consultation with the CMC is required within 3 working days. The participant should be re-evaluated weekly until the toxicity improves to Grade ≤ 2 or until stabilized. For Grade 4 isolated hyperbilirubinemia attributed to ATV, ATV may be continued unless associated with jaundice or scleral icterus that presents an intolerable cosmetic concern to the participant.

Once a Grade 4 toxicity improves to Grade ≤ 2, use of study drug may be resumed; in this case, alternative study-provided or non-study-provided drugs should replace the implicated study drug(s). Alternatively, if the Grade 4 toxicity was assessed as probably not or not related to the study drug, the original regimen may be resumed at the discretion of the site investigator, with approval in advance from the CMC. Following resumption of study drug, if the Grade 4 toxicity recurs, the implicated study drug(s) should be permanently discontinued. If one or more study drugs are not clearly implicated, the site investigator should consult the CMC prior to permanent discontinuation.

Participants experiencing Grade 4 toxicities requiring permanent discontinuation of an implicated study drug should be followed at least weekly until improvement to Grade ≤ 2 or until stabilized and no longer in need of such frequent monitoring, as determined by the site investigator.

Grade 3 and 4 acute and worsening depression, including suicidal ideation and suicide attempts, have been reported infrequently with use of FTC/TDF/RPV. Participants on FTC/TDF/RPV should be counseled to report severe depressive symptoms immediately because discontinuation of FTC/TDF/RPV may be required. In the event that a participant experiences treatment-limiting (in the opinion of the site investigator) depressive symptoms attributed to FTC/TDF/RPV, FTC/TDF/RPV should be permanently discontinued.

Guidance on Toxicity Management Table for Specified Toxicities: Anemia & Neutropenia

CONDITION AND SEVERITY	STUDY DRUG USE	FOLLOW-UP AND MANAGEMENT
**ANEMIA AND NEUTROPENIA**		
Grade 1	Continue study drug	
Grade 2	Continue study drug (or manage as in management box)	Participants on ZDV may continue study drug unchanged or may substitute TDF or another NRTI for ZDV at the discretion of the site investigator
Grade 3 possibly related, probably not related, or not related	Continue study drug	Repeat test to confirm within 3 working days.<br>If repeat assessment is Grade ≤ 2, manage as per Grade 2.<br>If repeat assessment is Grade 3, repeat test again within 7 days. If Grade 3 persists, consult the CMC on study drug regimen and frequency of repeat assessments.
Grade 3 probably related or related<br>OR<br>Grade 4 that is not immediately life threatening	Hold all study drugs or replace suspect study drug	Repeat test to confirm within 3 working days.<br>If repeat assessment is Grade ≤ 2, manage as per Grade 2.<br>If repeat assessment is Grade 3, continue immediate action (hold all study drugs or replace suspect study drug) and consult the CMC within 3 working days on study drug regimen and frequency of repeat assessments.
Grade 4 that is immediately life threatening based on clinical findings (e.g., fever, illness)	Hold all study drugs	Repeat test to confirm within 3 working days.<br>If repeat assessment is Grade < 4, manage per the grade of the repeat assessment.<br>If repeat assessment is Grade 4, continue immediate action (hold all study drugs) and consult the CMC within 3 working days on study drug regimen and frequency of repeat assessments.

Guidance on Toxicity Management Table for Specified Toxicities: Asymptomatic AST or ALT

CONDITION AND SEVERITY	STUDY DRUG USE	FOLLOW-UP AND MANAGEMENT
**ELEVATIONS in AST or ALT**		
Grade 1<br>Asymptomatic	Continue study drug	Repeat test as soon as possible and within 14 days.<br><br>If repeat assessment is Grade ≤1, continue study drug.<br><br>If participant becomes symptomatic, follow guidance for symptomatic hepatitis.
Grade 2<br>Asymptomatic	Continue study drug	Repeat test as soon as possible and within 14 days.<br><br>Assess for alcohol use, non-study medication-related drug toxicity, lactic acidosis syndrome, pre-eclampsia, fatty liver of pregnancy, and viral hepatitis as the cause of the AST/ALT elevation. If the AST/ALT elevation is considered most likely to be due to concomitant illness or medication, standard management, including discontinuation of the likely causative agent, should be undertaken.<br><br>If repeat assessment is Grade ≤2, continue study drug.<br><br>If participant becomes symptomatic, follow guidance for symptomatic hepatitis.

Guidance on Toxicity Management Table for Specified Toxicities: AST or ALT (Cont'd)

CONDITION AND SEVERITY	STUDY DRUG USE	FOLLOW-UP AND MANAGEMENT
**ELEVATIONS in AST or ALT**		
Grade 3<br>Asymptomatic	Continue study drug	Repeat test within 3 working days.<br>Assess for alcohol use, non-study medication-related drug toxicity, lactic acidosis syndrome, pre-eclampsia, fatty liver of pregnancy, and viral hepatitis as the cause of the AST/ALT elevation.<br>If repeat assessment is Grade ≤2, manage as per Grade 2.<br>If repeat assessment is Grade 3 and is attributed to concomitant illness or medication (probably not or not related to study drug), study drug may be continued at the discretion of the site investigator. Treat the underlying illness or remove the likely causative agent.<br>If the repeat assessment is Grade 3 and is assessed as possibly, probably, or definitely related to study drug, hold study drug (entire regimen). Repeat testing weekly and once the toxicity grade is Grade ≤2, study drug may be resumed with replacement of the implicated study drug(s). If one or more study drugs are not clearly implicated, the site investigator should consult the CMC on the regimen to be resumed. Should the site investigator wish to resume an implicated study drug, consultation with the CMC is required in advance.<br>If study drug is resumed following a hold for Grade 3 AST/ALT, repeat testing should be performed one week after resumption. If the result of this testing is Grade 3 or 4, consult the CMC. Otherwise, it is not necessary to report the results to the CMC.<br>If participant becomes symptomatic, follow guidance for symptomatic hepatitis.

CONDITION AND SEVERITY	STUDY DRUG USE	FOLLOW-UP AND MANAGEMENT
**ELEVATIONS in AST or ALT**		
Grade 4<br>Asymptomatic	Hold study drug	Repeat test within 3 working days, in addition to total bilirubin and INR if available at the site.<br><br>Assess for alcohol use, non-study medication-related drug toxicity, lactic acidosis syndrome, pre-eclampsia, fatty liver of pregnancy, and viral hepatitis as the cause of the AST/ALT elevation.<br><br>If repeat assessment is Grade < 4, manage per the grade of the repeat assessment.<br><br>If repeat assessment is Grade 4, continue to hold study drug (entire regimen). Consult the CMC on study drug regimen and frequency of repeat assessments while following ALT/AST at least weekly. Once the toxicity grade is Grade ≤ 1, study drug may be resumed with replacement of the implicated study drug(s). If one or more study drugs are not clearly implicated, the site investigator should consult the CMC on the regimen to be resumed. Should the site investigator wish to resume an implicated study drug, consultation with the CMC is required in advance.<br><br>If study drug is resumed following a hold for Grade 4 AST/ALT, repeat testing should be performed one week after resumption. If the result of this testing is Grade 3 or 4, consult the CMC. Otherwise, it is not necessary to report the results to the CMC.<br><br>If participant becomes symptomatic, follow guidance for symptomatic hepatitis.

Guidance on Toxicity Management Table for Specified Toxicities:
Symptomatic Hepatitis

CONDITION AND SEVERITY	STUDY DRUG USE, FOLLOW-UP, AND MANAGEMENT
**SYMPTOMATIC HEPATITIS**	
Signs and symptoms of hepatitis include but are not limited to fatigue, malaise, anorexia, nausea, acholic stools, bilirubinuria, jaundice, liver tenderness, and/or hepatomegaly (icteric sclera in isolation without systemic complaints is not considered symptomatic)	If participant is on NVP:<br>- Immediately perform AST and ALT tests, in addition to total bilirubin and INR if available at the site. If AST or ALT has increased one or more grades above the participant's baseline value, immediately hold NVP. Also hold NVP if the participant's signs and symptoms include acholic stools, bilirubinuria, jaundice, liver tenderness, or hepatomegaly.<br>- Consult the CMC on study drug regimen and frequency of repeat assessments (in general, at least weekly re-assessment is recommended).<br>- If it is determined that the participant has clinical hepatitis with or without liver function test abnormalities and NVP cannot be excluded as the cause, NVP should be permanently discontinued.
	If participant is not on NVP:<br>- Immediately perform AST and ALT tests, in addition to total bilirubin and INR if available at the site; follow general management guidelines based on the highest grade sign or symptom.<br>- Consult the CMC on study drug regimen and frequency of repeat assessments (in general, at least weekly re-assessment is recommended).

Guidance on Toxicity Management Table for Specified Toxicities: Creatinine Clearance

CONDITION AND SEVERITY	STUDY DRUG USE	FOLLOW-UP AND MANAGEMENT
**CREATININE CLEARANCE (CrCl)**		
Estimated CrCl < 50 (initial value)	Continue study drug unless participant is symptomatic	Repeat test and CrCl calculation (Cockcroft-Gault formula) as soon as possible (and within 1 week)
Confirmed CrCl < 50	Manage study drug as defined here or in package inserts	Participants with a confirmed CrCl rate < 50 mL/min should undergo a thorough evaluation for potential causes of decreased renal function in addition to receiving treatment, as appropriate. May substitute ZDV or d4T or ABC for TDF with appropriate renal dosing adjustments (see below) while the etiology of the renal insufficiency is being investigated and renal function is being closely followed. Consult the CMC as needed on evaluating causes of renal insufficiency and potential relationship to TDF.<br><br>If TDF is the only potential cause of renal insufficiency found, TDF should be permanently discontinued.<br><br>Follow weekly until CrCl rate returns to ≥60 mL/min. Once CrCl rate is ≥60 mL/min, and if the renal insufficiency was ascribed to etiologies other than TDF, TDF-containing regimens may be resumed with careful monitoring* of renal function.<br><br>If the CrCl remains < 60, and TDF has been excluded as a cause of the renal insufficiency, after consultation with the CMC, a TDF-containing regimen may be restarted with careful monitoring* and appropriate renal dosing adjustments of the drugs in the regimen.<br><br>*Careful monitoring of renal function should include weekly re-assessment of CrCl for one month and monthly re-assessment for the next three months.<br><br>For Lamivudine (3TC), recommended renal dosing adjustments are as follows:<br>• If CrCl ≥50, 150 mg twice daily or 300 mg once daily<br>• If CrCl 30-49, 150 mg once daily<br>• If CrCl 15-29, 150 mg first dose, then 100 mg once daily<br>• If CrCl 5-14, 150 mg first dose, then 50 mg once daily<br>• If CrCl <5, 50 mg first dose, then 25 mg once daily<br><br>For Stavudine (d4T), recommended renal dosing adjustments (assuming a starting dose of 30 mg every 12 hours) are as follows:<br>• If CrCl >50, 30 mg every 12 hours<br>• If CrCl 26-50, 15 mg every 12 hours<br>• If CrCl 10-25, 15 mg every 24 hours

Guidance on Toxicity Management Table for Specified Toxicities:
Rash

CONDITION AND SEVERITY	STUDY DRUG USE	FOLLOW-UP AND MANAGEMENT
**RASH**		
Grade 1 or Grade 2	If on NVP, EFV, or abacavir: study drugs may need to be held depending on rash distribution and relatedness assessment	If the rash is generalized and there is no definitive explanation for the rash:<br>• Hold study drug (entire regimen)<br>• Test ALT within 3 working days, and<br>• Evaluate for symptoms of clinical hepatitis and hypersensitivity reaction.<br>If any clinical symptoms of hepatitis or ALT elevation or hypersensitivity reaction, permanently discontinue NVP, EFV, or abacavir and consult with CMC on study drug regimen.<br><br>If the rash is not generalized or if there is a definitive explanation for the rash (e.g., varicella), study drug may be continued with no additional evaluation required.
	If not on NVP, EFV, or abacavir: continue study drug	May be treated symptomatically, but should be monitored closely by the site investigator.
Grade 3	Hold all study drugs unless the rash is determined to be unrelated to study drug	If there is no definitive explanation for the rash (e.g., varicella), test ALT and manage per the ALT/AST elevation table.<br>If on NVP, EFV, or abacavir, permanently discontinue this/these drugs. When the rash resolves, study drug may be resumed (except abacavir or NVP or EFV).
Grade 4	Hold all study drugs	Consult the CMC on possible alternative study drug regimens.

Guidance on Toxicity Management Table for Specified Toxicities: Elevated Serum Triglycerides or Cholesterol

CONDITION AND SEVERITY	STUDY DRUG USE	FOLLOW-UP AND MANAGEMENT
**ELEVATED SERUM TRIGLYCERIDES OR CHOLESTEROL**		
Any Grade 1 or Grade 2	Continue study drug	
Initial Grade 3 or Grade 4	Continue study drug	Repeat assessment within 2 weeks with participant fasting for a minimum of 8 hours.
Persistent Grade 3 or Grade 4	May continue study drug with dietary and exercise counseling.	Site investigator may consider fibric acid derivative or nicotinic acid for triglyceride elevation or allowed HMG-CoA reductase inhibitor for cholesterol elevation.

Management of Immune Reconstitution Inflammatory Syndromes

Inflammatory syndromes have been reported to occur shortly after the initiation of potent combination ART. When these syndromes are suspected the following management plan should be followed, and consultation with the study CMC is recommended:

- Continue ARV treatment.
- Confirm diagnosis of opportunistic infection (OI).
- Continue or initiate specific therapy for the infection.
- Evaluate the participant clinically to exclude a new infectious process if the participant was already receiving therapy for the OI.
- Initiate anti-inflammatory agents, initially nonsteroidals or, if needed corticosteroids at the discretion of the site investigator in consultation with the CMC.

APPENDIX IV: SAMPLE INFORMED CONSENT FOR SCREENING AND ENROLLMENT

IMPAACT 1077HS
HAART Standard Version of the PROMISE Study
(Promoting Maternal and Infant Survival Everywhere)
Protocol Version 2.0, Dated 9 October 2012

Note to Sites: Version number and date of the protocol should be included on the first page of the protocol and the version number and date of the consent form should be included in a header or footer on each page along with page numbering in the following format: Page 1 of x, Page 2 of x, Page 3 of x.

SHORT TITLE FOR THE STUDY: PROMISE HAART-Standard Version

INTRODUCTION

You are being asked to take part in this research study because:

- you are infected with human immunodeficiency virus (HIV), the virus that causes AIDS
- you are pregnant or recently completed a pregnancy
- you are taking or took a combination of medicines during pregnancy (known as highly active antiretroviral therapy or HAART) to try to keep your baby from getting HIV

This study is sponsored by the U.S. National Institutes of Health (NIH). The doctor in charge of this study at this site is: [insert name of site Principal Investigator]. Before you decide if you want to join this study, we want you to know about the study. We will explain the study to you and you are free to ask questions at any time. We will ask if you want to join the study. If you do want to join, we will ask you to sign this consent form and you will be given a copy to take home with you.

WHY IS THIS STUDY BEING DONE?

In some countries, such as the US and Brazil *[sites may add other study countries, i.e., Argentina, Botswana, China, Haiti, Peru, Thailand]*, women with HIV who become pregnant are given a combination of HIV medicines (HAART) to try to keep their babies from getting HIV. When these women are no longer pregnant, a decision must be made to either continue taking HAART or to stop taking HAART. For women with high CD4+ cell counts (counts of cells that fight HIV), HAART is usually stopped after pregnancy. However, it is not known if it is better for women to stop or continue taking HAART after pregnancy. The main purpose of this study is to answer that question.

Some studies in nonpregnant people have shown that it is better to continue taking HAART once started, rather than stopping, but people in these studies often had much lower CD4+ cell counts and had been on HAART longer than women who take it only during pregnancy. Some other studies have not shown that continuing HAART is better than stopping. To see if stopping HAART is better, worse, or the same in the long run compared to continuing HAART, in this study we will see how women who stop HAART after pregnancy do compared to women who continue taking HAART after pregnancy.

There are a number of other goals of the study including:

- To see how well the women will be able to take HAART after pregnancy and how that relates to remaining healthy and having a low amount of the HIV in blood.
- To see if one or a combination of blood tests that measure how the immune system (the system that fights infections) is affected by stopping or continuing HAART after pregnancy. The study will also find out if these tests can help identify which women might benefit the most from continuing HAART after pregnancy.
- To see if there are differences in the number of illnesses (for example, infections, heart disease, kidney disease, liver disease), and abnormal blood tests in women who stop HAART compared to women who continue HAART after pregnancy.
- To see if there are differences in the chances of developing resistance to HIV medicines in women who stop HAART compared to women who continue HAART after pregnancy.
- To see if there are differences in quality of life and costs of health care in women who stop HAART compared to women who continue HAART after pregnancy.

This study has been approved by the [*sites—add local ethics committee or IRB information]*. An Institutional Review Board (IRB), or Ethics Committee (EC), is a special committee that watches over the safety and rights of research participants.

WHAT DO I HAVE TO DO IF I AM IN THIS STUDY?

Screening

If you decide that you want to join the study, we will first do some screening tests to see if you are eligible. The screening tests will be done in the last trimester of pregnancy or within 42 days after you are no longer pregnant.

- We will collect medical history information and perform a physical exam.
- We will do some blood tests to look at your CD4+ cell count and how your liver and kidneys are working.
- If your medical history does not have documentation of hepatitis B tests done within the last 12 months, we will do these tests.

If You Do Not Enroll into the Study

If the tests show that you are not eligible to participate, you will continue to receive care from your usual provider, and they will help you figure out if you should continue taking HAART after your pregnancy.

If you decide not to take part in this study or if you do not meet the eligibility requirements, we will still use some of your information. As part of the screening tests, some demographic (e.g., age, gender, race), clinical (e.g., disease condition, diagnosis), and laboratory (e.g., CD4+ cell count, viral load, HIV resistance testing) data are being collected from you so that the researchers may determine whether there are patterns or common reasons why people do not join the study**.**

Study Visits if You Enroll into the Study

If you join the study, you will be randomly assigned *[sites—insert locally relevant description here, such as, "like flipping a coin"]* to one of the study groups: either the Stop HAART Group or the Continue HAART Group. You and the study staff will know which group you are in.

If you are assigned to the Stop HAART Group, the study staff will discuss how and when to stop the HIV medicines you took during pregnancy.

If you are assigned to the Continue HAART Group, you can remain on the HIV medicines you took during pregnancy or switch to HIV medicines provided by the study. In some cases, depending on the combination of HIV medicines you took during pregnancy, you will be switched to a different combination. You will also be offered different HIV medicines if you have side effects or if your HIV is not responding to the medicines you are taking.

The preferred study-supplied HAART regimen is lopinavir/ritonavir (LPV/RTV) plus fixed dose combination tenofovir/emtricitabine (TDF/FTC). Additional HIV medicines available in this study include fixed dose combination tenofovir/emtricitabine/rilpivirine (TDF/FTC/RPV), fixed dose combination lamivudine/zidovudine (3TC/ZDV), lamivudine (3TC), tenofovir (TDF), zidovudine (ZDV), didanosine (ddI), atazanavir (ATV), raltegravir (RAL), and ritonavir (RTV). All of these medicines may not be available in all countries where the study is taking place but all will be available in the countries where they are approved by national drug authorities. The study staff will tell you which medicines are available in your country.

Other HIV medicines that are not provided by the study may be used if the combination of medicines is considered effective and the medicines are provided by prescription.

**Stop HAART Group**		**Continue HAART Group**
The HIV medicines are stopped after delivery.	OR	The HIV medicines are continued for the rest of the study.

The first study visit (the day when you join) will be within 42 days after you are no longer pregnant. After that, you will have a study visit at week 4, week 12, and then approximately every 3 months until the end of the study.

Each study visit will last about [*sites—insert local information on time for study visits]*. You will have routine medical check-ups at the study clinic. It is important that you return for all of these study visits. If you do not come for a study visit or if a test result comes back abnormal, the outreach worker will contact you to find out how you are doing [*Sites: please indicate how participants may be contacted (i.e., phone, text, and/or home visit)]*. If at any time, you become sick you should let the study nurse or doctor know right away.

If you are assigned to the Stop HAART Group, and then your HIV gets worse and you are advised to start HAART, the study will provide HIV medicines to you or you may choose to take HIV medicines from other programs or providers outside the study. You will be seen for a visit one month after starting these medicines to check for any side effects and to check how they are working at treating HIV. Then you will go back to having study visits every 3 months.

Tests and Procedures at the Study Visits

- Medical history, questionnaires, and physical exam
  We will ask you about your medical history and about any medications you have taken in the past and about how well you are taking your HIV medicines, if on them. You will have a physical exam.

- Blood collected
  Blood will be collected from you for various tests. Some tests are to measure how well HIV medicines are controlling the virus. For some of the blood tests, you will be asked to fast prior the study visit. You will have approximately 45 mL (about 3 tablespoons) of blood taken at most visits. You will be given the results of these tests as soon as they are available.

- Pregnancy test
  You may be asked to give blood (about 1 mL or less than 1 teaspoon) or urine to test for pregnancy at some visits. If you take an HIV medicine called efavirenz (EFV), you will have a pregnancy test at every visit while taking this medicine and for 3 months thereafter.

- Stored Blood
  Some of your blood will be stored for testing that is planned to be done after the study is completed. After all of the planned testing is done, some of your blood may be leftover. Separately, you will be asked permission for this leftover blood to be stored and used for other research related to HIV. If you agree, you will sign a separate consent form for this. You can still be in the PROMISE study if you do not agree to have your leftover blood stored and used after all planned study testing is completed.

After your last study visit, the study staff will contact you to give you the results of tests done at your last visit. If you become pregnant during the study, and are still pregnant at your last study visit, the study staff will contact you again to find out the outcome of your pregnancy.

OTHER INFORMATION

The information collected in this study may be used for other research approved by the US NIH-sponsored research group that is conducting this study, the International Maternal Pediatric Adolescent AIDS Clinical Trials (IMPAACT) group.

HOW MANY PEOPLE WILL TAKE PART IN THIS STUDY?

About 2,000 women will take part in this study in Argentina, Botswana, Brazil, China, Haiti, Peru, Thailand and the US.

HOW LONG WILL I BE IN THIS STUDY?

You will be in the study anywhere from 2 years to almost 6 years, depending on when you join. Most women will be in the study for less than 4 years.

WHY MIGHT THE DOCTOR TAKE ME OFF THIS STUDY EARLY?

The study doctor may need to take you off the study early without your permission if:

- the study is cancelled by IMPAACT, the U.S. National Institutes of Health (NIH), the Office for Human Research Protections (OHRP), the drug companies supporting this study, the local Ministry of Health, or the site's Institutional Review Board (IRB) or Ethics Committee (EC).
- a Data Safety Monitoring Board (DSMB) recommends that the study be stopped early (a DSMB is an outside group of experts who monitor the study).

The study doctor may also need to take you off the study HIV medicines if:

- you are not able to attend the study visits
- you are not able to take the HIV medicines as required by the study
- continuing the HIV medicines may be harmful to you
- you need a treatment that you may not take while on the study

If you must permanently stop taking HIV medicines before your study participation is over, the study staff will discuss other options that may be of benefit to you. The study doctor will ask you to continue to be part of the study and return for some study visits.

AFTER THE STUDY

After you have finished your study participation, the study will not be able to continue to provide you with HIV medicines. If continuing to take these or similar medicines would be of benefit to you, the study staff will discuss how you may be able to obtain them [sites insert local information here].

WHAT ARE THE RISKS OF THE STUDY?

Taking part in this study may involve some risks and discomforts. These include possible side effects of HIV medicines, possible risks and discomforts from the study tests, and possible risks to your privacy. More information is given on each of these types of risks below.

<u>Side Effects of HIV Medicines</u>

Women in the study who take HIV medicines will take a combination of at least 3 different medicines. Some of the medicines are combined together in one tablet, others come in separate tablets. Until you join the study, we will not know what specific medicines you may take. Therefore, this form gives information about all the HIV medicines that may be given in the study (see list on page [3] of this form).

Each HIV medicine can cause side effects, when taken alone and when taken in combination. Some side effects are minor, while others can be severe. Some are common, while others are rare. If you join the study, the study staff will tell you about the side effects of the specific medicines you will take. They will check for side effects during study visits and tell you what to do if you have any side effects.

First you should know about the possible severe side effects. These effects are rare, but they can cause serious health problems and can result in death:

- Severe rash. This can be caused by atazanavir, lopinavir/ritonavir, ritonavir, and raltegravir.
- Severe depression, including suicidal thoughts or acts. This can be caused by rilpivirine.
- Abnormal heart beat, which can result in lightheadedness, fainting, and serious heart problems. This can be caused by atazanavir, lopinavir/ritonavir, and ritonavir.
- Inflammation of the pancreas. The pancreas is an organ near the stomach. When the pancreas becomes inflamed, it can cause pain in the belly, nausea, vomiting, and increased fats in the blood. This can be caused by didanosine, lamivudine, lopinavir/ritonavir, ritonavir, and tenofovir/emtricitabine.
- Inflammation of the liver. The liver is an organ near the stomach. When the liver becomes inflamed, it can cause pain and swelling in the belly, nausea, and vomiting. This can be caused by lamivudine, lopinavir/ritonavir, ritonavir, tenofovir, and zidovudine.
- Lactic acidosis, enlargement of the liver, and fatty liver, which can result in liver failure. Lactic acidosis is an imbalance in the blood that can cause weight loss, pain in the belly, nausea, vomiting, tiredness, weakness and difficulty breathing. When the liver is enlarged, it can cause pain especially on the right side of the belly, swelling in the belly, nausea, vomiting, and loss of appetite. It can also cause bleeding problems that can result in vomiting blood or dark colored stools. Fatty liver is when healthy liver cells are replaced with fat. Sometimes it causes the liver to be enlarged, but doctors usually find out about it from tests of the blood. These effects can be caused by didanosine, emtricitabine, lamivudine, tenofovir, and zidovudine. They occur more often in women, pregnant women, people who are overweight, and people who already have liver problems.
- Kidney damage or failure. The kidneys are organs near the middle of your back (one on each side). Doctors usually find out about kidney damage from tests of the blood. These effects can be caused by tenofovir.

You should also know about the more common side effects, which are not severe. There are many possible mild and moderate side effects. Some people who take HIV medicines have some of these effects, other people have different effects. The more common mild and moderate side effects are listed on the next page. This list is not a complete list of all side effects for all HIV medicines. As a reminder, if you join the study, the study staff will tell you about the side effects of the specific HIV medicines you will take.

Overall Body Effects	Effects on Your Muscles and Bones
• Overall weakness, tiredness, or feeling unwell<br>• Loss of appetite<br>• Loss of weight<br>• Changes in the placement of body fat, such as enlargement of the neck, stomach, and breasts and thinning of the arms, legs, and cheeks<br>• Numbness or tingling in the hands, arms, feet, legs, or around the mouth<br>• Pain in the hands or feet<br>• Allergic reaction<br>• Fever	• Aches or pains<br>• Loss of muscle<br>• Muscle weakness<br>• Clumsiness or lack of coordination<br>• Bone thinning or softening (which could increase the chance of breaking a bone)
**Effects on Your Skin**<br>• Rash, with or without itching<br>• Yellowing of the skin<br>• Darkening of the palms and soles of feet	**Effects on Your Blood**<br>• Decreased blood cells<br>• White blood cells help fight infection.<br>• Red blood cells help store and transport energy through the body. Low red cells can cause weakness, tiredness, and dizziness.<br>• Decreased clotting / increased bleeding<br>• Increased blood sugar or development of diabetes<br>• Increased fats in the blood that may increase the risk of heart problems<br>• Other changes in blood test results that may indicate problems with the muscles, kidneys, liver, pancreas, or gall bladder. The blood tests that may be affected include tests of how well these organs are working, tests of substances made by these organs, and tests of fats in the blood.
**Effects on Your Head**<br>• Headache<br>• Runny nose<br>• Yellowing of the eyes<br>• Not seeing normally<br>• Changes in the sense of taste<br>• Swelling of the face, lips, or tongue	
**Effects on Your Chest**<br>• Cough<br>• Shortness of breath<br>• Heartburn	**Effects on Your Mind or Mental Function**<br>• Trouble sleeping<br>• Unusual dreams<br>• Depression<br>• Anxiety or paranoia<br>• Dizziness
**Effects on Your Belly**<br>• Pain or discomfort in the belly<br>• Nausea<br>• Vomiting<br>• Gas<br>• Loose or watery stools<br>• Inflammation of the gall bladder. The gall bladder is an organ near the stomach. If it becomes inflamed, it can cause severe pain.<br>• Stones in the gall bladder or kidneys. If these stones form, they can cause severe pain.	

Other Possible Risks of HIV Medicines

Risk of Resistance: All HIV medicines can cause resistance. When resistance occurs, a medicine no longer works against HIV, which can limit the choices of medicines a person can take against HIV in the future. To avoid resistance, it is important to take HIV medicines as instructed, and not miss doses.

Risk of Immune Reconstitution Syndrome: In some people with advanced HIV infection, signs and symptoms of inflammation from other infections may occur soon after HIV medicines are started.

Risks with Hepatitis B: Some HIV medicines are active against Hepatitis B (including lamivudine, emtricitabine, and tenofovir). For women who have Hepatitis B, and take these medicines, there are some risks. The Hepatitis B could become resistant and harder to treat. Also, stopping the medicines could cause the Hepatitis B to worsen. If this happens, most women get better quickly without treatment, but in rare cases this has resulted in death.

Risks with Contraception: Some HIV medicines can interfere with some contraceptive methods, including pills, injections (shots), and implants (placed under the skin). Because of this, it may be necessary to use different or additional contraceptive methods while taking HIV medicines. The study staff will tell you about the effects of the specific HIV medicines you will take and discuss reliable contraceptive methods with you.

Risks with Pregnancy: If you wish to conceive or if you think you may be pregnant at any time during the study, please tell the study staff right away. The study staff will talk with you about the best HIV treatment options for you.

If you get pregnant during the study you can continue in the study. If you are taking HIV medicines when you get pregnant, you can continue taking these medicines or you can receive other treatment according to the local guidelines. If you are not taking HIV medicines when you get pregnant, you will be advised to start the HIV medicines usually given to pregnant women in your area to prevent mother-to-child transmission of HIV. If you get pregnant while taking HIV medicines given to you by the study, you will be asked to sign a separate consent form to continue receiving those medicines while you are pregnant. Site staff will discuss with you what is known about using the medicines during pregnancy and what risks there might be. If you are taking lopinavir/ritonavir (LPV-RTV), your dose may be increased in the last trimester of pregnancy.

Risks of Treatment Interruption

The long-term health effects of stopping HAART after taking HAART during pregnancy in women with CD4+ counts above 400 are not well described. The following are some of the possible risks associated with stopping HAART after pregnancy.

Stopping HAART may lead to virus rebound and resistance. "Virus rebound" means that the HIV viral load goes back up or "rebounds" to detectable levels. "Resistance" means that a medicine no longer works against HIV. Resistance is the greatest risk of stopping and restarting HIV medicines several times.

It is possible that your CD4+ count may go down and that your HIV disease may progress more quickly when HAART is stopped, however this risk is lower if you have a high CD4+ count when you start HAART. It is also possible that the number of cells in which the virus remains latent (hidden or resting) may increase.

If you are assigned to the Stop HAART Group, you will be followed closely and be advised to re-start HAART if your HIV gets worse or you develop HIV-related infections or diseases that can be helped by HAART.

A recent study has shown that taking HAART can make it much less likely for a person with HIV to pass HIV to a sexual partner. Based on that study, in April 2012 the World Health Organization (WHO) issued new recommendations for couples in which one partner has HIV and the other does not. The WHO recommends that all couples have HIV counseling and testing and that, if one partner has HIV and the other does not, the partner with HIV should start taking HAART even if his or her CD4+ cell count is above 350. In the PROMISE study, if you are assigned to stop HAART after pregnancy, you may be more likely to pass HIV to a sexual partner than if you continued HAART after pregnancy. In relation to the new WHO recommendations, over the coming months, health officials in each country where the PROMISE study is ongoing will need to decide whether to change their HIV treatment programs to offer HAART to people with CD4+ cell counts above 350 whose partners do not have HIV. When these decisions are made, we will tell you about them.

<u>Risks of Blood Draws</u>

Drawing blood may commonly cause discomfort, pain, dizziness or local bruising and rarely a local infection.

<u>Possible Risks to Your Privacy</u>

We will make every effort to protect your privacy while you are in this study. Your visits here will take place in private. However, it is possible that others may learn of your participation here and, because of this, may treat you unfairly. There also is a risk to your privacy if someone else taking part in this study knows you.

<u>Other Risks</u>

There may be other risks associated with taking part in this study that are not known at this time.

ARE THERE BENEFITS TO ME BY TAKING PART IN THIS STUDY?

If you take part in this study, there may be a direct benefit to you. Some but not all studies suggest that people who initiate HAART when they have high CD4+ cell counts and are in good health and remain in treatment can live longer, delay their progression to AIDS, and have lower chances of heart, kidney, and liver complications from their HIV disease. A recent study also suggests that taking HAART can make it much less likely to pass HIV to a sexual partner. It is important for you to understand that no guarantee can be made. It is also possible that you may receive no benefit from being in this study or that your health can worsen if you don't take HIV medicines as prescribed or develop resistance to the HIV medicines. Information learned from this study may help others who have HIV.

WHAT OTHER CHOICES DO I HAVE BESIDES THIS STUDY?

Joining or continuing in this study is voluntary. Instead of being in this study, you have the choice *[sites: fill in information about available alternatives at the site].* Please talk to your doctor about these and other choices available to you. Your doctor will explain the risks and benefits of these choices.

You will continue to receive regular care whether or not you take part in the study.

WHAT ABOUT CONFIDENTIALITY?

Every effort will be made to keep your personal information confidential. This personal information may be disclosed if required by law. Any publication of this study will not use your name or identify you personally.

US domestic sites only: insert the following:

To help us protect your privacy, we have obtained a Certificate of Confidentiality from the National Institutes of Health. With this Certificate, the researchers cannot be forced to disclose information that may identify you, even by a court subpoena, in any federal, state, or local civil, criminal, administrative, legislative, or other proceedings. The researchers will use the Certificate to resist any demands for information that would identify you, except as explained below. The Certificate cannot be used to resist a demand for information from personnel of the United States Government that is used for auditing or evaluation of federally funded projects or for information that must be disclosed in order to meet the requirements of the federal Food and Drug Administration (FDA).

People who may review your records include the site IRB (insert name of site IRB), the US National Institutes of Health, the Office for Human Research Protections (OHRP), study staff, study monitors, drug companies supporting the study, and their designees.

You should understand that a Certificate of Confidentiality does not prevent you or a member of your family from voluntarily releasing information about you or your participation in this research. If an insurer, employer, or other person obtains your written consent to receive research information, then the researchers may not use the Certificate of Confidentiality to withhold that information.

(The researchers should include language such as the following if they intend to make voluntary disclosure about things such as child abuse). The Certificate of Confidentiality does not prevent the researchers from disclosing voluntarily, without your consent, information that would identify you as a participant in the research project under the following circumstances. (The researchers should state here the conditions under which voluntary disclosure will be made.)]

The outreach workers may contact you so we need to know the best way to reach you (such as home visit or phone call). Your records may be reviewed by the [insert name of site] IRB or ethics committee, [your country's national health agency], National Institutes of Health (NIH), IMPAACT, ACTG, the US Office of Human Research Protections (OHRP), study staff, study monitors, and drug companies supporting this study.

WHAT ARE THE COSTS TO ME?

There is no cost to you for your study visits, exams, or blood tests. There is no cost to you for HIV medicines given to you from the study. If you take HIV medicines from another program or provider outside the study, you or your health insurance will need to pay for the medicines, unless the medicines are available free of charge. The study cannot pay for medicines obtained from other programs or providers.

WILL I RECEIVE ANY PAYMENT?

If you have to come to the hospital because of your participation in the study, your transportation and time will be reimbursed to you. You will receive *[sites: insert amount]* for each study visit.

WHAT HAPPENS IF I AM INJURED?

If you are injured as a result of being in this study, you will be given immediate treatment for your injuries *[sites: add local information regarding treatment for injury]*. There is no program for compensation either through this study or the sponsor of the study, the US National Institutes of Health (NIH). *[Sites: do not delete the statement that the sponsor will not provide compensation for injury]*. You will not be giving up any of your legal rights by signing this consent form.

WHAT ARE MY RIGHTS AS A RESEARCH PARTICIPANT?

Taking part in this study is completely voluntary. You may choose not to participate in this study or leave this study at any time. You will continue to receive care no matter what you decide.

We will tell you about new information from this or other studies that may affect you and your health, welfare, or willingness to stay in this study. If you want to be informed about the results of this study, the study staff *[will contact you when these are available--sites: include local information about how participants can find out about study results if applicable]*.

WHAT DO I DO IF I HAVE QUESTIONS OR PROBLEMS?

For questions about this study or a research-related injury, contact:
- *Site: insert name of the investigator or other study staff*
- *Site: insert telephone number of above*

For questions about your rights as a research participant, contact:
- *Site: insert name or title of person on the Institutional Review Board (IRB) or other organization appropriate for the site*
- *Site: insert telephone number of above*

Addendum to IMPAACT 1077HS APPENDIX IV
SAMPLE INFORMED CONSENT FOR SCREENING AND ENROLLMENT
For US Sites Only

As you consider whether to take part in the PROMISE study, there is some new information about taking HAART that you should know about. In March 2012, the expert panel that develops treatment guidelines for the US issued some updated guidelines and the following changes were made:

- People with CD4 cell counts below 350 should take HAART. This continues to be a strong recommendation based on clear evidence from multiple randomized clinical trials of benefit from HAART for people with CD4 cell counts below 350.

- People with CD4 cell counts between 350 and 500 should take HAART. This is now a strong recommendation based on evidence from observational (non-randomized) research studies and one randomized clinical trial. The evidence for this recommendation is increasing but is less strong than the evidence for people with CD4 cell counts below 350.

- For people with CD4 cell counts above 500, the panel gave a moderate recommendation to take HAART. This recommendation is less strong, and is based on expert opinion, because there have been no research studies for people with CD4 counts above 500.

For people in all three ranges of CD4 cell counts, the panel's recommendations to take HAART are based on the benefits of HAART for the person taking it and evidence that taking HAART can make it much less likely for a person with HIV to pass the virus to a sexual partner.

What do the updated treatment guidelines mean in relation to the PROMISE study? There are a few things to consider:

- Only women with CD4 cell counts above 400 prior to taking HAART in their current pregnancy and at the time of entry into the study can take part in the study.

- Women in the Continue HAART Group will be given HAART through the study regardless of their CD4 cell count.

- Women in the Stop HAART Group will not be given HAART through the study unless their CD4 cell count falls below 350 cells or they develop an illness for which HAART is recommended. This is being done is to better understand the risks and benefits of HAART for women with CD4 cell counts above 350. Based on the updated treatment guidelines, if women in the Stop HAART Group were being treated outside of the study, they would be recommended to take HAART if their CD4 cell count was between 350 and 500. They might also be recommended to take HAART if their CD4 cell count was above 500.

The PROMISE study is being done to provide evidence from a randomized clinical trial on whether continuing HAART should be recommended for women who took HAART during pregnancy for prevention of mother-to-child transmission. As an alternative to the options available in the study, starting on HAART for your own health is an option available to you outside the study. This is something you should consider and discuss with the study staff and your regular health care provider before deciding whether to take part in the study. Please ask any questions you may have and only decide whether to take part in the study after all of your questions have been answered.

SIGNATURE PAGE FOR PARTICIPANTS OF LEGAL AGE TO PROVIDE INDEPENDENT INFORMED CONSENT

If you have read this consent form (or had it explained to you), all of your questions have been answered, and you agree to take part in this study, please sign your name below.

______________________________	______________________________
Participant's Name (print)	Participant's Signature and Date
______________________________	______________________________
Study Staff Conducting Consent Discussion (print)	Study Staff Signature and Date
______________________________	______________________________
Witness's Name (print) (As appropriate)	Witness's Signature and Date

SIGNATURE PAGE FOR PARTICIPANTS BELOW LEGAL AGE TO PROVIDE INDEPENDENT INFORMED CONSENT

Participant Assent

If you have read this consent form (or had it explained to you), all of your questions have been answered, and you agree to take part in this study, please sign your name below.

______________________________	______________________________
Participant's Name (print)	Participant's Signature and Date

Parent/Legal Guardian Consent

If you have read this consent form (or had it explained to you), all of your questions have been answered, and you agree for your child (named above) to take part in this study, please sign your name below.

______________________________	______________________________
Parent/Guardian Name (print)	Parent/Guardian Signature and Date
______________________________	______________________________
Study Staff Conducting<br>Consent Discussion (print)	Study Staff Signature and Date
______________________________	______________________________
Witness's Name (print)<br>(As appropriate)	Witness's Signature and Date

APPENDIX V: SAMPLE INFORMED CONSENT FOR SPECIMEN STORAGE AND FUTURE USE

IMPAACT 1077HS
HAART Standard Version of the PROMISE Study
(Promoting Maternal and Infant Survival Everywhere)
Protocol Version 2.0, Dated 9 October 2012

Note to Sites: Version number and date of the protocol should be included on the first page of the consent form and the version number and date of the consent form should be included in a header or footer on each page of the consent form along with page numbering in the following format: Page 1 of x, Page 2 of x, Page 3 of x.

SHORT TITLE FOR THE STUDY: PROMISE HAART-Standard Version

INTRODUCTION

You have decided that you will participate in this research study to help us find the best ways to keep mothers healthy after receiving anti-HIV drugs to prevent their babies from being infected with HIV. In addition to the tests that you have as part of the study, we are asking now for your permission to save some blood and cells from your blood for future use. These specimens would be saved in a place called a repository, which is a special laboratory with freezers to store the specimens.

Researchers can learn a lot from a study, but as time goes by the tests that they use get better or brand new tests are developed, and more can be learned with these better or new tests by using them on stored specimens. If a researcher wants to do a test on specimens from the repository in the future, he or she will write up the idea and it will have to be approved by the leaders of the study team and other groups to make sure the research is worthwhile. If the idea is approved, then coded specimens and coded information will be given to the researcher. They would never know your name.

Because of the location of the repositories and/or the place where the tests will be conducted, these stored samples may be shipped to another country for storage and/or future use.

WHAT ABOUT CONFIDENTIALITY?

There are no names on any of the specimens, only a special study number. The people who run the repository and the scientists who later use the specimens will not know your name or any other information about you that might identify you. As explained when you agreed to join the study, records associated with the specimens may be reviewed by the study sponsor (the US National Institutes of Health) or its agents, the US Office for Human Research Protections (OHRP), pharmaceutical supporters, national regulatory authorities and the institutional review board (IRB) or ethics committee (EC) that oversee the research.

HOW OFTEN WILL THESE SPECIMENS BE COLLECTED?

As described to you when you agreed to join the study, blood will be collected for study tests at each study visit. After all testing that is planned to be done for the study has been completed, some of your blood and cells from your blood may be leftover. It is these leftover specimens that you are being asked to have stored for future use. You are not being asked to give additional specimens for long term storage and future use.

WHAT KIND OF TESTS MIGHT BE DONE ON MY SPECIMENS?

Tests that might be done include tests to see how much HIV is in the blood, how the body responds to HIV, how HIV causes disease, the levels of HIV drugs in the blood, and how drugs cause side effects. The tests might also look at other infections like malaria or other conditions like diabetes that people with HIV may get. The tests might look at how a person's genetic makeup (your DNA) either protects them or puts them at greater risk. This kind of information is important for scientists who are working on an HIV vaccine.

WILL I GET THE RESULTS OF THESE TESTS?

Most of the time, you will not get results from these tests. This is because research can take a long time. Results from research using the specimens may not be ready for many years.

The researchers who use stored samples for a study approved by NIH will not contact you with the results of their study because they will use samples with codes and will not know who to contact. If their findings could provide important information for your medical care, then the investigators would contact the research staff at your site with the results, and the staff at your clinic can link the code with your name and notify you of the results. If you would like to be contacted with this sort of information, you must notify the site staff of any changes in your address or phone number.

HOW LONG WILL THE SPECIMENS BE STORED?

There is no time limit on how long the samples will be stored.

WHAT IF I DON'T WANT MY SAMPLES SAVED FOR FUTURE USE?

You may decide that you do not want your samples stored for future research studies. You can still participate in this study even if you make this decision. Any leftover specimens from you will be destroyed at the end of the study.

WHAT IF I AGREE TO HAVE MY SPECIMENS STORED AND THEN CHANGE MY MIND?

People always have the right to stop participating in research. If you decide that you do not want researchers to be able to use the specimens in the repository, you can contact the clinic staff. They will tell the repository that the specimens with your study code number should not be studied, and these specimens will be destroyed. If you change your mind after your specimens have already been shipped for testing, the samples that have been shipped will still be tested but your specimens still remaining in the repository will be destroyed.

WHAT ARE THE BENEFITS TO ME FROM AGREEING TO STORE SPECIMENS?

There are no direct benefits to you from storing your specimens. You may be helping people in the future from the results of studies using the stored specimens.

WHAT ARE THE RISKS TO ME FROM AGREEING TO STORE SPECIMENS?

These specimens are collected as part of this study and there is no additional risk from collecting them. They are stored by code number so there is no risk of loss of privacy.

WHAT ARE THE COSTS TO ME?

There is no cost to you for having your specimens stored.

WILL I RECEIVE ANY PAYMENT?

You will not receive any payment for providing these specimens for storage. Your samples will not be sold or directly used to produce commercial products. In the future, some of the research may help to develop new products, such as tests and drugs (commercial products). If this does happen and these tests or drugs make money, there are no plans to share that money with the people who gave the specimens.

WHAT DO I DO IF I HAVE QUESTIONS OR PROBLEMS?

For questions about this study or a research-related injury, contact:

- site insert name of the investigator or other study staff
- site insert telephone number of above

For questions about your rights as a research participant, contact:

- site insert name or title of person on the Institutional Review Board (IRB) or other organization appropriate for the site
- site insert telephone number of above

SIGNATURE PAGE FOR PARTICIPANTS OF LEGAL AGE TO PROVIDE INDEPENDENT INFORMED CONSENT

If you have read this consent form (or had it explained to you) and all of your questions have been answered, please indicate your choice and sign your name below.

____ I give my permission for storage and future testing of my specimens as discussed in this consent form (including genetic testing).

____ I give my permission for storage and future testing of my specimens as discussed in this consent form EXCEPT for genetic testing.

____ I do NOT give my permission for storage and future testing of my specimens.

______________________________	______________________________
Participant's Name (print)	Participant's Signature and Date
______________________________	______________________________
Study Staff Conducting<br>Consent Discussion (print)	Study Staff Signature and Date
______________________________	______________________________
Witness's Name (print)<br>(As appropriate)	Witness's Signature and Date

SIGNATURE PAGE FOR PARTICIPANTS BELOW LEGAL AGE TO PROVIDE INDEPENDENT INFORMED CONSENT

Participant Assent

If you have read this consent form (or had it explained to you) and all of your questions have been answered, please indicate your choice and sign your name below.

____ I give my permission for storage and future testing of my specimens as discussed in this consent form (including genetic testing).

____ I give my permission for storage and future testing of my specimens as discussed in this consent form EXCEPT for genetic testing.

____ I do NOT give my permission for storage and future testing of my specimens.

Participant's Name (print)	Participant's Signature and Date

Parent/Legal Guardian Consent

If you have read this consent form (or had it explained to you) and all of your questions have been answered, please indicate your choice and sign your name below.

____ I give my permission for storage and future testing of my child's specimens as discussed in this consent form (including genetic testing).

____ I give my permission for storage and future testing of my child's specimens as discussed in this consent form EXCEPT for genetic testing.

____ I do NOT give my permission for storage and future testing of my child's specimens.

Parent/Guardian Name (print)	Parent/Guardian Signature and Date
Study Staff Conducting Consent Discussion (print)	Study Staff Signature and Date
Witness's Name (print) (As appropriate)	Witness's Signature and Date

APPENDIX VI: SAMPLE INFORMED CONSENT FORM FOR WOMEN WHO BECOME PREGNANT WHILE ON STUDY-SUPPLIED STUDY DRUG

IMPAACT 1077HS
HAART Standard Version of the PROMISE Study
(Promoting Maternal and Infant Survival Everywhere)
Protocol Version 2.0, Dated 9 October 2012

Note to Sites: The version number and date of the protocol should be included on the first page of the consent form and the version number and date of the consent form should be included in a header or footer on each page along with page numbering in the following format: Page 1 of x, Page 2 of x, Page 3 of x.

SHORT TITLE FOR THE STUDY: PROMISE HAART-Standard Version

INTRODUCTION

This is a consent form for women who become pregnant while taking HIV medicines given to them from the PROMISE study. Because you are now pregnant, you are being asked if you want to continue taking HIV medicines from the study. This form gives information on how HIV medicines may affect you, your pregnancy, and your baby. It is important to have this information before deciding if you want to continue taking HIV medicines from the study.

The study staff will talk with you about this information. You may also talk with your own health care provider and you are free to ask questions at any time. If you choose to continue taking HIV medicines from the study, you will be asked to sign this form. You will be given a copy to keep.

WHAT DO I HAVE TO DO IF I CONTINUE TAKING HIV MEDICINES FROM THE STUDY?

There will be no change to your study visits regardless of whether you choose to continue taking HIV medicines from the study. You will continue to have study visits and tests as stated in the consent form that you signed when you first joined the study. If you are still pregnant at your last study visit, the study staff will contact you to find out the outcome of your pregnancy.

The study will not provide care related to your pregnancy or the delivery of your baby. The study also will not provide care for your baby after birth. You must arrange for this care outside the study. The study staff can tell you about places to go for this care, if you wish.

If you choose to continue taking HIV medicines from the study, the study staff will talk more with you about the medicines you are taking and make recommendations about whether to keep taking those medicines or to switch to different medicines. The study staff will also tell you if the dose of your medicines should be changed while you are pregnant.

Long-term follow-up is recommended for babies whose mothers take HIV medicines during pregnancy. The study staff will talk with you about options for long-term follow-up that may be available when your participation in this study ends.

WHAT ARE THE RISKS OF CONTINUING TO TAKE HIV MEDICINES FROM THE STUDY?

The possible risks of taking part in this study were described in the consent form that you signed when you first joined the study. This form describes additional possible risks for you and your baby from taking HIV medicines during pregnancy.

Risks to You:

- Different side effects or more severe side effects may occur in pregnant women taking HIV medicines. This may make it more difficult to take your HIV medicines. Not taking your medicines as directed could cause the medicines to not work on the HIV in your body.
- The amount of HIV medicine in the blood may change during pregnancy. Because of this, the amount of medicine in your body may be decreased and the medicines may not work as well as usual. This could also cause the HIV in your body to become resistant. When resistance occurs, a medicine no longer works against HIV, which can limit the choices of HIV medicines that a person can take in the future.
- It is not known if some risks of pregnancy might be made worse by HIV medicines, possibly resulting in death.

Risks to Your Baby:

- It is not known if some HIV medicines may cause babies to be born early or dead.
- It is not known if some HIV medicines may cause babies to be sick or have birth defects. Not all birth defects are seen at birth. Some birth defects are seen later as the baby grows.

The World Health Organization recommends the use of several HIV medicines during pregnancy that are available through the study, including zidovudine (ZDV), lamivudine (3TC), tenofovir (TDF), and lopinavir-ritonavir (LPV-RTV). In the US, only ZDV is approved by the Food and Drug Administration to decrease the risk of passing HIV from mother to baby. The US Public Health Service recommends that women take ZDV with other HIV medicines to decrease the risk of passing HIV from mother to baby.

In the places where the PROMISE study is being done, it recommended that women take a combination of at least three HIV medicines during pregnancy to try to keep their babies from getting HIV during pregnancy and delivery. If you choose not to continue taking HIV medicines from the study while you are pregnant, it is important that you take HIV medicines from another program or provider outside the study to decrease the risk of passing HIV to your baby.

BREASTFEEDING

Breastfeeding is not recommended for HIV-infected women where safe formula feeding is available. HIV can be passed to babies through breast milk and taking HIV medicines during breastfeeding cannot be guaranteed to protect against this. Babies may also receive some amounts of HIV medicines taken by their mothers through breast milk. It is not known whether this may cause any harm to babies.

WHAT ARE THE BENEFITS OF CONTINUING TO TAKE HIV MEDICINES FROM THE STUDY?

The possible benefits of taking part in this study were described in the consent form that you signed when you first joined the study. This form describes additional possible benefits for you and your baby from taking HIV medicines during pregnancy.

HIV medicines, whether given to you from the study or obtained from outside the study, can decrease risk of passing HIV from mother to baby. These medicines are used throughout the world for this purpose. Information learned from this study may help others who have HIV in the future.

WHAT OTHER CHOICES DO I HAVE BESIDES CONTINUING TO TAKE HIV MEDICINES FROM THE STUDY?

Instead of continuing to take HIV medicines from the study, you have the choice of taking HIV medicines from another program or provider outside the study. The study staff will talk with you about these choices, their risks and benefits. You should also talk with your own health care provider about these choices.

WHAT ABOUT CONFIDENTIALITY?

The consent form that you signed when you first joined the study explained the efforts that will be made keep information about you and your participation in this study confidential. There will be no change to this regardless of whether you continue to take HIV medicines from the study.

WHAT ARE THE COSTS TO ME?

There are no additional costs to you as a result of continuing to take HIV medicines from the study while you are pregnant. If you take HIV medicines from another program or provider outside the study, you or your health insurance will need to pay for the medicines, unless the medicines are available free of charge. The study cannot pay for medicines obtained from other programs or providers.

As stated above, this study will not cover any costs related to your pregnancy, delivery of your baby, or care for your baby.

WILL I RECEIVE ANY PAYMENT?

The consent form that you signed when you first joined the study explained the reimbursement you will receive for study visits. There will be no change to this regardless of whether you continue to take HIV medicines from the study.

WHAT HAPPENS IF I AM OR MY BABY IS INJURED?

If you or your baby are injured as a result of you being in this study — including as a result of taking HIV medicines from the study — you will both be given immediate treatment for your injuries [sites: add local information regarding treatment for injury]. There is no program for compensation either through this institution or the US National Institutes of Health (NIH). You will not be giving up any of your legal rights by signing this consent form.

WHAT ARE MY RIGHTS AS A RESEARCH PARTICIPANT?

Continuing to take part in this study, and to take HIV medicines from the study, is completely voluntary. You may choose not to do this or to leave the study at any time. You will be treated the same no matter what you decide.

We will tell you about any new information from this study or other studies that may affect your health, welfare, or willingness to continue taking HIV medicines from the study. We will also tell you about new information that may affect your willingness to stay in the study.

WHAT DO I DO IF I HAVE QUESTIONS OR PROBLEMS?

For questions about this study or a research-related injury, contact:
- site insert name of the investigator or other study staff
- site insert telephone number of above

For questions about your rights as a research participant, contact:
- site insert name or title of person on the Institutional Review Board (IRB) or other organization appropriate for the site
- site insert telephone number of above

SIGNATURE PAGE FOR PARTICIPANTS OF LEGAL AGE TO PROVIDE INDEPENDENT INFORMED CONSENT

If you have read this consent form (or had it explained to you), all of your questions have been answered, and you agree to continue taking HIV medicines from the study during your pregnancy, please sign your name below.

______________________________	______________________________
Participant's Name (print)	Participant's Signature and Date
______________________________	______________________________
Study Staff Conducting<br>Consent Discussion (print)	Study Staff Signature and Date
______________________________	______________________________
Witness's Name (print)<br>(As appropriate)	Witness's Signature and Date

SIGNATURE PAGE FOR PARTICIPANTS BELOW LEGAL AGE TO PROVIDE INDEPENDENT INFORMED CONSENT

Participant Assent

If you have read this consent form (or had it explained to you), all of your questions have been answered, and you agree to continue taking HIV medicines from the study during your pregnancy, please sign your name below.

______________________________	______________________________
Participant's Name (print)	Participant's Signature and Date

Parent/Legal Guardian Consent

If you have read this consent form (or had it explained to you), all of your questions have been answered, and you agree for your child (named above) to continue taking HIV medicines from the study during her pregnancy, please sign your name below.

______________________________	______________________________
Parent/Guardian Name (print)	Parent/Guardian Signature and Date
______________________________	______________________________
Study Staff Conducting Consent Discussion (print)	Study Staff Signature and Date
______________________________	______________________________
Witness's Name (print) (As appropriate)	Witness's Signature and Date
